# Experimental verification of the error minimization theory using non-standard genetic codes constructed in vitro

**DOI:** 10.7554/eLife.111164

**Published:** 2026-07-13

**Authors:** Ryota Miyachi, Norikazu Ichihashi

**Affiliations:** 1 Department of Life Science, Graduate School of Arts and Sciences, The University of Tokyo Tokyo Japan; 2 Komaba Institute for Science, The University of Tokyo Tokyo Japan; 3 Research Center for Complex Systems Biology, Universal Biology Institute, The University of Tokyo Tokyo Japan; Centre for Cellular and Molecular Biology India; University of Cambridge United Kingdom

**Keywords:** genetic code, tRNA, reconstituted system, cell-free, mutation, *E. coli*

## Abstract

All living systems use an almost identical standard genetic code (SGC), in which 20 amino acids are assigned non-randomly. According to the error minimization theory, amino acids are arranged to minimize the mutational effect on protein function, while experimental verification remains limited. Here, we constructed 10 non-standard genetic codes (non-SGCs) in vitro by reassigning three amino acids (Ala, Ser, and Leu) in vacant codons of the minimal genetic code consisting of 21 tRNAs. Most of these non-SGCs have a higher cost of amino acid replacement than the SGC, calculated based on three amino acid properties: polar requirement (PR), molecular volume (MV), and hydropathy index (HI). The protein function of three reporter genes expressed using these non-SGCs decreased similarly when random mutations were introduced into the genes, implying that the effect of mutations was similar across all the non-SGCs tested here. This result provides direct experimental evidence that mutational robustness does not significantly change in individual reporter protein activity within the range of mutational cost tested in this study (Cost_PR_: 5.29–5.77, Cost_MV_: 1848–2348, and Cost_HI_: 3.27–5.10), which covers approximately 18.4% (PR), 37.6% (MV), and 50.8% (HI) of the possible cost range achievable among one million randomly-generated genetic codes.

## Introduction

**Figure 1.**
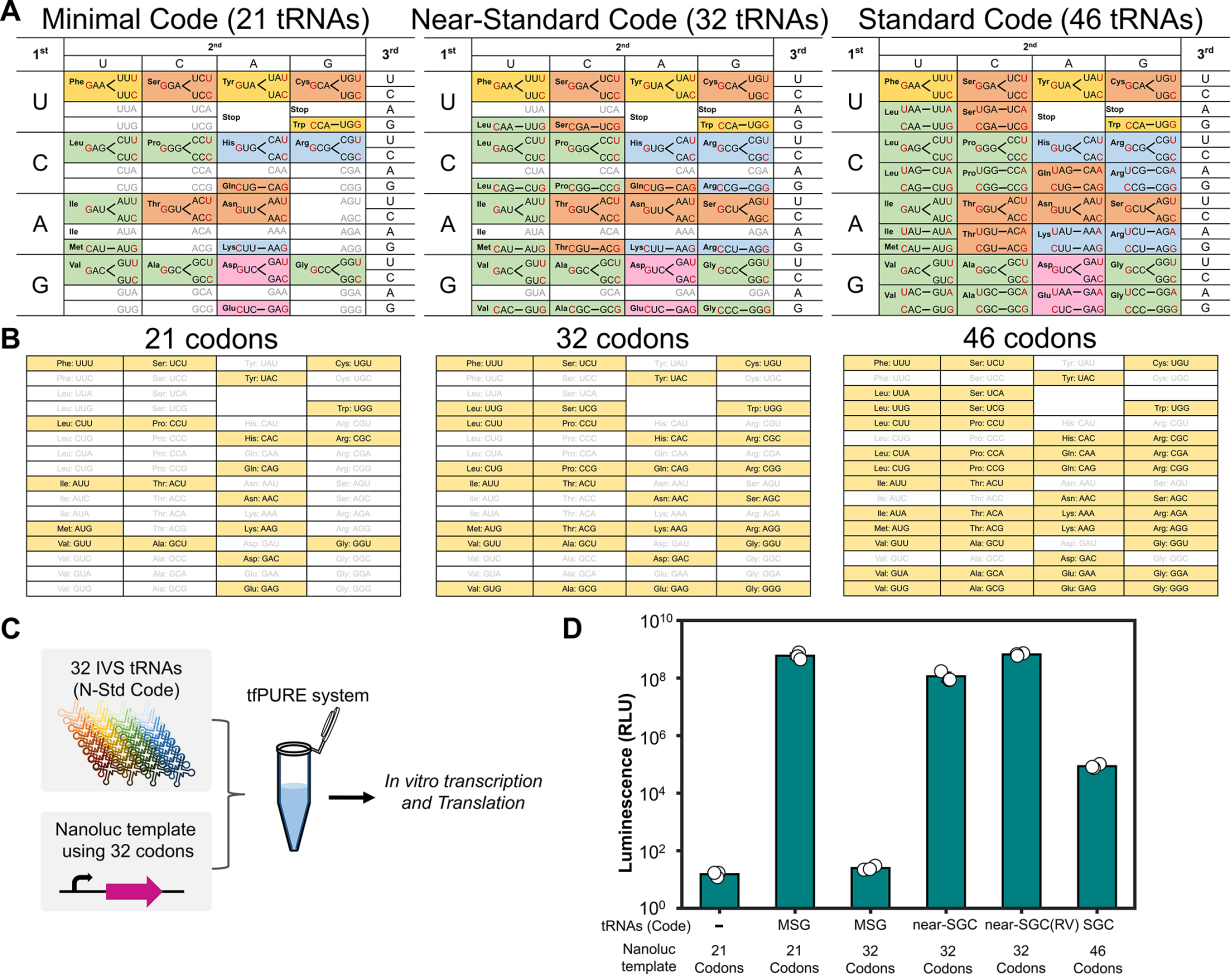
In vitro construction of minimal, near-standard, and standard genetic codes (SGCs). (**A**) Anticodons of tRNAs and their corresponding codon assignments in the minimal genetic code (MGC), the near-SGC, and the SGC. Each codon is colored according to the physicochemical properties of the assigned amino acid: hydrophobic (green), aromatic (yellow), polar uncharged (orange), basic (blue), and acidic (pink). In each box, the anticodons of tRNAs (left) and the corresponding codons (right) are shown. (**B**) Codon sets used for reporter genes. The 21 codons contain only codons that are usable for MGC. Reporter genes composed of these codons were used in D and the subsequent experiments using non-SGCs. The 32 and 46 codons contain codons that are usable for near-SGC and SGC, respectively. Reporter genes composed of these codons were used in D. (**C**) Schematic of the translation assay. Reporter genes (NanoLuc, 1 nM) consisting of the 21, 32, or 46-codon were translated in a customized reconstituted translation system lacking endogenous tRNAs (tRNA-free PURE system [tfPURE]) supplemented with in vitro-synthesized tRNAs corresponding to MGC, near-SGC, or SGC (IPEN tRNA at 100 ng/µL; all other tRNAs at 12 ng/µL), and T7 RNA polymerase (0.42 U/µL) at 30 °C for 16 hr. (**D**) NanoLuc activity after incubation. In near-SGC (RV), two tRNAs (tRNA^Val^_CAC_ and tRNA^Arg^_CCU_) were increased to 100 ng/µL. Each dot represents an independent experiment (n=3). Bars indicate mean values, and error bars represent SDs. Statistical comparisons in (**D**) were performed using one-way ANOVA followed by Tukey’s post hoc test on NanoLuc activity; major comparisons are summarized in Appendix 1—table 8.

The genetic code is the set of rules for the translation of nucleotide sequences in mRNA into amino acid sequences in proteins. All known living organisms use a nearly identical genetic code, referred to as the standard genetic code (SGC), representing a universal mechanism shared by almost all extant living systems on Earth (Crick, 1968; Koonin and Novozhilov, 2017; Woese, 1965; Woese et al., 1966). In SGC, 20 amino acids and stop codons are nonrandomly assigned to 64 codons; amino acids with similar physicochemical properties are assigned to neighboring codons (see Figure 1A, SGC; Koonin and Novozhilov, 2017; Koonin and Novozhilov, 2009). The origin and reason for this non-random codon organization remain a major mystery in biology (Crick, 1968; Di Giulio, 2005; Koonin and Novozhilov, 2017; Koonin and Novozhilov, 2009).

**Figure 1—figure supplement 1.**
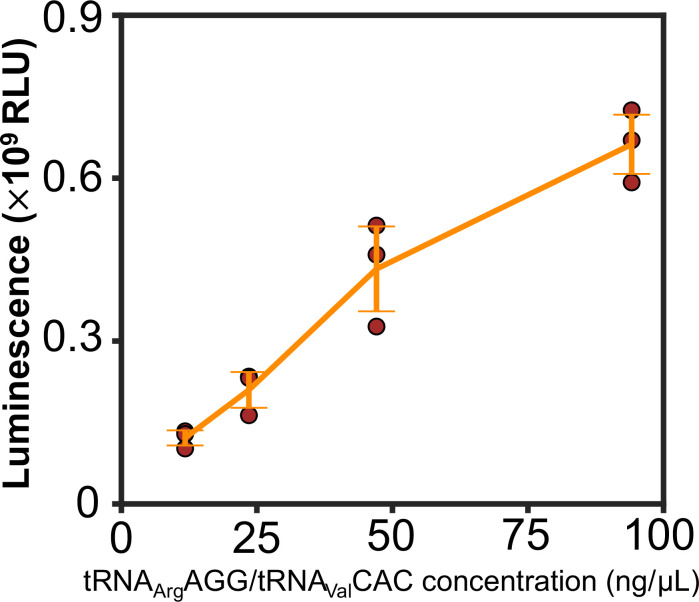
Effect of tRNA^Val^_CAC_ and tRNA^Arg^_AGG_ concentrations on NanoLuc translation using near-SGC. The concentrations of both tRNAs were increased simultaneously at equal ratios. Each dot represents the results of three technical replicates, and error bars represent SDs.

**Figure 1—figure supplement 2.**
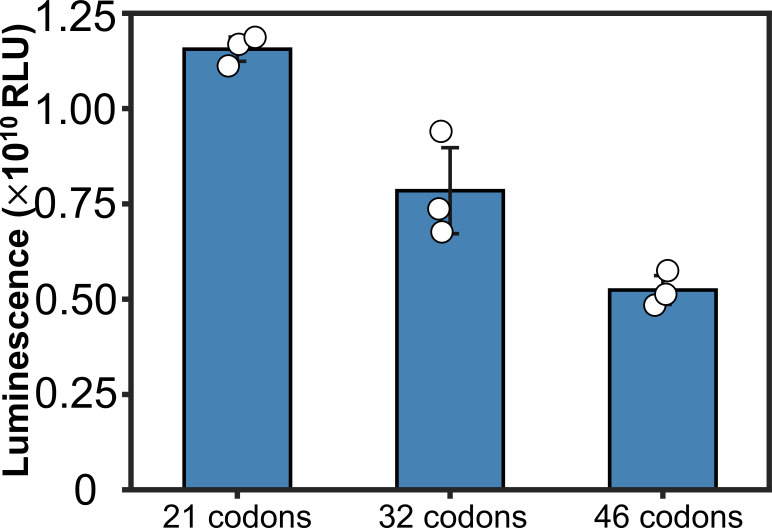
Translation of each NanoLuc templates using Native *Escherichia coli* tRNAs. Translation of NanoLuc templates encoded with 21, 32, or 46 codons using native *E. coli* tRNAs (600 ng/µL) in the tfPURE system.

One explanation for this nonrandomness in SGC is mutational robustness. According to the error minimization theory, amino acid arrangement in SGC was selected to minimize the functional impact of amino acid substitutions caused by single-nucleotide mutations or translational errors (Freeland et al., 2003; Freeland and Hurst, 1998; Haig and Hurst, 1991). Several theoretical studies have estimated the possible cost of amino acid replacement (referred to as mutational cost in this study) based on the physicochemical properties of amino acids. These analyses consistently demonstrated that SGC exhibits an exceptionally low mutational cost compared with randomly generated genetic codes (Buhrman et al., 2013; Freeland and Hurst, 1998; Gilis et al., 2001; Goodarzi et al., 2004; Haig and Hurst, 1991; Omachi et al., 2023), although some genetic codes still have a lower cost than SGC (Błażej et al., 2018; Novozhilov et al., 2007; Wnętrzak et al., 2018). An alternative explanation for the non-randomness of SGC is a by-product of genetic code expansion (Massey, 2016; Massey, 2008). According to this explanation, the arrangement of SGC does not confer a selective advantage over other genetic codes. Despite these many theoretical studies, experimental verification is still limited.

A fundamental obstacle to the experimental verification of the role of genetic code arrangement in mutational robustness is the lack of variation in genetic codes in nature; all extant organisms use a nearly identical genetic code (Massey, 2015; Shulgina and Eddy, 2021). Recently, *Escherichia coli* with designed genetic codes have been developed by removing specific stop codons and rarely used sense codons from the genome (Fredens et al., 2019; Lajoie et al., 2013; Nyerges et al., 2023; Robertson et al., 2025; Robertson et al., 2021; Zürcher et al., 2022) and reassigning them to non-canonical or alternative amino acids through the introduction of orthogonal tRNA-aminoacyl-tRNA synthetase (aaRS) pairs (Costello et al., 2024; Shandell et al., 2021). However, these approaches based on living cells require extensive genome engineering, and thus, the number of codons that can be reassigned remains limited.

An alternative approach for genetic code alteration is in vitro reconstitution (Hibi et al., 2020; Miyachi et al., 2025; Shimizu et al., 2001). In this approach, the components required for translation, such as tRNAs and aaRSs, are individually prepared and supplied, allowing simultaneous reassignment of multiple codons. A representative platform for such reconstitution is the PURE system (Shimizu et al., 2001), a reconstituted cell-free translation system composed of purified translation components, including ribosomes, translation factors, aaRSs, amino acids, and energy-regeneration components. In particular, a tRNA-free PURE (tfPURE) system (Miyachi et al., 2022), in which endogenous tRNA activity is minimized and defined tRNA sets are supplied externally, enables genetic codes to be reconstructed by controlling the supplied tRNAs. To date, several genetic codes have been reconstituted in vitro, including minimal genetic codes (MGCs) composed of 21 tRNAs that mimic or are derived from SGC, and those incorporating non-canonical amino acids (Forster et al., 2003; Iwane et al., 2016; Jones et al., 2025; McFeely et al., 2023). Translation of peptides and proteins using these reconstituted genetic codes has also been demonstrated (Calles et al., 2019; Fujino et al., 2020; Hibi et al., 2020; Iwane et al., 2016; Miyachi et al., 2025). However, reconstitution of elaborately arranged genetic codes and protein translation by them remains challenging.

In this study, to assess the effect of genetic code arrangement on mutational robustness, we constructed non-SGCs in vitro and compared the effect of mutations on the function of translated proteins. We first established a MGC, composed of 21 tRNAs with vacant codons, which allows multiple alternative codon assignments to be introduced under otherwise comparable translation conditions. By introducing tRNA variants into these vacant codons, we then constructed 10 non-SGCs with distinct costs of amino acid replacement, along with a near-SGC. Using these genetic codes, we translated reporter gene libraries carrying random mutations and quantified the resulting protein activities. Random mutations decreased reporter protein function at similar levels across all genetic codes examined, implying that alterations of the genetic code within the ranges explored in this study have no significant effect on mutational robustness of individual protein activity.

## Results

### Construction of minimal and standard genetic codes

Before constructing non-SGCs, we first constructed a MGC using 21 tRNAs (20 for 20 amino acids and one for formyl-Met) according to a previously reported method (Fujino et al., 2020; Hibi et al., 2020; Figure 1A, left). In this MGC, we used tRNAs with G at the first position of the anticodon for 15 amino acids (Phe, Ser, Tyr, Cys, Leu, Pro, His, Arg, Ile, Thr, Asn, Val, Ala, Asp, and Gly), which can decode codons with C or U at the third position through wobble base pairing (Hibi et al., 2020). We also used tRNAs with C at the first position of the anticodon for 5 amino acids (Trp, Gln, Met, Lys, and Glu), which can decode codons with G at the third position. The expected correspondences between anticodons and the codons they decode are represented as lines in Figure 1A (left three letters in each box indicate the anticodon of the tRNA, and the right ones indicate the codon).

To ensure fair comparisons, we also constructed genetic codes close to the SGC using the same method (i.e. using unmodified tRNAs). Previously, a genetic code approximating the SGC was constructed in vitro using 32 tRNAs for peptide synthesis (Iwane et al., 2016). Following this method, we introduced 11 additional tRNAs (Leu_CAA, Ser_CGA, Leu_CAG, Pro_CGG, Arg_CCG, Ser_GCU, Thr_CGU, Arg_CCU, Val_CAC, Ala_CGC, and Gly_CCC) into the MGC (Figure 1A, middle). We refer to this as the near-SGC. Although the near-SGC mimics the SGC in most of the codons, it does not cover codons with A at the third nucleotide position (NNA). We therefore further introduced 14 tRNAs with UNN anticodons that are expected to decode NNA codons to construct SGC consisting of a 46-tRNA set (Figure 1A, right).

We next examined the translation efficiency of the reconstructed MGC, near-SGC, and SGC. Translation was performed in a custom-made tfPURE system (Miyachi et al., 2025), supplemented with 21 tRNAs for MGC, 32 tRNAs for near-SGC, or 46 tRNAs for SGC. As a reporter protein, we used the NanoLuc luciferase, originally derived from deep-sea shrimp (Hall et al., 2012). For MGC, the NanoLuc sequence was designed to use only the 21 corresponding codons (Figure 1B, 21 codons). For near-SGC and SGC, the NanoLuc coding sequences were designed so that the codons available in each genetic code were used with minimal differences in codon counts, while preserving the amino acid sequence (Figures 1B, 32 codons and 46 codons). After incubation at 30 °C for 16 hr, luminescence was measured as an indicator of translation efficiency. Translation using near-SGC exhibited lower luminescence (1.2×10^8^; Figure 1D, near-SGC) than that using MGC (6.0×10^8^; MGC, 21 codons). To improve translation efficiency with near-SGC, we focused on two tRNA concentrations (tRNA^Val^_CAC_ and tRNA^Arg^_CCU_), which were suggested to have low activities in a previous study (Iwane et al., 2016). Upon increasing the concentrations of these two tRNAs, the translation increased up to 100 ng/μL (Figure 1—figure supplement 1). At 100 ng/μL for each tRNA, the translation efficiency of near-SGC was increased to a level comparable to that of MGC (6.6×10^8^; Figure 1D, near-Std [RV]). The increased concentrations of these two tRNAs were used in all the subsequent experiments. The translation level of SGC, which uses 46 codons with 46 tRNAs, was approximately 100-fold lower (8.6×10^4^; Figure 1D), indicating that the additional tRNAs corresponding to NNA codons may not function as efficiently as other tRNAs. This inefficiency is probably due to the lack of chemical modifications in tRNAs used here, as the anticodon of the native tRNAs that decode NNA are frequently modified in the cell (El Yacoubi et al., 2012; Masuda and Hou, 2024). Such chemical modifications may be required for efficient translation of the NNA codon (Cui et al., 2015; Madore et al., 1999; Tamura et al., 1992).

We also examined whether the lower translation efficiency of the 46-codon NanoLuc template could be explained by sequence-dependent effects, such as codon context or mRNA structure. When the 21-, 32-, and 46-codon NanoLuc templates were translated using native *E. coli* tRNAs in the tfPURE system (Figure 1—figure supplement 2), the 46-codon template showed lower activity than the 21- and 32-codon templates; however, this difference was within approximately twofold. Accordingly, we decided to use only the 32 codons used in near-SGC (i.e. excluding NNA codons) in the subsequent construction of non-SGCs.

### Test of the availability of each vacant codon for Ala, Ser, and Leu

We next aimed to construct multiple non-standard genetic codes (non-SGCs) by introducing tRNAs with altered anticodon sequences into the vacant codons of MGC, excluding NNA codons. Currently, anticodon sequences of tRNAs can be altered only for Ala, Ser, and Leu while maintaining recognition by their respective aaRSs because the aaRSs for these amino acids do not recognize the anticodon sequence (Asahara et al., 1993; Francklyn and Schimmel, 1989; Sampson and Saks, 1993). Therefore, we decided to design non-SGCs by reassigning Ala, Ser, and Leu to 10 vacant codons (UUG, UCG, CUG, CCG, CGG, ACG, AGC, GUG, GCG, and GGG) in MGC. Note that one vacant codon (AGG) was assigned to the original amino acid (Arg) because this codon is already distant from the codon used for Arg (CGC) in MGC and does not need to be reassigned.

**Figure 2.**
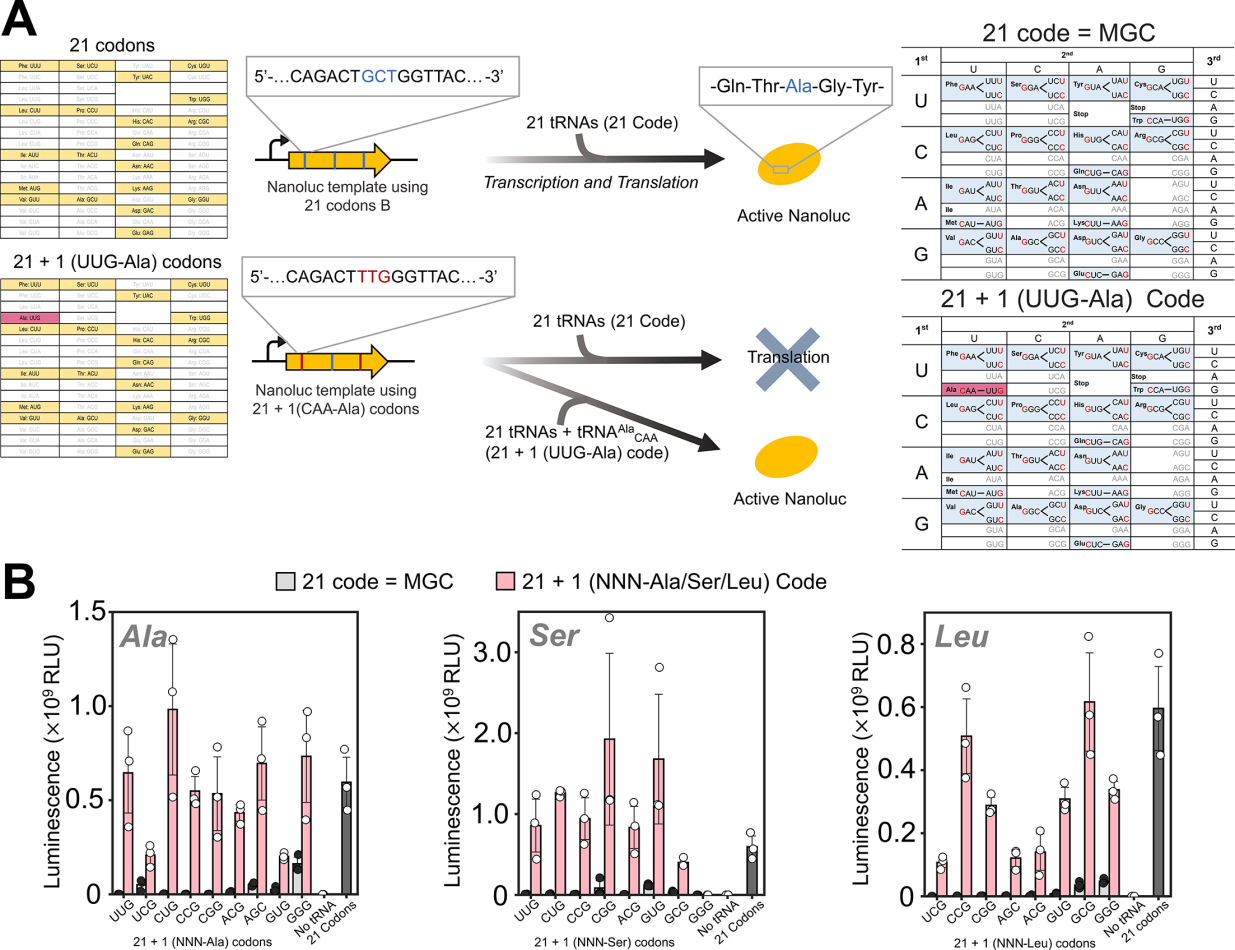
Reassignment experiments to test the availability of 10 vacant codons for Ala, Ser, and Leu. (**A**) Schematic illustration of reassignment experiments. Translation with the original MGC and NanoLuc template is shown at the top for comparison. An example of Ala reassignment to the UUG codon is shown at the bottom. In this example, three Ala codons in the NanoLuc sequence were replaced with one type of vacant codon (e.g. UUG), generating a 21+1 (UUG-Ala) codon set. Similar reassignment experiments were performed for three amino acids (Ala, Ser, and Leu) and nine vacant codons. Specifically, two Ala codons (Ala16 and Ala120), three Ser codons (Ser31, Ser49, and Ser150), or four Leu codons (Leu32, Leu67, Leu144, and Leu170) were replaced. (**B**) NanoLuc translation results for each codon reassignment experiment. Translation reactions were performed in tfPURE supplemented with a 21-tRNA mixture (600 ng/µL), one tRNA variant (12 ng/µL each), and each NanoLuc template (1 nM) that contains 2–4 of a corresponding codon to be tested (21+1 NNN-Ala/Ser/Leu codons). Reactions were incubated at 30 °C for 16 h, after which NanoLuc activity was measured. As a control, translation reactions lacking the additional tRNA variant were conducted (21 code, gray bars) and compared to the data with the additional tRNA (21+1 code, pink bars). Additional controls included translation without any tRNA (no tRNA) and translation using MGC with NanoLuc templates encoded by the original 21 codons (21 codons), both shown for comparison. Each dot represents three technical replicates, and error bars represent SDs. For each template, NanoLuc activity in the 21-code and corresponding 21+1 code conditions was compared using Welch’s t-test on luminescence. Statistical results are summarized in Appendix 1—table 9.

We tested whether each of the 10 vacant codons is usable for Ala, Leu, and Ser when the corresponding tRNA variant with an altered anticodon is introduced (Figure 2A). To test this, we constructed a total of 25 genetic codes (nine for Ala, eight for Ser, and eight for Leu) by introducing one tRNA variant with an altered anticodon into MGC (referred to as 21+1) and also designed 25 corresponding NanoLuc template DNAs, in which some codons for Ala, Ser, and Leu (two for Ala, three for Ser, and four for Leu) were replaced with the corresponding vacant codons from the 21-codon set shown in Figure 1B. For example, when assigning Ala to the UUG codon (Figure 2A, bottom), two GCU codons used for Ala in the template DNA were changed to UUG codons.

**Figure 2—figure supplement 1.**
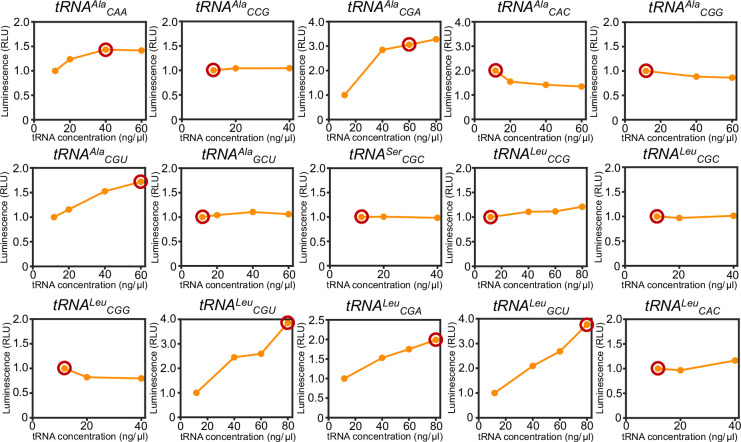
Optimization of the concentration of each anticodon variant tRNA. For 15 of the 25 anticodon variant tRNAs that exhibited relatively low translational activity, the effect of increasing tRNA concentration on translation efficiency was examined. Translation assays were performed under the same conditions as described in Figure 2. The original concentration of each tRNA variant was 12 ng/µL. The concentrations selected for use in subsequent experiments are indicated by red circles.

Translation reactions were performed in tfPURE supplemented with 21 tRNAs alone (21 code, equivalent to MGC) or 21 tRNAs plus one additional tRNA variant for Ala, Ser, and Leu (21+1 code) with the corresponding NanoLuc template (1 nM) at 30 °C for 16 hr. NanoLuc activity was low when using only 21 tRNAs (Figure 2B, gray bars, 21 code), but increased when the corresponding tRNA variant was supplied (pink bars, 21+1 code) for most of the tested codons. An exception was observed when Ser was assigned to the GGG codon, in which NanoLuc activity did not increase with 21+1 code for reasons that remain unclear. These results demonstrate that the nine tested vacant codons (i.e. excluding GGG) can be reassigned to the three amino acids, allowing the construction of up to 3^9^=19,683 types of non-SGCs, or approximately 20,000 codes. For GGG, we retained the original glycine assignment in all subsequent experiments. We further optimized the concentration of each additional tRNA variant, particularly for those that exhibited lower translation efficiency (Figure 2—figure supplement 1). Based on these results, the concentrations of the following tRNAs were increased to the indicated concentrations in subsequent experiments: tRNA^Ala^_CAA_ (40 ng/µL), tRNA^Ala^_CGA_ (60 ng/µL), tRNA^Ala^_CGU_ (60 ng/µL), tRNA^Leu^_CGU_ (80 ng/µL), tRNA^Leu^_CGA_ (80 ng/µL), and tRNA^Leu^_GCU_ (80 ng/µL).

### Design of non-standard genetic codes

**Figure 3.**
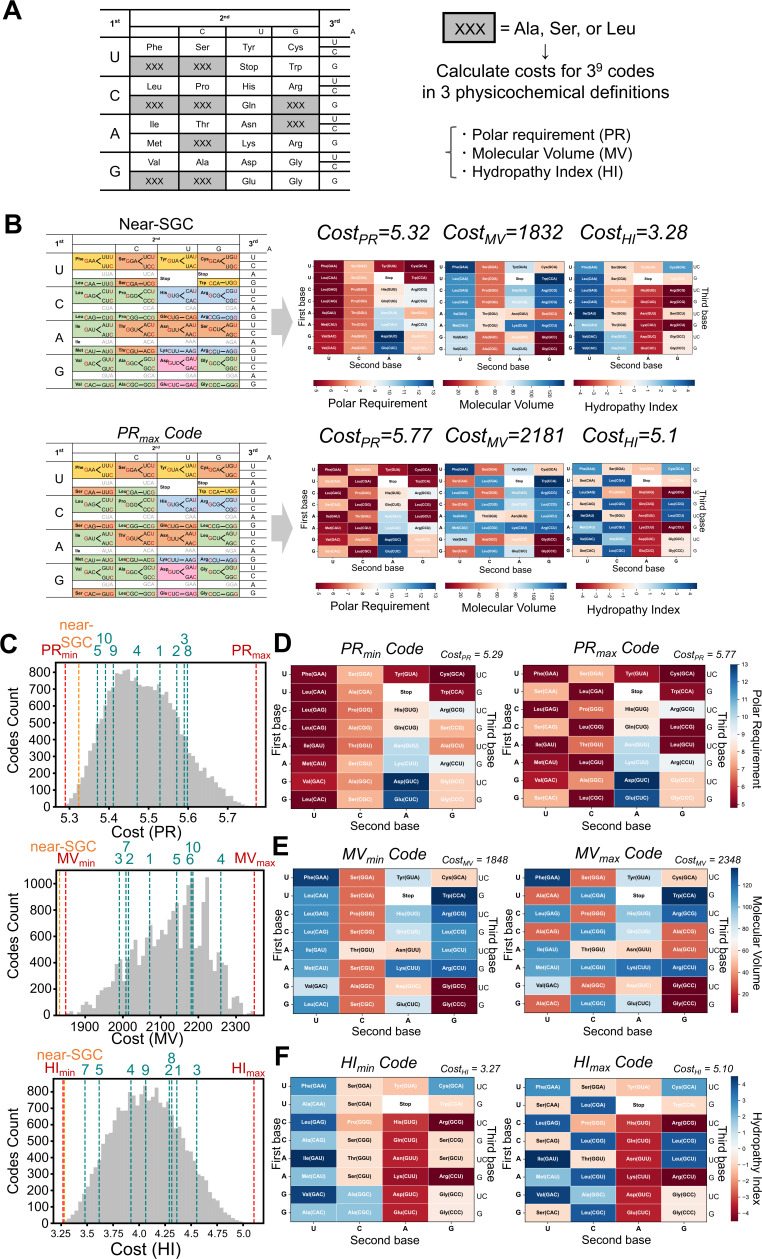
Distribution of mutational costs of reassigned genetic codes. (**A**) Calculation method of mutational costs for each genetic code based on three physicochemical properties of amino acids. The average change in each of the three physicochemical properties of amino acids upon single-nucleotide substitutions from the 21 codons was calculated (see Methods for details). In the reassigned genetic codes analyzed here, one of three amino acids (Ala, Ser, or Leu) was assigned to each of the nine vacant codons shown in gray, and the costs were calculated for all possible reassignment combinations. (**B**) Representative comparison of the near-SGC and PR_max_ code. Codon assignment schemes are shown on the left, and heatmap representations of the assigned amino acid values for polar requirement (PR), molecular volume (MV), and hydropathy index (HI) are shown on the right. The corresponding Cost_PR_, Cost_MV_, and Cost_HI_ values are indicated above each heatmap. (**C**) Distributions of mutational costs for each physicochemical property of amino acids. Dashed lines indicate the cost values of 10 genetic codes selected for experimental construction. Red dashed lines indicate the minimum and maximum cost values for each cost definition, and orange lines indicate the cost values of near-SGC. (**D, E, F**) Genetic codes exhibiting the minimum and maximum mutational costs based on PR (**D**), MV (**E**), and HI (**F**). The physicochemical values of amino acids assigned to each codon are shown as heatmaps.

We next designed non-SGCs. By assigning Ala, Ser, or Leu to the nine vacant codons (gray boxes in Figure 3A), a total of 19,683 (=3^9^) possible genetic codes can be generated. The objective of this study is to evaluate how the arrangement of the genetic code influences the impact of point mutations on protein function. To guide the selection of genetic codes to construct, we defined three types of mutational cost for each genetic code based on three physicochemical properties of amino acids, polar requirement (PR), molecular volume (MV), and hydropathy index (HI), according to previous studies (Freeland and Hurst, 1998; Haig and Hurst, 1991). PR reflects polarity-related interactions of amino acids and has been used as a classical measure of amino acid similarity in error minimization analyses. MV represents side-chain size and steric volume, which could affect protein packing and structural stability, whereas HI reflects hydrophobicity, which could be closely related to protein folding or hydrophobic core formation. The mutational costs, three values (Cost_PR_, Cost_MV_, and Cost_HI_) associated with each genetic code, represent the expected average negative effect of a single nucleotide substitution in a target gene on the protein function, estimated from the change in each physicochemical property of the substituted amino acid. To make the design of non-SGCs more explicit, we show one representative non-SGC together with the near-SGC in Figure 3B. This comparison illustrates how assignment of Ala, Ser, or Leu to the vacant codon boxes changes the three mutational cost metrics, Cost_tPR_, Cost_MV_, and Cost_HI_.

**Figure 3—figure supplement 1.**
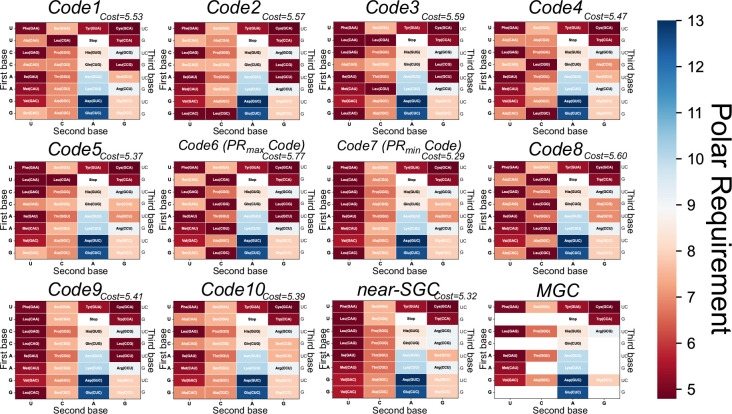
Polar requirement (PR) values of amino acids assigned in the constructed non-standard genetic codes (non-SGCs). The experimentally constructed non-SGCs and their corresponding mutational costs are shown. For each genetic code, the PR values of amino acids assigned to individual codons are displayed as heatmaps. Near-SGC and MGC are shown for comparison.

**Figure 3—figure supplement 2.**
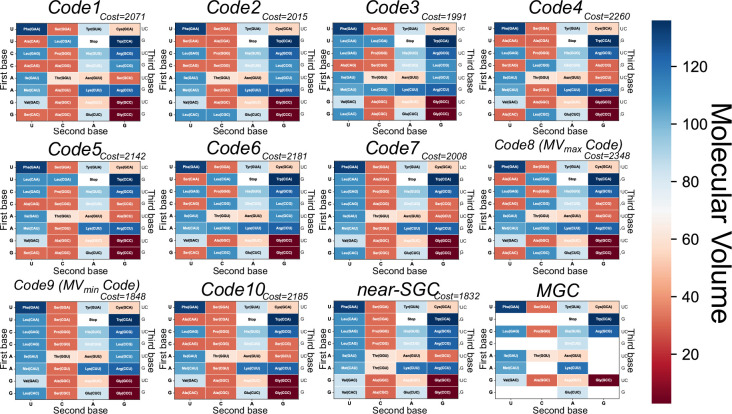
Molecular volume (MR) values of amino acids assigned in the constructed non-standard genetic codes (non-SGCs). The experimentally constructed non-SGCs and their corresponding mutational costs are shown. For each genetic code, the MR values of amino acids assigned to individual codons are displayed as heatmaps. Near-SGC and MGC are shown for comparison.

**Figure 3—figure supplement 3.**
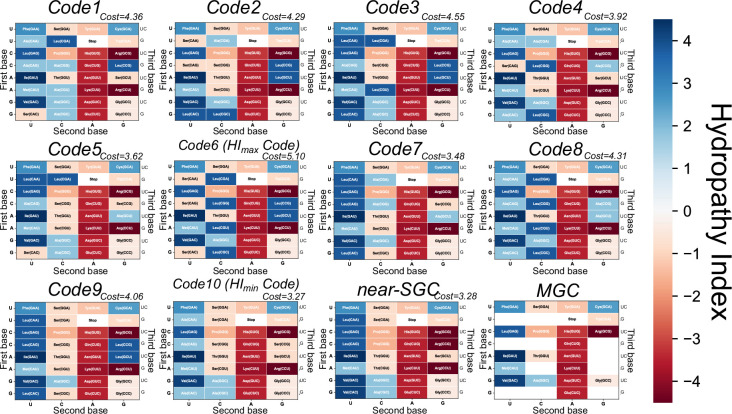
Hydropathy index (HI) values of amino acids assigned in the constructed non-standard genetic codes (non-SGCs). The experimentally constructed non-SGCs and their corresponding mutational costs are shown. For each genetic code, the HI values of amino acids assigned to individual codons are displayed as heatmaps. Near-SGC and MGC are shown for comparison.

The three types of mutation costs based on PR, MV, and HI were calculated for each of the 19,683 (=3^9^) possible genetic codes as follows. First, we considered a virtual protein-encoding DNA sequence using the 21 codons shown in Figure 1B (left) at equal frequencies. We then enumerated the set of codons reachable from each codon via single-nucleotide substitutions and calculated the average change in each physicochemical property of amino acids for each genetic code. In this calculation, mutation biases, including the relative frequencies of transitions and transversions, were incorporated as weighting factors. When mutations produced NNA codons, which are vacant in the genetic codes used here, their substitutions were neglected. The resulting average physicochemical change was defined as the mutational cost for the target genetic code. According to this calculation, a smaller mutational cost indicates that single-nucleotide substitutions lead to smaller changes in the corresponding amino acid properties and thus expectedly smaller changes in protein function, reflecting higher robustness against mutations.

The distributions of mutational costs calculated for each physicochemical property (Cost_PR_, Cost_MV_, and Cost_HI_) are shown in Figure 3C. The near-SGC (orange dashed lines) was located in the low-cost region of the distributions for all three properties, consistent with previous theoretical studies (Freeland and Hurst, 1998; Haig and Hurst, 1991). We next selected genetic codes for construction and experimental evaluation. First, we selected the genetic codes exhibiting the maximum and minimum costs for each physicochemical property (Figure 3B, red dashed lines; PR_min/max_, MV_min/max_, and HI_min/max_). These selected genetic codes are shown in Figure 3D–F, colored according to the physicochemical values of the assigned amino acids. The genetic codes of the minimum costs (PR_min_, MV_min_, and HI_min_) exhibit relatively ordered arrangements of the physicochemical values, whereas those with maximum costs (PR_max_, MV_max_, and HI_max_) exhibit relatively disordered arrangements. Because the PR_max_ and HI_max_ codes were identical, these five extreme genetic codes were selected for construction. Second, we selected five additional genetic codes representing various points across the cost distributions for all three properties (green dashed lines). These 10 non-SGCs (shown in Figure 3—figure supplements 1–3) were constructed by introducing the corresponding additional tRNA sets into MGC and used in subsequent experiments.

### Preparation and analysis of random mutation libraries of reporter genes

To compare functional robustness against mutations across different genetic codes, we prepared randomly mutagenized DNA libraries of three reporter genes, β-galactosidase (GAL), firefly luciferase (Luc), and mStayGold (Ando et al., 2024; mSG), from the original sequences composed of only the 21 codons shown in Figure 1B (left). DNA libraries were prepared through a three-step PCR procedure (Figure 4—figure supplement 1A). In the first PCR, random mutations were introduced by error-prone PCR using Taq DNA polymerase in the presence of Mn^2+^. The mutation rate was tuned by varying the Mn^2+^ concentration across five levels: 10, 50, 100, 250, and 350 µM. For comparison, we also performed standard PCR using a high-fidelity polymerase (KOD Plus Neo) to prepare a low-mutation library. In the second PCR, a HiBiT tag was attached to the C-terminus using the high-fidelity polymerase for quantitation of translation levels. In the third PCR, Illumina sequencing adapters and unique barcode sequences were added using the high-fidelity polymerase. The products of the second PCR were used directly for translation experiments, whereas the products of the third PCR were subjected to Illumina sequencing to quantify mutation rates and characterize the resulting libraries.

Mutation frequencies at each position were calculated from the sequencing data obtained. The simple average of these values across all positions was defined as the mean per-base mutation rate. When using Taq DNA polymerase, the error rates increased monotonically with increasing Mn^2+^ concentration, ranging from 1.2×10^–3^ to 6.9×10^–3^ (Figure 4—figure supplement 1B). In contrast, when using the high-fidelity polymerase, the error rates were much lower (ranging from 3.0×10^–4^ to 4.0×10^–4^). The mutations observed with the high-fidelity polymerase are thought to have been caused by sequencing errors because the rate is consistent with the reported sequencing error rate (Schirmer et al., 2016) and also the primer-binding regions, which did not undergo PCR amplification, exhibited a similar mutation rate (approximately 5.0×10^–4^), implying that the actual mutation rate obtained using the high-fidelity polymerase can be lower.

We further analyzed the patterns of nucleotide substitutions observed in the libraries. With Taq polymerase, transitions (A↔G or C↔T) accounted for 61–72% of all substitutions, and this bias was consistently observed across all Mn^2+^ concentrations tested (Figure 4—figure supplement 1C). This pattern is consistent with previously reported mutation biases associated with this polymerase (LinGoerke et al., 1997). Visualization of the positional distribution of mutation rates further confirmed that error-prone PCR introduced mutations throughout the entire gene sequence (Figure 4—figure supplement 1D).

### Translation of random mutation libraries using near-SGC

**Figure 4.**
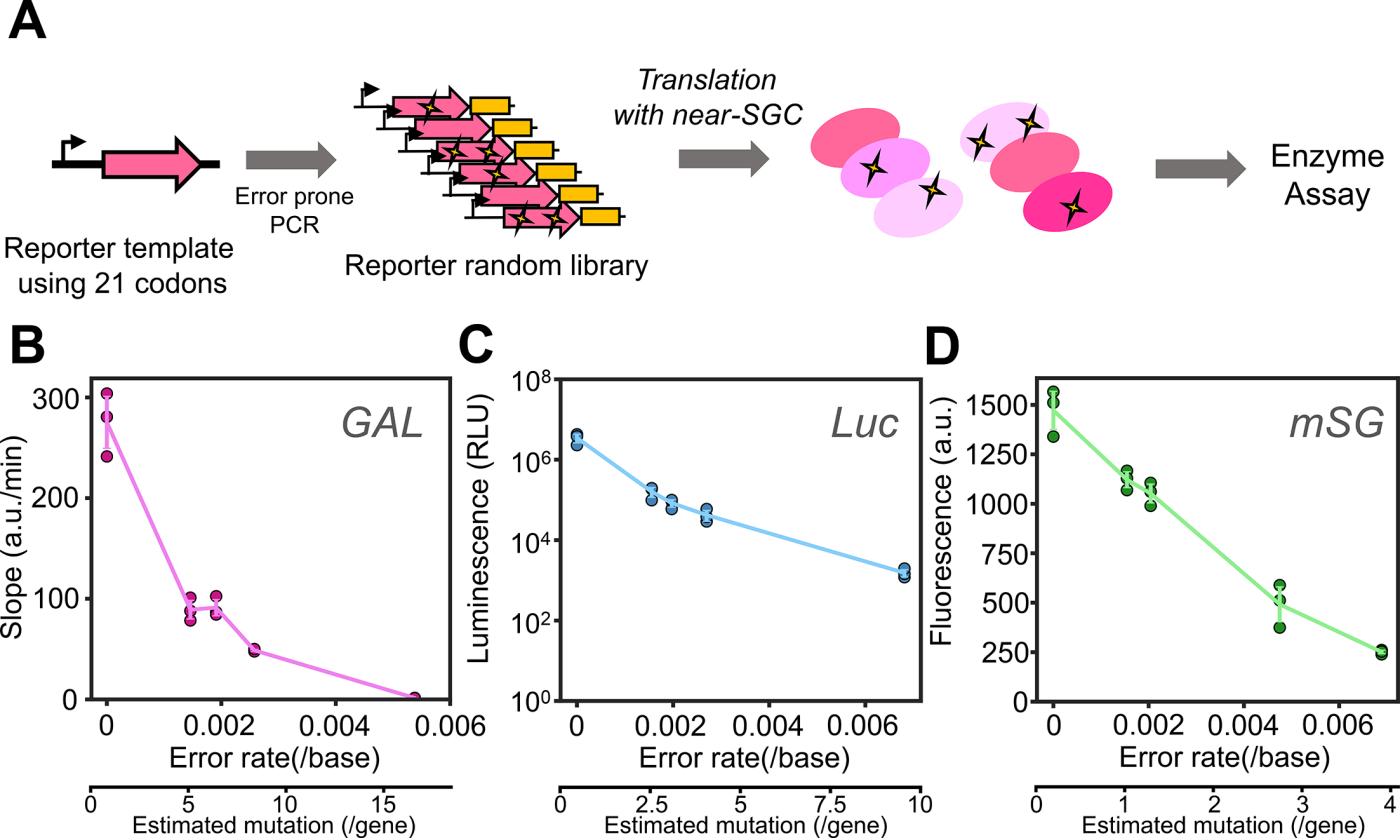
Translation of random mutagenesis libraries with near-SGC. (**A**) Schematic overview of the protein activity assay using a random mutation library. Reporter genes composed of the 21 codons were subjected to random mutagenesis by error-prone PCR at different Mn^2+^ concentrations to generate DNA libraries, as shown in Figure 4—figure supplement 1. These libraries (5 nM) were translated using near-SGC, consisting of a 32-tRNA mixture (tRNA^IPEN^, tRNA^Val^_CAC_, and tRNA^Arg^_CCU_ at 100 ng/µL; all other tRNAs at 12 ng/µL) in tfPURE, including T7 RNA polymerase (1.7 U/µL) at 30 °C for 16 h, and each protein activity was measured. (**B, C, D**) Dependence of β-galactosidase (GAL) (**B**), firefly luciferase (Luc) (**C**), and mStayGold (mSG) (**D**) activity on mutation rate. Note that the vertical axis of panel C (Luc) is on a log scale. Each dot represents the results of three technical replicates, and error bars represent SDs. The lower x-axis indicates the estimated number of mutations per gene, calculated by multiplying the mutation rate per base by the coding sequence length of each reporter gene. Spearman’s rank correlation coefficients were ρ = −0.90 for GAL, ρ = −1.00 for Luc, and ρ = −1.00 for mSG.

To examine the relationship between mutation rates and translated protein function, we performed translation reactions using the near-SGC (RV), containing higher concentrations of two tRNAs (tRNA^Val^_CAC_ and tRNA^Arg^_CCU_) as shown in Figure 1D, and DNA libraries with different mutation rates (Figure 4A). When using the GAL library, we observed a decrease in mean protein activity as the mutation rate increased (Figure 4B), indicating that a sufficient number of mutations were introduced to evaluate the mutational effect on protein function. Similar trends were observed for Luc and mSG (Figure 4C and D), although the magnitude of the decrease varied among proteins. For example, protein activities decreased to 33%, 2.4%, and 79% for GAL, Luc, and mSG, respectively, at a mutation rate of approximately 0.002 per base, indicating that sensitivity to mutations differs substantially among proteins, consistent with a previous study (Lind et al., 2017).

**Figure 4—figure supplement 1.**
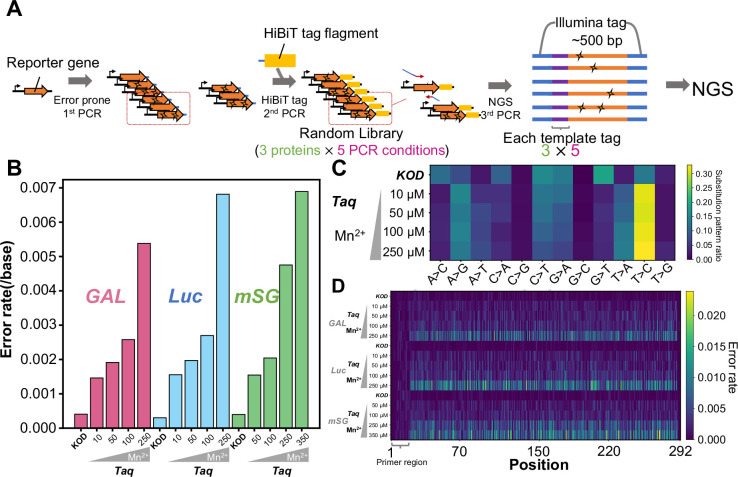
Construction of random libraries by error-prone PCR and analysis of mutation patterns. (**A**) Schematic of the method for preparing DNA libraries. Reporter gene templates composed of the 21 codons (Figure 1B) were amplified by a first PCR using either a high-fidelity polymerase (KOD Plus Neo) or Taq DNA polymerase under error-prone conditions in the presence of Mn^2+^. A second PCR was then performed using the high-fidelity polymerase to append a C-terminal HiBiT tag for protein quantification; these products were used for subsequent translation assays under each genetic code. For sequencing, a third PCR was performed using the high-fidelity polymerase to add Illumina adapters and unique barcode sequences identifying each reporter gene and PCR condition. (**B**) Mean per-base error rates under each 1st PCR condition. The mutation probability was calculated for each position of the template, and the simple average across all positions was used as the mean per-base error rate (see Methods). (**C**) Mutation spectra under each 1st PCR condition. The fractions of each substitution type were computed across all observed substitutions (e.g. ‘A>C’ denotes substitution from A to C). (**D**) Position-wise distribution of error rate. The mutation probability at each nucleotide position is shown.

**Figure 4—figure supplement 2.**
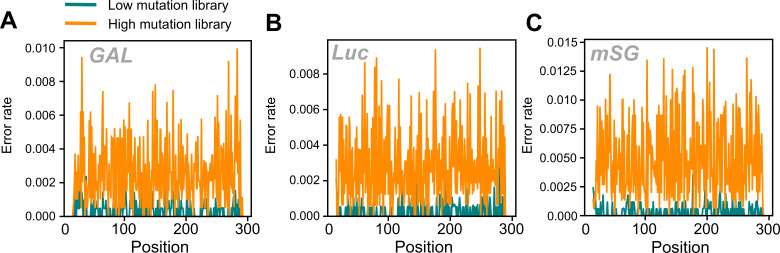
Position-wise distribution of non-reference rates in random mutagenesis libraries. For each reporter gene, the non-reference rate at each position was calculated from amplicon sequencing data and plotted along the analyzed region. Low- and high-mutation libraries are shown separately. These profiles show the positional distribution of mutations introduced by error-prone PCR.

### Translation of random mutation libraries using 10 non-SGCs

**Figure 5.**
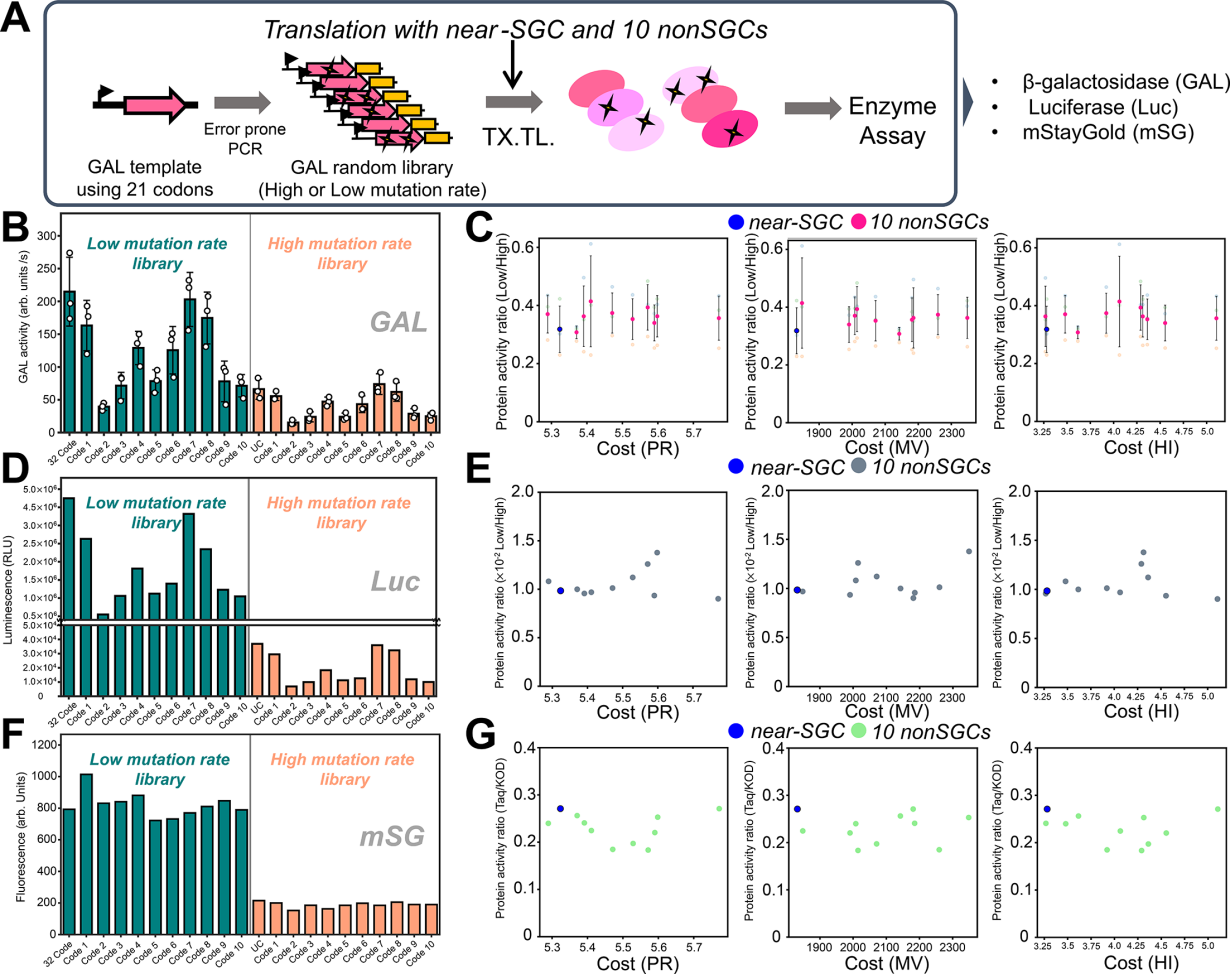
Translation of mutagenized DNA libraries with non-standard genetic codes (non-SGCs). (**A**) Schematic of the experiment for comparing protein activities translated with different genetic codes. Random libraries prepared at low and high mutation rates were translated using either the 10 non-SGCs or the near-SGC (RV). Translation conditions were identical to those described in Figure 4. (**B, D, F**) Protein activities of products translated with each genetic code using low- and high-mutation DNA libraries. Activities are shown for β-galactosidase (GAL; B, mutation rate = 2.6 × 10^–3^ per base), firefly luciferase (Luc; D, mutation rate = 2.7 × 10^–3^ per base), and mStayGold (mSG; F, mutation rate = 4.8 × 10^–3^ per base). Quantification of protein synthesis levels for GAL is shown in Figure 5—figure supplement 1. (**C, E, G**) Ratios of protein activity of high-mutation libraries to those of low-mutation libraries, plotted against the corresponding theoretical mutational costs. Data are shown for GAL (**C**), Luc (**E**), and mSG (**G**). Mean values of three technical replicates are shown with SDs for GAL. For GAL activity in (**B**), two-way ANOVA was performed using genetic code and mutation level as factors. Significant main effects of genetic code and mutation level were detected (both *P*<0.0001), whereas their interaction was not significant. For (**C**), (**E**), and (**G**), Spearman’s rank correlation analysis was performed between each mutational cost metric and the high-/low-mutation activity ratio. Statistical details are summarized in Appendix 1—table 10.

We next evaluated the effects of random mutations on protein activity using 10 non-SGCs constructed above. For this experiment, two random mutation libraries were used: a low-mutation library prepared using the high-fidelity polymerase and a high-mutation library prepared using Taq DNA polymerase at a Mn^2+^ concentration that yields mutation rates of 0.002–0.005 per base (0.0026 for GAL, 0.0027 for Luc, and 0.0048 for mSG, corresponding to approximately 8.0, 4.5, and 3.3 mutations per gene). We also plotted position-wise non-reference rates along the analyzed regions of each reporter gene, confirming that mutations were broadly distributed across the amplicons (Figure 4—figure supplement 2). These libraries were transcribed and translated using 10 non-SGCs selected above, as well as the near-SGC (RV), and the resulting protein activities were measured (Figure 5A).

**Figure 5—figure supplement 1.**
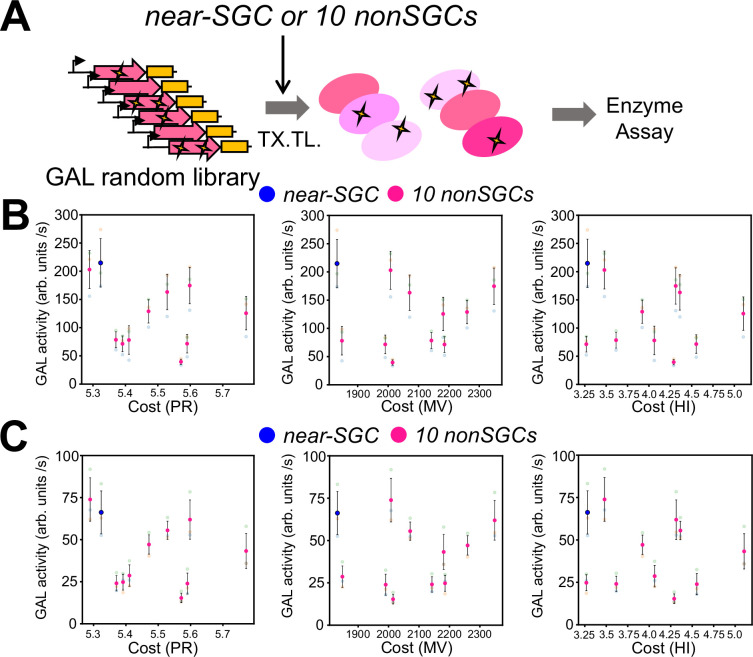
Quantification of translated GAL protein concentrations across different genetic codes. GAL protein synthesized using each low- or high-mutation library under each genetic code was quantified by the HiBiT assay. A HiBiT tag was fused to the C-terminus of the GAL gene. After translation, the HiBiT tag formed an active NanoLuc luciferase upon addition of LgBiT, and the resulting luminescence was measured. A standard curve generated using known concentrations of a HiBiT control protein was measured in parallel and used to quantify the amount of synthesized GAL protein.

**Figure 5—figure supplement 2.**
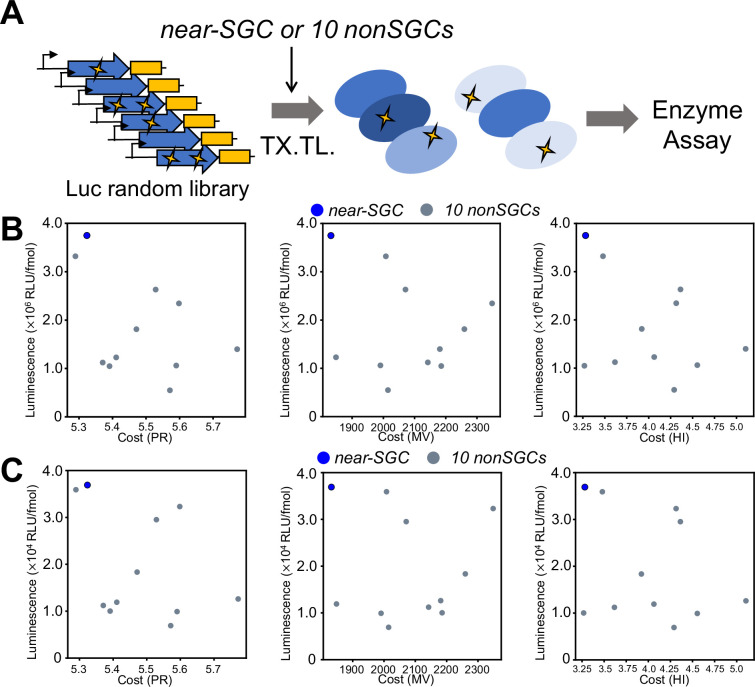
Translation of GAL random library with non-standard genetic codes (non-SGCs). (**A**) Schematic of the experimental procedure. Random DNA libraries prepared at low and high mutation rates were translated using 10 non-SGCs or near-SGC with the GAL random DNA library (5 nM). After incubation at 30 °C for 16 h, GAL activity was quantified. (**B, C**) Distribution of GAL protein activity plotted against theoretical mutational cost for the low-mutation library (**B**) and the high-mutation library (**C**).

**Figure 5—figure supplement 3.**
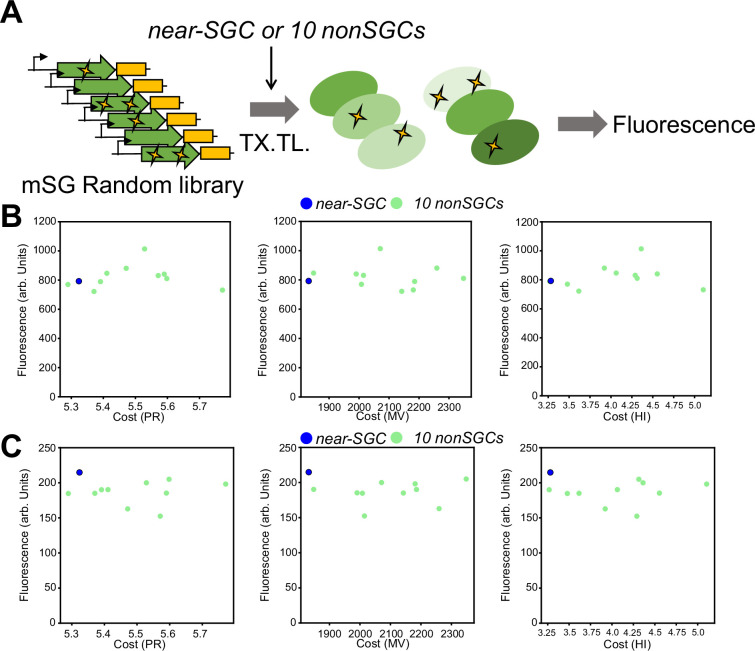
Translation of Luc random library with non-standard genetic codes (non-SGCs). (**A**) Schematic of the experimental procedure. Random DNA libraries prepared at low and high mutation rates were translated using 10 non-SGCs or near-SGC with the Luc random DNA library (5 nM). After incubation at 30 °C for 16 h, luciferase activity was quantified. (**B, C**) Distribution of Luc protein activity plotted against theoretical mutational cost for the low-mutation library (**B**) and high-mutation library (**C**).

**Figure 5—figure supplement 4.**
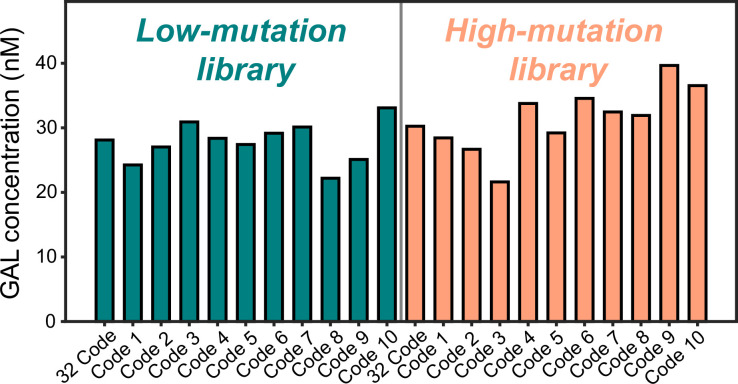
Translation of mSG random library with non-standard genetic codes (non-SGCs). (**A**) Schematic of the experimental procedure. Random DNA libraries prepared at low and high mutation rates were translated using 10 non-SGCs or near-SGC with the mSG random DNA library (5 nM). During incubation at 30 °C for 16 h, mSG fluorescence was quantified. (**B, C**) Distribution of mSG protein activity plotted against theoretical mutational cost for the low-mutation library (**B**) and high-mutation library (**C**).

**Figure 5—figure supplement 5.**
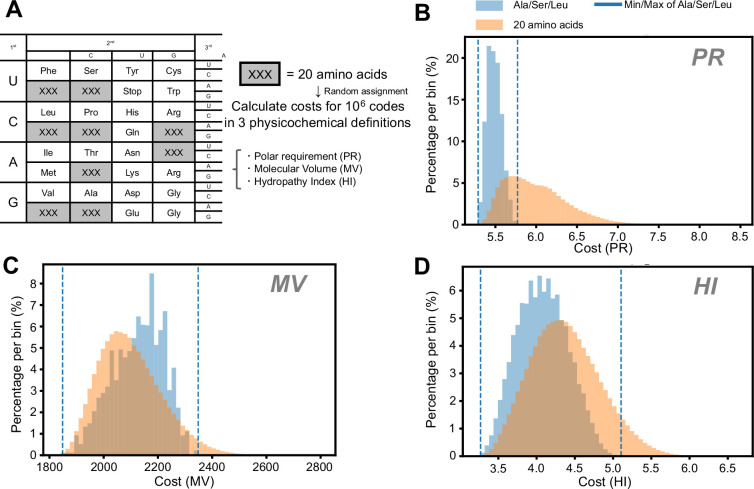
Distributions of the mutational costs when assigning all 20 amino acids to the vacant codons (orange). (**A**) Calculation method of mutational costs for each genetic code based on three physicochemical properties of amino acids. For each genetic code, the average magnitude of change in amino acid physicochemical properties resulting from single-nucleotide substitutions from the 21 codons was calculated, taking mutation weighting into account (see Methods). Three physicochemical metrics were used: polar requirement (PR), molecular volume (MV), and hydropathy index (HI). In this analysis, all 20 amino acids were randomly assigned to each of the nine vacant codon boxes (gray), in contrast to the analysis in Figure 3B, in which only three amino acids (Ala, Leu, and Ser) were assigned. Mutational costs were calculated for 1,000,000 randomly sampled genetic codes. (**B, C, D**) Distributions of mutational costs for each physicochemical property of amino acids when assigning 20 amino acids (orange). The distributions obtained when assigning only three amino acids (Ala, Ser, and Leu), identical to the data shown in Figure 3B, are shown for comparison (blue). Dashed lines indicate the maximum and minimum values of the blue distribution, the cost ranges of the experimentally constructed non-standard genetic codes (non-SGCs) in this study.

**Figure 5—figure supplement 6.**
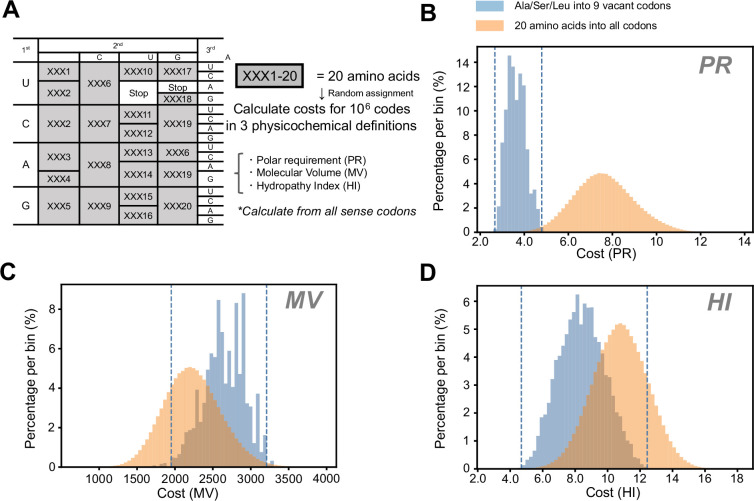
Distributions of the mutational costs when assigning all 20 amino acids to all sense codons (orange). (**A**) Calculation method of mutational costs for each genetic code based on three physicochemical properties of amino acids. For each genetic code, the average magnitude of change in amino acid physicochemical properties resulting from single-nucleotide substitutions from all sense codons was calculated, taking mutation weighting into account (see Methods). Three physicochemical metrics were used: polar requirement (PR), molecular volume (MV), and hydropathy index (HI). In this analysis, all 20 amino acids were randomly assigned to each of the 20 codon boxes (gray) while preserving the degeneracy pattern of the standard genetic code (SGC). Mutational costs were calculated for 1,000,000 randomly sampled genetic codes. (**B, C, D**) Distributions of mutational costs for each physicochemical property of amino acids when assigning all 20 amino acids into all sense codons (orange). The distributions obtained when assigning only three amino acids (Ala, Ser, and Leu), identical to the data shown in Figure 3B, are shown for comparison (blue). Dashed lines indicate the maximum and minimum values of the blue distribution, the cost ranges of the experimentally constructed non-standard genetic codes (non-SGCs) in this study.

**Figure 5—figure supplement 7.**
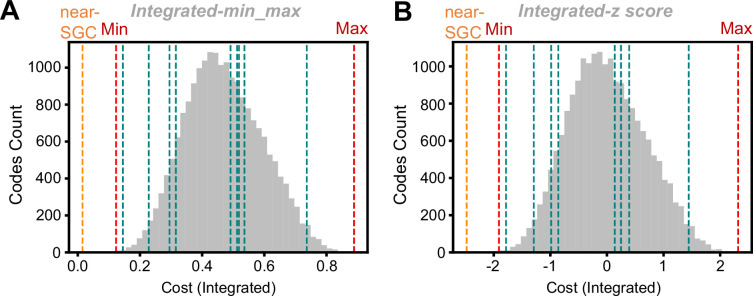
Integrated mutational cost analysis combining PR, MV, and HI. (**A**) Distribution of integrated min–max costs among 19,683 candidate non-standard genetic codes (non-SGCs). For each cost metric, Cost_PR_, Cost_MV_, and Cost_HI_ were min–max normalized across the candidate non-SGCs and averaged with equal weights. The orange dashed line indicates the near-SGC reference, and the red dashed line indicates the candidate non-SGC with the lowest and highest integrated cost. The green dashed lines indicate the cost values of 10 genetic codes selected for experimental construction. (**B**) Distribution of integrated z-score costs among candidate non-SGCs. For each metric, costs were z-score normalized using the mean and SD of the candidate non-SGC distribution and averaged with equal weights.

We first examined GAL activities. For the low-mutation library, mean protein activity varied by up to 5.4-fold among different genetic codes (Figure 5B left, green bars). For the high-mutation library, GAL activity decreased overall, while the relative differences in activity among genetic codes observed in the low-mutation library were broadly retained (Figure 5B right, orange bars). Quantification of protein synthesis levels using the HiBiT assay revealed similar protein synthesis levels both among the different genetic codes and between the low- and high-mutation rates (Figure 5—figure supplement 1), indicating that the variation in protein activity among genetic codes and between low- and high-mutation rates was not attributable to differences in translational levels. The variation in protein activity among genetic codes with the low-mutation library was possibly caused by differences in translational error frequencies among genetic codes. Such differences are not surprising because different tRNA sets were used for each genetic code, and each tRNA is expected to have distinct translational accuracies. However, the purpose of this study is to evaluate how the effect of mutations on protein function varies depending on the genetic code; this effect can be extracted even in the presence of different translational error rates by normalizing the protein activity of the mutated library by that of the less-mutated library.

To estimate the average activity reduction associated with increased mutational burden under each genetic code, we calculated the ratio of activity obtained from the high-mutation library to that from the corresponding low-mutation library and plotted this ratio against each of the three mutational costs (Figure 5C). Data before this normalization are shown in Figure 5—figure supplements 2–4. According to the error minimization theory, genetic codes with higher mutational costs tend to reduce the protein activity more upon mutagenesis; thus, a negative correlation between mutational cost and protein activity would be expected. Contrary to this expectation, the effect of mutations on GAL activity remained nearly constant across the ranges of all three mutational costs examined (Figure 5C).

We performed the same experiment for Luc (Figure 5D) and mSG (Figure 5F), and the results were similar to those obtained by GAL. For both proteins, activity with the low-mutation library varied among genetic codes and decreased with the high-mutation library, although the variation among genetic codes was smaller for mSG. The activity ratios of the high- to low-mutation libraries showed no clear dependence on any of the three mutational costs (Figure 5E and G). Taken together, these results indicate that the mutational robustness of individual reporter protein function did not substantially differ among the genetic codes that exhibit the mutational cost ranging from 5.29 to 5.77 for PR, 1848 to 2348 for MV, and 3.27–5.10 for HI.

## Discussion

In this study, we constructed 10 non-SGCs by assigning Ala, Ser, and Leu to vacant codons in MGC. These non-SGCs represent mutational costs ranging from 5.29 to 5.77 for Cost_PR_, 1848 to 2348 for Cost_MV_, and 3.27–5.10 for Cost_HI_, most of which are higher than those of SGC. The error minimization theory predicts that the deleterious effect of mutations on protein function becomes more severe when using genetic codes with higher mutational cost (Freeland et al., 2003; Freeland and Hurst, 1998; Haig and Hurst, 1991; Massey, 2008). In contrast to this prediction, translation experiments using the non-SGCs and mutagenized libraries revealed that the effects of mutations on protein functions were similar across all genetic codes and reporter genes examined here, suggesting that the mutational robustness of protein activity remained largely unchanged within at least the ranges of mutational cost tested in this study. It should be noted that this conclusion is limited to the activity of individual reporter proteins translated in a reconstituted in vitro system. Therefore, whether similar trends would be observed at the level of cellular fitness or long-term evolution remains an open question. Moreover, our results do not exclude other possible roles of SGC organization. The SGC may have been shaped by multiple factors, including robustness to translational errors, historical constraints during genetic code expansion, biosynthetic or coevolutionary relationships among amino acids, stereochemical interactions, and effects on protein evolvability (Katoh and Suga, 2023; Koonin and Novozhilov, 2017; Koonin and Novozhilov, 2009; Novozhilov et al., 2007; Wong, 2005).

Although protein amounts quantified by the HiBiT tag were comparable among genetic codes, GAL activities differed substantially. This indicates that the activity differences among genetic codes were not primarily attributable to differences in the amount of C-terminally completed translation products. The HiBiT assay does not provide information on the fraction of catalytically active protein, including sequence fidelity or folding state, and therefore cannot distinguish among these possibilities. Detailed characterization of translated products by mass spectrometry would provide further mechanistic insight into how individual non-SGCs affect protein quality. However, the primary objective of the present study was to compare mutation-dependent activity loss across genetic codes. Therefore, we evaluated this effect by normalizing the activity of the high-mutation library to that of the corresponding low-mutation library within each genetic code.

A further limitation of this study is that the reporter activities were measured at the level of pooled random mutation libraries. Therefore, the high-/low-mutation activity ratio used in this study should be interpreted as the relative reduction in average activity caused by increasing the mutational burden in a heterogeneous mutation pool, rather than as the effect of identical variants before and after additional mutations. This library-averaged approach was chosen because the mutational costs considered here are also defined as average expected physicochemical effects over many possible single-nucleotide substitutions. In addition, because the non-SGCs constructed in this study were generated by reassigning only Ala, Ser, and Leu, the detectable effects may depend on how frequently mutations involving these amino acids occur in each reporter gene and whether the affected positions are functionally important. If genetic code-dependent effects are restricted to a small subset of deleterious variants, such effects may be masked in pooled activity measurements. Future studies using defined variants or high-throughput genotype–phenotype mapping assays will be required to determine the mutation-specific and position-specific mechanisms underlying genetic code-dependent effects on protein function (Rozhoňová et al., 2024).

It should be noted that the range of mutational costs experimentally tested in this study is limited because we could only reassign three amino acids (Ala, Ser, and Leu) to the vacant codons in MGC. If all 20 amino acids could be reassigned to the nine vacant codons, the possible range of mutational costs would be 5.29–8.49 for Cost_PR_, 1826 – 2805 for Cost_MV_, and 3.21–6.65 for Cost_HI_ (Figure 5—figure supplement 5). The genetic codes constructed in this study cover approximately 15.1%, 51.1%, and 53.3% of these possible cost ranges, for PR, MV, and HI, respectively. Furthermore, if we could assign any amino acids to any codon boxes with a degenerate pattern similar to that of SGC, as assumed in previous theoretical studies (Freeland and Hurst, 1998; Haig and Hurst, 1991), the cost ranges were 2.67–4.79 for Cost_PR_, 1952 – 3209 for Cost_MV_, and 4.69–12.44 for Cost_HI_ for one million randomly sampled genetic codes. The non-SGCs constructed in this study covered 18.4%, 37.6%, and 50.8% of these possible cost ranges for PR, MV, and HI, respectively (Figure 5—figure supplement 6). We consider that the cost ranges examined in this study represent substantial fractions, especially for MV and HI. Although the near-SGC did not necessarily exhibit the lowest cost for each individual physicochemical metric, this does not mean that it is unfavorable in the multidimensional cost space. Because the SGC may reflect a balance among multiple physicochemical constraints rather than optimization of a single property, we also calculated integrated cost indices by combining Cost_PR_, Cost_MV_, and Cost_HI_ after min–max normalization or z-score normalization. In both integrated indices, the near-SGC showed the lowest overall cost when compared with all 19,683 candidate non-SGCs (Figure 5—figure supplement 7), indicating that no candidate non-SGC exhibited a lower combined cost than the near-SGC when the three physicochemical properties were considered jointly. However, we also think that the finding of this study – mutational robustness does not detectably depend on the genetic codes – might change if non-SGCs with broader cost ranges could be constructed.

This limitation in the achievable mutational cost ranges in this study arises from the fact that only three amino acids (Ala, Ser, and Leu) can be reassigned to different codons. The other amino acids cannot be reassigned to different codons because their corresponding tRNAs require the cognate anticodon sequence for recognition by the corresponding aaRS. To construct genetic codes with a broader range of mutational costs, it will be necessary to identify new aaRSs that do not recognize anticodon sequence but still catalyze aminoacylation for the other 17 amino acids. To date, such aaRSs have not been clearly reported, but some aaRSs, such as a mitochondrial aaRS with altered anticodon recognition (Su et al., 2011) and aaRS mutants that shuffle the anticodon recognition domain (Brevet et al., 2003), may be suitable for this purpose. Although numerous engineered orthogonal aaRS–tRNA pairs have been developed to incorporate non-canonical amino acids into proteins, these systems are typically designed to target stop or limited sense codons (Costello et al., 2024; Robertson et al., 2021; Shandell et al., 2021; Zeng et al., 2014). Alternatively, ribozyme-based aminoacylation systems such as flexizymes (Goto and Suga, 2021; Jones et al., 2025; Murakami et al., 2006; Terasaka et al., 2014) offer a more flexible route to assigning diverse amino acids to vacant codons, although protein synthesis using ribozyme-based aminoacylation is still a challenge (Chen et al., 2021).

The results of this study suggest that altering the arrangement of the genetic code does not necessarily lead to a drastic loss of functional robustness against mutations. This finding indicates that genetic codes can be redesigned for specific purposes without severe functional penalties, at least in vitro. This insight paves the way for utilizing engineered genetic codes (Huang et al., 2025; Pines et al., 2017; Zürcher et al., 2022) for broader applications in protein engineering and in vitro synthetic biology, such as genetic code expansion using non-canonical amino acids (Iwane et al., 2016; Jones et al., 2025; Katoh and Suga, 2022; McFeely et al., 2023; Passioura et al., 2018), and the design of artificial translation systems orthogonal to natural translation systems for biocontainment (Calles et al., 2019; Fujino et al., 2024; Fujino et al., 2020; Nyerges et al., 2023; Robertson et al., 2021). Accelerated protein evolution may also be achievable using non-SGCs (Pines et al., 2017; Zürcher et al., 2022) because the structure of the genetic code determines the amino acids accessible through mutation. While some theoretical studies suggest that alternative genetic codes could enhance evolvability (Pines et al., 2017), others have argued that the SGC itself possesses advantageous evolvability features (Firnberg and Ostermeier, 2013; Rozhoňová et al., 2024). The knowledge obtained in this study enhances the usefulness of genetic code engineering in vitro for broader applications.

## Methods

**Key resources table:** 

Reagent type (species) or resource	Designation	Source or reference	Identifiers	Additional information
Recombinant DNA reagent	NanoLuc reporter gene (21 codons)	Twist Bioscience (fragment synthesis); this paper	n/a	Designed to use only 21 codons compatible with MGC; sequences listed in Appendix 1—table 1
Recombinant DNA reagent	NanoLuc reporter gene (32 codons)	Twist Bioscience (fragment synthesis); this paper	n/a	Designed to use 32 codons compatible with near-SGC; sequences listed in Appendix 1—table 1
Recombinant DNA reagent	NanoLuc reporter gene (46 codons)	Twist Bioscience (fragment synthesis); this paper	n/a	Designed to use 46 codons compatible with SGC; sequences listed in Appendix 1—table 1
Recombinant DNA reagent	NanoLuc reporter genes for codon reassignment experiments (21+1 variants; 25 constructs total)	Twist Bioscience (fragment synthesis); this paper	n/a	Based on 21-codon NanoLuc; 2 Ala, 3 Ser, or 4 Leu codons replaced with target vacant codons; sequences listed in Appendix 1—table 1
Recombinant DNA reagent	*lacZ* (β-galactosidase; GAL) reporter gene	Twist Bioscience (fragment synthesis); this paper	n/a	Designed using 21-codon set; sequences listed in Appendix 1—table 1
Recombinant DNA reagent	Firefly luciferase (Luc) reporter gene	Twist Bioscience (fragment synthesis); this paper	n/a	Designed using 21-codon set; sequences listed in Appendix 1—table 1
Recombinant DNA reagent	mStayGold (mSG) reporter gene	Twist Bioscience (fragment synthesis); this paper	n/a	Designed using 21-codon set; sequences listed in Appendix 1—table 1
Recombinant DNA reagent	Plasmid templates for tRNA transcription (21-tRNA set for MGC)	Miyachi et al., 2025; Miyachi et al., 2022	PMID:40858540; PMID:35848947	Used as templates for site-directed mutagenesis (inverse PCR) to generate anticodon variants
Recombinant DNA reagent	Plasmid templates for tRNA transcription (11 additional tRNAs for near-SGC; 14 for SGC; anticodon variants for Ala, Ser, Leu)	This paper	n/a	Anticodon regions replaced by site-directed mutagenesis (inverse PCR); primer sequences in Appendix 1—table 2; tRNA sequences in Appendix 1—table 3
Sequence-based reagent	21-tRNA set for MGC (in vitro-transcribed or chemically synthesized tRNAs)	Miyachi et al., 2025; Miyachi et al., 2022; Agilent (chemical synthesis)	PMID:40858540; PMID:35848947	tRNA sequences in Appendix 1—table 3; chemically synthesized tRNAs: tRNAAsn(GUU), tRNAGln(CUC), tRNAfMet(CAU), tRNAIle(GAU), tRNATrp(CCA), tRNAPro(GGG); remainder transcribed in vitro
Sequence-based reagent	11 additional tRNAs for near-SGC: tRNALeu(CAA), tRNASer(CGA), tRNALeu(CAG), tRNAPro(CGG), tRNAArg(CCG), tRNASer(GCU), tRNAThr(CGU), tRNAArg(CCU), tRNAVal(CAC), tRNAAla(CGC), tRNAGly(CCC)	This paper	n/a	Anticodon region replaced from 21-tRNA set templates; tRNA sequences in Appendix 1—table 3; (tRNAVal(CAC) and tRNAArg(CCU) used at 100 ng/µL; others at 12 ng/µL)
Sequence-based reagent	14 additional tRNAs with UNN anticodons for SGC (NNA codon decoding)	This paper	n/a	Anticodon region replaced from 21-tRNA set templates; tRNA sequences in Appendix 1—table 3
Sequence-based reagent	Anticodon variant tRNAs for Ala, Ser, and Leu (reassignment to 9 vacant codons: UUG, UCG, CUG, CCG, CGG, ACG, AGC, GUG, GCG)	This paper	n/a	Used to construct 10 non-SGCs; tRNA concentrations: tRNAAla(CAA) 40 ng/µL, tRNAAla(CGA) 60 ng/µL, tRNAAla(CGU) 60 ng/µL, tRNALeu(CGU) 80 ng/µL, tRNALeu(CGA) 80 ng/µL, tRNALeu(GCU) 80 ng/µL; others 12 ng/µL
Sequence-based reagent	PCR primers (for DNA template preparation, tRNA transcription template preparation, library preparation, and Illumina sequencing)	This paper	n/a	Sequences listed in Appendix 1—table 2; primers include T7 promoter-containing forward primers and 2'-O-methyl-modified reverse primers for run-off tRNA transcription
Peptide, recombinant protein	T7 RNA polymerase	Takara Bio	n/a	Used for in vitro tRNA transcription (1 U/µL) and cell-free translation (0.42–1.7 U/µL)
Peptide, recombinant protein	KOD Plus Neo DNA polymerase	Toyobo	n/a	Used for low-mutation PCR, tRNA template preparation, and library preparation
Peptide, recombinant protein	Taq DNA polymerase	New England Biolabs	n/a	Used for error-prone PCR; supplemented with MgCl2 (3.0 mM final) and MnCl2 (10–350 µM)
Peptide, recombinant protein	DpnI	New England Biolabs	n/a	Used to remove template plasmid after PCR amplification of tRNA transcription templates; incubated at 37 °C for 2 h
Peptide, recombinant protein	Inorganic pyrophosphatase	New England Biolabs	n/a	Used in in vitro tRNA transcription reactions (2 U/µL)
Peptide, recombinant protein	RNasin (ribonuclease inhibitor)	Promega	n/a	Used in in vitro tRNA transcription reactions (0.8 U/µL)
Peptide, recombinant protein	PURE system components (ribosomes, translation factors, aminoacyl-tRNA synthetases, energy-regeneration components)	Miyachi et al., 2025; Miyachi et al., 2022	PMID:40858540; PMID:35848947	Laboratory-made tRNA-free PURE system (tfPURE); individual factors expressed with His-tags and purified by affinity +gel-filtration chromatography; EF-Tu subjected to two rounds of affinity purification; ribosomes purified by butyl column +sucrose cushion +ultrafiltration; complete composition in Appendix 1—table 6
Commercial assay or kit	Luciferase Assay Reagent	Promega	n/a	Used for firefly luciferase activity measurement (1 µL translation reaction +30 µL reagent)
Commercial assay or kit	NanoLuc Assay Reagent	Promega	n/a	Used for NanoLuc luciferase activity measurement (1 µL translation reaction +50 µL reagent)
Commercial assay or kit	HiBiT tag / HiBiT assay system	Promega	n/a	Used for quantification of C-terminally tagged translation products; HiBiT tag attached by overlap extension PCR (2nd PCR)
Commercial assay or kit	FastGene Gel/PCR Extraction Kit	Nippon Genetics	n/a	Used for purification of PCR products and DNA fragments
Commercial assay or kit	PureLink RNA Mini Kit	Invitrogen	n/a	Used for purification of in vitro-transcribed tRNAs
Chemical compound, drug	MnCl2	Other	n/a	Used in error-prone PCR at 10, 50, 100, 250, or 350 µM to modulate mutation rates; promotes Taq polymerase infidelity
Chemical compound, drug	TokyoGreen-βGal	Sekisui Medical	n/a	Fluorescent substrate for β-galactosidase (GAL) activity assay (5 µM final); GAL activity determined from slope of fluorescence time course
Chemical compound, drug	NTPs (ATP, GTP, CTP, UTP); GMP	Other	n/a	Used in in vitro tRNA transcription (2 mM each NTP; 3 mM GMP)
Chemical compound, drug	Spermidine	Other	n/a	Used in in vitro tRNA transcription buffer (2 mM)
Chemical compound, drug	DTT	Other	n/a	Used in tRNA transcription (5 mM) and GAL assay buffer (5 mM)
Software, algorithm	BWA-MEM	Other	RRID:SCR_010910	Used for paired-end read alignment to amplicon reference sequences
Software, algorithm	SAMtools	Other	RRID:SCR_002105	Used for BAM processing (sorting, indexing) and position-wise base extraction (mpileup; MAPQ ≥20, Phred Q≥30)
Software, algorithm	cutadapt	Other	RRID:SCR_011841	Used for demultiplexing of sequencing reads by barcode sequence (perfect full-length match required)
Software, algorithm	Mutational cost calculation script	This paper	n/a	Custom script to calculate CostPR, CostMV, and CostHI for 19,683 candidate genetic codes; based on formulations in Freeland and Hurst, 1998 and Haig and Hurst, 1991; substitution weights in Appendix 1—table 4; amino acid physicochemical values in Appendix 1—table 5. The code is included in Source Code file (Source code 1).
Other	GloMax luminometer	Promega	RRID:SCR_018613	Used to measure firefly luciferase and NanoLuc luminescence
Other	Mx3005P real-time PCR system	Agilent Technologies	n/a	Used for continuous fluorescence monitoring of mStayGold (mSG) during translation (up to 16 h); also used for GAL fluorescence assay (FAM detection settings, 1 min intervals for 60 min)
Other	NanoDrop spectrophotometer	Thermo Fisher Scientific	RRID:SCR_016517	Used for DNA and RNA concentration determination by absorbance at A260
Other	Illumina MiSeq	Illumina	RRID:SCR_016379	Used for paired-end sequencing of random mutation libraries (~500 bp amplicons)

### DNA preparation

All DNA templates encoding reporter proteins were obtained through a fragment synthesis service provided by Twist Bioscience. The NanoLuc reporter genes used for translation under MGC, near-SGC, or SGC were designed to utilize 21, 32, or 46 codons (Figure 1B). For validation of translation by each anticodon variant tRNA in Figure 2, NanoLuc reporter genes were designed based on the 21 codon set, in which two alanine, three serine, or four leucine codons were replaced by target codons, producing a total of 25 distinct NanoLuc templates. For assays involving non-SGCs, DNA sequences encoding β-galactosidase (GAL), firefly luciferase (Luc), and mStayGold (mSG) were designed using the 21 codon set. The sequences of all DNA templates used in this study are provided in Supplementary Appendix 1—table 1.

To prepare templates for translation reactions, PCR amplification was performed using primers 1 and 2, which were common to all constructs. For PCR under low-mutation conditions, KOD Plus Neo DNA polymerase (Toyobo) was used. Error-prone PCR conditions are described in a separate section below. To prepare templates with a C-terminal HiBiT tag for protein quantification (Figure 4—figure supplement 1, 2nd PCR), a HiBiT tag fragment was first amplified using primers 3 and 4. This fragment was then fused to the coding region of each reporter gene by overlap extension PCR; PCR products encoding GAL, Luc, or mSG were amplified using primers 1 and 5 (GAL), 6 (Luc), or 7 (mSG), respectively, using either KOD Plus Neo for the low-mutation library or Taq DNA polymerase for the higher-mutation libraries, and subsequently fused with the HiBiT fragment using primers 1 and 8. The resulting DNA fragments were purified using the FastGene Gel/PCR Extraction Kit (Nippon Genetics), and DNA concentrations were determined from A260 measurements using a NanoDrop spectrophotometer (Thermo Fisher Scientific). The sequences of all primers used in this study are listed in Supplementary Appendix 1—table 2.

### Error-prone PCR

To introduce random mutations into protein-coding DNA, error-prone PCR was performed as described schematically in Figure 4—figure supplement 1 (1st PCR). PCR reactions were carried out using Taq DNA polymerase (New England Biolabs) in the manufacturer-recommended Standard Taq Reaction Buffer supplemented with additional MgCl_2_ and MnCl_2_. The final concentration of MgCl_2_ was adjusted to 3.0 mM. To modulate the mutation rates, MnCl_2_ was added at final concentrations of 10, 50, 100, 250, or 350 µM. PCR amplification was performed for 30 cycles, and the resulting products were used as random DNA libraries for subsequent experiments.

### tRNA preparation

Among the 21 tRNAs used in MGC, those corresponding to our previous work (Miyachi et al., 2025; Miyachi et al., 2022) were used without modification, whereas the remaining 11 tRNAs for near-SGC and 14 for SGC were newly prepared by replacing their anticodon sequences. For these tRNAs, plasmid templates encoding the original sequences of the corresponding 21-tRNAs set were used as templates, and only the anticodon region was replaced by site-directed mutagenesis using inverse PCR. Anticodon variants of tRNA^Ala^, tRNA^Ser^, and tRNA^Leu^ were prepared in the same manner, with substitutions restricted to the anticodon region. The primer sets used for inverse PCR and the sequences of all tRNAs are listed in Appendix 1—tables 2 and 3.

Plasmids constructed by inverse PCR were used as templates for tRNA transcription. To prepare transcription templates, PCR amplification was performed using a forward primer containing a T7 promoter sequence and a reverse primer designed for run-off transcription carrying a 2′-O-methyl modification (Appendix 1—tables 2 and 3). PCR reactions were carried out using KOD Plus Neo DNA polymerase (Toyobo) according to the manufacturer’s instructions. Following amplification, PCR products were treated with DpnI at 37 °C for 2 hr to remove template plasmids. The resulting DNA fragments were purified, and DNA concentrations were determined as described above.

Of the 46 tRNAs used in this study, the majority were prepared by in vitro transcription using T7 RNA polymerase. To enable efficient transcription, tRNAs whose native sequences do not begin with guanosine at the 5′-end were redesigned as 5′-G variants unless otherwise noted. Consequently, most tRNAs were synthesized by in vitro transcription regardless of their native 5′-terminal nucleotide. Exceptions were tRNA^Asn^_GUU_, tRNA^Gln^_CUC_, tRNA^fMet^_CAU_, tRNA^Ile^_GAU_, tRNA^Trp^_CCA_, and tRNA^Pro^_GGG_, which were obtained by chemical synthesis (Agilent), as our previous report (Miyachi et al., 2022).

In vitro transcription reactions were performed in a mixture containing 40 mM Tris–HCl (pH 8.0), 10 mM MgCl_2_, 2 mM spermidine, 5 mM DTT, 1 U/µL T7 RNA polymerase (Takara Bio), 2 mM each NTP (ATP, GTP, CTP, and UTP), 3 mM GMP, 5 ng/µL template DNA, 2 U/µL inorganic pyrophosphatase (New England Biolabs), and 0.8 U/µL RNasin (Promega). Reactions were incubated at 37 °C for 12 hr, and RNA products were purified using the PureLink RNA Mini Kit (Invitrogen). Purified tRNAs were dissolved in water and stored at –80 °C until use. RNA concentrations were determined by absorbance at 260 nm.

### Calculation of mutational cost

The mutational costs for each genetic code, $Cost\left (C\right)$, were calculated based on previously proposed formulations of genetic code optimality (Freeland and Hurst, 1998; Haig and Hurst, 1991). In this calculation, the cost reflects the expected change in amino acid property caused by single-nucleotide substitutions from the initial 21-codon sets in this study (Figure 1B). Details of calculations are as follows. We denote each codon in the 21-codon set as $\beta $, and the amino acid encoded by $\beta $ as $AA\left (\beta \right)$. For each $\beta $, we identified the set of codons reachable by a single-nucleotide substitution, denoted as $N\left (\beta \right)$. Each element of this set is represented as a pair $\left (\beta ',\,w_{\beta \rightarrow \beta '}\right)$, where $\beta '$ is a neighboring codon box and $w_{\beta \rightarrow \beta '}$ is a weight reflecting the relative likelihood of the corresponding nucleotide substitution. These weights account for differences in substitution probabilities depending on nucleotide position and whether the substitution is a transition or transversion. The substitution weights were adopted from the previous reports (Freeland and Hurst, 1998; Omachi et al., 2023) and are summarized in Appendix 1—table 4.

For each possible substitution from $\beta $ to $\beta '$, we calculated the physicochemical distance between the corresponding amino acids, $\Delta \left (AA\left (\beta \right),AA\left (\beta '\right)\right)$. This distance was evaluated using three physicochemical properties: PR, MV, and HI. For each property, the distance was defined as the squared difference between the property values of the two amino acids. For example, for PR, the distance function is defined as:

(1) $$\displaystyle \Delta \left (\alpha _{1},\alpha _{2}\right)=\left (PR\left (\alpha _{1}\right)-PR\left (\alpha _{2}\right)\right)^{2}$$

The expected cost contribution of each substitution was calculated by multiplying the amino acid distance by the corresponding substitution weight. The overall mutational cost was then obtained by summing these weighted distances over all single-nucleotide substitutions from all codon boxes in the 21-codon set and dividing by the total number of substitutions considered. Formally, the cost of the genetic code $C$ was defined as:

(2) $$\displaystyle  Cost\left (C\right)\colon =\ \frac{\underset{\beta \in B_{21\ codons}}{\sum }\underset{\left (\beta ',\ w_{\beta \rightarrow \beta '}\right)\in N\left (\beta \right)}{\sum }w_{\beta \rightarrow \beta '}\cdot \Delta \left (AA\left (\beta \right),AA\left (\beta '\right)\right)}{\underset{\beta \in B_{21\ codons}}{\sum }|N\left (\beta \right)|}$$

Here, $B_{21\,codons}$ denotes the set of codon boxes fixed in the 21-box code. The denominator represents the total number of possible single-nucleotide substitutions considered, ensuring that the cost corresponds to the average expected physicochemical impact per mutation. Physicochemical property values for each amino acid were taken from the datasets adopted by Haig and Hurst, 1991, and are listed in Appendix 1—table 5.

For integrated cost analysis, Cost_PR_, Cost_MV_, and Cost_HI_ were combined after normalization because the three metrics have different numerical scales. For min–max normalization, each metric $m\in \left \{PR,MV,HI\right \}$ cost $Cost_{m_{norm}}\left (C\right)$ was normalized across the set $G$ of candidate non-SGCs as

(3) $$\displaystyle Cost_{m_{norm}}\left (C\right)=\frac{Cost_{m}\left (C\right)-min_{h\in G}\left (h\right)}{max_{h\in G}\left (h\right)-min_{h\in G}\left (h\right)}$$

The integrated min–max cost was defined as the equal-weight mean of the three normalized costs.

For z-score normalization, each cost was transformed as

(4) $$\displaystyle Z_{m}\left (g\right)=\frac{Cost_{m_{norm}}\left (C\right)-\mu _{m,G}}{\sigma _{m,G}}$$

where $\mu _{m,G}$ and $\sigma _{m,G}$ are the mean and SD across candidate non-SGCs. The integrated z-score cost was defined as the equal-weight mean of the three z-scores. The near-SGC was evaluated using the same normalization parameters calculated from the candidate non-SGC distribution.

### Sequence analysis of random libraries

Random mutagenesis libraries (2nd PCR products; Figure 4—figure supplement 1) were used as templates for amplicon preparation for sequencing (3rd PCR). Using 40 pM of each library as input, an approximately 500 bp region spanning the coding sequence of each reporter gene was amplified by PCR using KOD Plus Neo DNA polymerase (Toyobo). Primers used for this PCR contained both sample-identifying barcodes and Illumina adapter sequences. For GAL, primers 107–111 were used as forward primers with primer 112 as a common reverse primer. For Luc, primers 113–117 were used as forward primers with primer 118 as a common reverse primer. For mSG, primers 119–123 were used as forward primers with primer 124 as a common reverse primer. PCR products were purified using the FastGene Gel/PCR Extraction Kit (Nippon Genetics). DNA concentrations were determined by absorbance at 260 nm and subjected to Illumina MiSeq sequencing.

Paired-end FASTQ files generated by the Illumina platform were used for all analyses. Libraries were prepared from three reporter genes across five error-prone PCR conditions, with each sample corresponding to an amplicon of approximately 500 bp. Sample-identifying barcode sequences were positioned at the 5′ end of read 1 (R1) of the pair-end sequencing, and the R1 reads covering approximately 300 bp from the 5′ side of each amplicon were used for non-reference-rate analysis. Demultiplexing was performed using cutadapt, requiring a perfect full-length match to the barcode sequence; only read pairs in which the R1 barcode matched exactly were retained, and all other read pairs were excluded from downstream analyses. Demultiplexed FASTQ files were generated for each sample. Paired-end reads were aligned to the corresponding amplicon reference sequence (generated for each reporter gene and condition) using BWA-MEM. Alignments were processed with SAMtools to generate sorted and indexed BAM files. Position-wise base information was extracted using SAMtools mpileup, applying filters of mapping quality MAPQ ≥20 (−q 20) and base quality Phred Q≥30 (−Q 30). Insertions and deletions were excluded from all analyses.

For each nucleotide position *i*, the mutation count $n_{i}$ was defined as the total number of bases in the pileup that differed from the reference base, and the total coverage at that position was denoted as $d_{i}$. The position-wise error rate was calculated as:

(5) $$\displaystyle error\ rate_{i}=\frac{n_{i}}{d_{i}}$$

The representative error rate for a given sample (i.e. a specific PCR condition) was defined as the simple average across all positions in the analyzed region (length $L$):

(6) $$\displaystyle  error\ rate_{sample\ A}=\frac{1}{L}\sum \limits_{i=1}^{L}error\ rate_{i}$$

To characterize the mutation spectrum, substitution types were counted among identified mutations by tallying the frequency of each reference-to-alternate base change (e.g. A>C). In addition, for each reporter gene, primer-binding regions (19 bp) were defined based on the known primer sequences. Mutation rates and substitution spectra were calculated separately for primer-binding regions and non-primer regions. Primary comparisons were performed using profiles from non-primer regions, whereas primer-region profiles were used to assess the background mutation rate under low-mutation conditions (using KOD Plus Neo).

### tRNA-free PURE system (tfPURE) preparation

All components of the laboratory-made PURE system were prepared following previously established procedures. Briefly, individual factors were expressed with histidine tags and purified by affinity chromatography, followed by gel-filtration chromatography, as described previously (Miyachi et al., 2025). Additionally, EF-Tu was subjected to two successive rounds of affinity purification in a stringent buffer to eliminate contaminating tRNAs, as described previously (Miyachi et al., 2025; Miyachi et al., 2022). Ribosomes were purified by a butyl column and sucrose cushion method and further processed by ultrafiltration to remove residual tRNAs, using the same strategy as in our previous work (Miyachi et al., 2025; Miyachi et al., 2022). The complete composition and concentrations of tfPURE used in this study are provided in Appendix 1—table 6.

### Cell-free translation with reconstituted tRNAs

Cell-free translation reactions were performed using tfPURE supplemented with defined mixtures of in vitro-synthesized tRNAs and reporter gene DNA templates. The concentrations of DNA templates are specified in the corresponding figure legends, and the concentrations of tRNAs used for each genetic code are summarized in Appendix 1—table 7. Reaction mixtures were incubated at 30 °C for 16 hr.

Following incubation (or during incubation for mSG), reporter protein production was quantified by three different methods. For firefly luciferase (Luc) or NanoLuc, a 1 µL aliquot of the translation reaction was mixed with 30 µL of Luciferase Assay Reagent (Promega) or 50 µL of NanoLuc Assay Reagent (Promega), respectively, and luminescence was measured using a GloMax luminometer (Promega). For mSG, fluorescence signals were monitored continuously for up to 16 hr using an Mx3005P real-time PCR system (Agilent Technologies) during the translation reaction. The fluorescence intensity at 0 hr was subtracted from that at 16 hr, and the resulting difference was used as a measure of mSG activity. For GAL (Matsuura et al., 2011), a 1 µL aliquot of the translation reaction was added to 9 µL of GAL reaction buffer (final concentrations: 50 mM HEPES–KOH, pH 7.4; 1 mM MgCl_2_; 5 mM DTT; 5 µM TokyoGreen-βGal [Sekisui Medical, Japan]). Fluorescence was recorded at 1 min intervals for 60 min using an Mx3005P instrument with FAM detection settings. GAL activity was determined from the slope of the linear region of the fluorescence time course.

## Data Availability

Source data files and raw data have been deposited in Zenodo (https://doi.org/10.5281/zenodo.20364053). The following dataset was generated: IchihashiN 2026Experimental verification of the error minimization theory using non-standard genetic codes constructed in vitroZenodo10.5281/zenodo.2036405342441424

## Additional files

MDAR checklist

Source code 1. This code was used for the calculation of the cost of each genetic code.

## Appendix 1

**Appendix 1—table 1.** DNA sequences used in this study.

Template	Sequence
Nanoluc_32 C *Nannoluc gene	GGCGATTAAGTTGGGTAACGCCAGGGTTTTCCCAGTCACGACGTTGTAaAACGACGGCCAGTGAATTCTAATACGACTCACTATAGGGGAATTGTGAGCGGATAACAATTCCCCTCTAGAAATAATTTTGTTTAACTTTAAGAAGGAGATATACATATGGTTTTTACTCTGGAGGACTTTGTGGGTGACTGGCGGCAGACGGCTGGGTACAACTTGGACCAGGTTCTGGAGCAGGGTGGGGTGTCTTCGCTCTTTCAGAACCTGGGTGTTAGCGTGACTCCTATTCAGCGCATTGTTTTGTCTGGGGAGAACGGTCTGAAGATTGACATTCACGTGATTATTCCGTACGAGGGGCTCTCGGGTGACCAGATGGGGCAGATTGAGAAGATTTTTAAGGTTGTGTACCCTGTTGACGACCACCACTTTAAGGTGATTCTGCACTACGGTACGTTGGTTATTGACGGGGTGACTCCGAACATGATTGACTACTTTGGTAGGCCTTACGAGGGGATTGCGGTTTTTGACGGTAAGAAGATTACGGTGACTGGGACGCTGTGGAACGGTAACAAGATTATTGACGAGCGGCTCATTAACCCGGACGGGAGCCTGTTGTTTCGCGTTACTATTAACGGTGTGACGGGGTGGAGGCTGTGTGAGCGGATTCTCGCGTAAAAGCTTGGCGTAATCATGGTCATAGCTGTTTCCTGTGTGAAATTGTTATCCGCTCACAATTCCACACAACATACGAGCCGG
Nanoluc_46 C *Nannoluc gene	GGCGATTAAGTTGGGTAACGCCAGGGTTTTCCCAGTCACGACGTTGTAAAACGACGGCCAGTGAATTCTAATACGACTCACTATAGGGGAATTGTGAGCGGATAACAATTCCCCTCTAGAAATAATTTTGTTTAACTTTAAGAAGGAGATATACATATGGTTTTTACTCTGGAGGACTTTGTGGGTGACTGGCGGCAGACGGCTGGGTACAACTTGGACCAAGTATTAGAACAGGGAGGGGTTTCTTCGCTCTTTCAAAACCTAGGTGTGAGCGTAACACCTATTCAGCGCATAGTTTTATCAGGAGAGAACGGTCTGAAGATTGACATACACGTGATTATACCGTACGAAGGGCTCTCGGGAGACCAAATGGGGCAGATTGAGAAAATATTTAAGGTAGTTTACCCAGTGGACGACCACCACTTTAAAGTAATTCTACACTACGGTACGTTGGTTATAGACGGAGTGACTCCGAACATGATTGACTACTTTGGTAGGCCTTACGAAGGGATAGCAGTATTTGACGGAAAGAAAATTACAGTTACTGGGACGCTGTGGAACGGTAACAAGATAATTGACGAGCGATTAATAAACCCAGACGGATCACTGTTGTTTAGAGTGACTATTAACGGTGTAACAGGGTGGCGACTATGTGAAAGAATACTCGCGTAAAAGCTTGGCGTAATCATGGTCATAGCTGTTTCCTGTGTGAAATTGTTATCCGCTCACAATTCCACACAACATACGAGCCGG
Nanoluc_21 C *Nannoluc gene	GGCGATTAAGTTGGGTAACGCCAGGGTTTTCCCAGTCACGACGTTGTAAAACGACGGCCAGTGAATTCTAATACGACTCACTATAGGGGAATTGTGAGCGGATAACAATTCCCCTCTAGAAATAATTTTGTTTAACTTTAAGAAGGAGATATACATATGGTTTTTACTCTGGAGGACTTTGTTGGTGACTGGCGGCAGACTGCTGGTTACAACCTGGACCAGGTTCTGGAGCAGGGTGGTGTTTCTTCTCTGTTTCAGAACCTGGGTGTTTCTGTTACTCCTATTCAGCGGATTGTTCTGTCTGGTGAGAACGGTCTGAAGATTGACATTCACGTTATTATTCCTTACGAGGGTCTGTCTGGTGACCAGATGGGTCAGATTGAGAAGATTTTTAAGGTTGTTTACCCTGTTGACGACCACCACTTTAAGGTTATTCTGCACTACGGTACTCTGGTTATTGACGGTGTTACTCCTAACATGATTGACTACTTTGGTCGGCCTTACGAGGGTATTGCTGTTTTTGACGGTAAGAAGATTACTGTTACTGGTACTCTGTGGAACGGTAACAAGATTATTGACGAGCGGCTGATTAACCCTGACGGTTCTCTGCTGTTTCGGGTTACTATTAACGGTGTTACTGGTTGGCGGCTGTGTGAGCGGATTCTGGCTTAAAAGCTTGGCGTAATCATGGTCATAGCTGTTTCCTGTGTGAAATTGTTATCCGCTCACAATTCCACACAACATACGAGCCGG
Nanoluc_21+Ala_CAA *Nannoluc gene	GGCGATTAAGTTGGGTAACGCCAGGGTTTTCCCAGTCACGACGTTGTAAAACGACGGCCAGTGAATTCTAATACGACTCACTATAGGGGAATTGTGAGCGGATAACAATTCCCCTCTAGAAATAATTTTGTTTAACTTTAAGAAGGAGATATACATATGGTTTTTACTCTCGAGGACTTTGTTGGTGACTGGCGCCAGACTTTGGGTTACAACCTCGACCAGGTTCTCGAGCAGGGTGGTGTTTCTTCTCTCTTTCAGAACCTCGGTGTTTCTGTTACTCCTATTCAGCGCATTGTTCTCTCTGGTGAGAACGGTCTCAAGATTGACATTCACGTTATTATTCCTTACGAGGGTCTCTCTGGTGACCAGATGGGTCAGATTGAGAAGATTTTTAAGGTTGTTTACCCTGTTGACGACCACCACTTTAAGGTTATTCTCCACTACGGTACTCTCGTTATTGACGGTGTTACTCCTAACATGATTGACTACTTTGGTCGCCCTTACGAGGGTATTTTGGTTTTTGACGGTAAGAAGATTACTGTTACTGGTACTCTCTGGAACGGTAACAAGATTATTGACGAGCGCCTCATTAACCCTGACGGTTCTCTCCTCTTTCGCGTTACTATTAACGGTGTTACTGGTTGGCGCCTCTGTGAGCGCATTCTCGCTTAAAAGCTTGGCGTAATCATGGTCATAGCTGTTTCCTGTGTGAAATTGTTATCCGCTCACAATTCCACACAACATACGAGCCGG
Nanoluc_21+Ala_CGA *Nannoluc gene	GGCGATTAAGTTGGGTAACGCCAGGGTTTTCCCAGTCACGACGTTGTAAAACGACGGCCAGTGAATTCTAATACGACTCACTATAGGGGAATTGTGAGCGGATAACAATTCCCCTCTAGAAATAATTTTGTTTAACTTTAAGAAGGAGATATACATATGGTTTTTACTCTCGAGGACTTTGTTGGTGACTGGCGCCAGACTTCGGGTTACAACCTCGACCAGGTTCTCGAGCAGGGTGGTGTTTCTTCTCTCTTTCAGAACCTCGGTGTTTCTGTTACTCCTATTCAGCGCATTGTTCTCTCTGGTGAGAACGGTCTCAAGATTGACATTCACGTTATTATTCCTTACGAGGGTCTCTCTGGTGACCAGATGGGTCAGATTGAGAAGATTTTTAAGGTTGTTTACCCTGTTGACGACCACCACTTTAAGGTTATTCTCCACTACGGTACTCTCGTTATTGACGGTGTTACTCCTAACATGATTGACTACTTTGGTCGCCCTTACGAGGGTATTTCGGTTTTTGACGGTAAGAAGATTACTGTTACTGGTACTCTCTGGAACGGTAACAAGATTATTGACGAGCGCCTCATTAACCCTGACGGTTCTCTCCTCTTTCGCGTTACTATTAACGGTGTTACTGGTTGGCGCCTCTGTGAGCGCATTCTCGCTTAAAAGCTTGGCGTAATCATGGTCATAGCTGTTTCCTGTGTGAAATTGTTATCCGCTCACAATTCCACACAACATACGAGCCGG
Nanoluc_21+Ala_CAG *Nannoluc gene	GGCGATTAAGTTGGGTAACGCCAGGGTTTTCCCAGTCACGACGTTGTAAAACGACGGCCAGTGAATTCTAATACGACTCACTATAGGGGAATTGTGAGCGGATAACAATTCCCCTCTAGAAATAATTTTGTTTAACTTTAAGAAGGAGATATACATATGGTTTTTACTCTCGAGGACTTTGTTGGTGACTGGCGCCAGACTCTGGGTTACAACCTCGACCAGGTTCTCGAGCAGGGTGGTGTTTCTTCTCTCTTTCAGAACCTCGGTGTTTCTGTTACTCCTATTCAGCGCATTGTTCTCTCTGGTGAGAACGGTCTCAAGATTGACATTCACGTTATTATTCCTTACGAGGGTCTCTCTGGTGACCAGATGGGTCAGATTGAGAAGATTTTTAAGGTTGTTTACCCTGTTGACGACCACCACTTTAAGGTTATTCTCCACTACGGTACTCTCGTTATTGACGGTGTTACTCCTAACATGATTGACTACTTTGGTCGCCCTTACGAGGGTATTCTGGTTTTTGACGGTAAGAAGATTACTGTTACTGGTACTCTCTGGAACGGTAACAAGATTATTGACGAGCGCCTCATTAACCCTGACGGTTCTCTCCTCTTTCGCGTTACTATTAACGGTGTTACTGGTTGGCGCCTCTGTGAGCGCATTCTCGCTTAAAAGCTTGGCGTAATCATGGTCATAGCTGTTTCCTGTGTGAAATTGTTATCCGCTCACAATTCCACACAACATACGAGCCGG
Nanoluc_21+Ala_CGG *Nannoluc gene	GGCGATTAAGTTGGGTAACGCCAGGGTTTTCCCAGTCACGACGTTGTAAAACGACGGCCAGTGAATTCTAATACGACTCACTATAGGGGAATTGTGAGCGGATAACAATTCCCCTCTAGAAATAATTTTGTTTAACTTTAAGAAGGAGATATACATATGGTTTTTACTCTCGAGGACTTTGTTGGTGACTGGCGCCAGACTCCGGGTTACAACCTCGACCAGGTTCTCGAGCAGGGTGGTGTTTCTTCTCTCTTTCAGAACCTCGGTGTTTCTGTTACTCCTATTCAGCGCATTGTTCTCTCTGGTGAGAACGGTCTCAAGATTGACATTCACGTTATTATTCCTTACGAGGGTCTCTCTGGTGACCAGATGGGTCAGATTGAGAAGATTTTTAAGGTTGTTTACCCTGTTGACGACCACCACTTTAAGGTTATTCTCCACTACGGTACTCTCGTTATTGACGGTGTTACTCCTAACATGATTGACTACTTTGGTCGCCCTTACGAGGGTATTCCGGTTTTTGACGGTAAGAAGATTACTGTTACTGGTACTCTCTGGAACGGTAACAAGATTATTGACGAGCGCCTCATTAACCCTGACGGTTCTCTCCTCTTTCGCGTTACTATTAACGGTGTTACTGGTTGGCGCCTCTGTGAGCGCATTCTCGCTTAAAAGCTTGGCGTAATCATGGTCATAGCTGTTTCCTGTGTGAAATTGTTATCCGCTCACAATTCCACACAACATACGAGCCGG
Nanoluc_21+Ala_CCG *Nannoluc gene	GGCGATTAAGTTGGGTAACGCCAGGGTTTTCCCAGTCACGACGTTGTAAAACGACGGCCAGTGAATTCTAATACGACTCACTATAGGGGAATTGTGAGCGGATAACAATTCCCCTCTAGAAATAATTTTGTTTAACTTTAAGAAGGAGATATACATATGGTTTTTACTCTCGAGGACTTTGTTGGTGACTGGCGCCAGACTCGGGGTTACAACCTCGACCAGGTTCTCGAGCAGGGTGGTGTTTCTTCTCTCTTTCAGAACCTCGGTGTTTCTGTTACTCCTATTCAGCGCATTGTTCTCTCTGGTGAGAACGGTCTCAAGATTGACATTCACGTTATTATTCCTTACGAGGGTCTCTCTGGTGACCAGATGGGTCAGATTGAGAAGATTTTTAAGGTTGTTTACCCTGTTGACGACCACCACTTTAAGGTTATTCTCCACTACGGTACTCTCGTTATTGACGGTGTTACTCCTAACATGATTGACTACTTTGGTCGCCCTTACGAGGGTATTCGGGTTTTTGACGGTAAGAAGATTACTGTTACTGGTACTCTCTGGAACGGTAACAAGATTATTGACGAGCGCCTCATTAACCCTGACGGTTCTCTCCTCTTTCGCGTTACTATTAACGGTGTTACTGGTTGGCGCCTCTGTGAGCGCATTCTCGCTTAAAAGCTTGGCGTAATCATGGTCATAGCTGTTTCCTGTGTGAAATTGTTATCCGCTCACAATTCCACACAACATACGAGCCGG
Nanoluc_21+Ala_CGU *Nannoluc gene	GGCGATTAAGTTGGGTAACGCCAGGGTTTTCCCAGTCACGACGTTGTAAAACGACGGCCAGTGAATTCTAATACGACTCACTATAGGGGAATTGTGAGCGGATAACAATTCCCCTCTAGAAATAATTTTGTTTAACTTTAAGAAGGAGATATACATATGGTTTTTACTCTCGAGGACTTTGTTGGTGACTGGCGCCAGACTACGGGTTACAACCTCGACCAGGTTCTCGAGCAGGGTGGTGTTTCTTCTCTCTTTCAGAACCTCGGTGTTTCTGTTACTCCTATTCAGCGCATTGTTCTCTCTGGTGAGAACGGTCTCAAGATTGACATTCACGTTATTATTCCTTACGAGGGTCTCTCTGGTGACCAGATGGGTCAGATTGAGAAGATTTTTAAGGTTGTTTACCCTGTTGACGACCACCACTTTAAGGTTATTCTCCACTACGGTACTCTCGTTATTGACGGTGTTACTCCTAACATGATTGACTACTTTGGTCGCCCTTACGAGGGTATTACGGTTTTTGACGGTAAGAAGATTACTGTTACTGGTACTCTCTGGAACGGTAACAAGATTATTGACGAGCGCCTCATTAACCCTGACGGTTCTCTCCTCTTTCGCGTTACTATTAACGGTGTTACTGGTTGGCGCCTCTGTGAGCGCATTCTCGCTTAAAAGCTTGGCGTAATCATGGTCATAGCTGTTTCCTGTGTGAAATTGTTATCCGCTCACAATTCCACACAACATACGAGCCGG
Nanoluc_21+Ala_GCU *Nannoluc gene	GGCGATTAAGTTGGGTAACGCCAGGGTTTTCCCAGTCACGACGTTGTAAAACGACGGCCAGTGAATTCTAATACGACTCACTATAGGGGAATTGTGAGCGGATAACAATTCCCCTCTAGAAATAATTTTGTTTAACTTTAAGAAGGAGATATACATATGGTTTTTACTCTCGAGGACTTTGTTGGTGACTGGCGCCAGACTAGCGGTTACAACCTCGACCAGGTTCTCGAGCAGGGTGGTGTTTCTTCTCTCTTTCAGAACCTCGGTGTTTCTGTTACTCCTATTCAGCGCATTGTTCTCTCTGGTGAGAACGGTCTCAAGATTGACATTCACGTTATTATTCCTTACGAGGGTCTCTCTGGTGACCAGATGGGTCAGATTGAGAAGATTTTTAAGGTTGTTTACCCTGTTGACGACCACCACTTTAAGGTTATTCTCCACTACGGTACTCTCGTTATTGACGGTGTTACTCCTAACATGATTGACTACTTTGGTCGCCCTTACGAGGGTATTAGCGTTTTTGACGGTAAGAAGATTACTGTTACTGGTACTCTCTGGAACGGTAACAAGATTATTGACGAGCGCCTCATTAACCCTGACGGTTCTCTCCTCTTTCGCGTTACTATTAACGGTGTTACTGGTTGGCGCCTCTGTGAGCGCATTCTCGCTTAAAAGCTTGGCGTAATCATGGTCATAGCTGTTTCCTGTGTGAAATTGTTATCCGCTCACAATTCCACACAACATACGAGCCGG
Nanoluc_21+Ala_CAC *Nannoluc gene	GGCGATTAAGTTGGGTAACGCCAGGGTTTTCCCAGTCACGACGTTGTAAAACGACGGCCAGTGAATTCTAATACGACTCACTATAGGGGAATTGTGAGCGGATAACAATTCCCCTCTAGAAATAATTTTGTTTAACTTTAAGAAGGAGATATACATATGGTTTTTACTCTCGAGGACTTTGTTGGTGACTGGCGCCAGACTGTGGGTTACAACCTCGACCAGGTTCTCGAGCAGGGTGGTGTTTCTTCTCTCTTTCAGAACCTCGGTGTTTCTGTTACTCCTATTCAGCGCATTGTTCTCTCTGGTGAGAACGGTCTCAAGATTGACATTCACGTTATTATTCCTTACGAGGGTCTCTCTGGTGACCAGATGGGTCAGATTGAGAAGATTTTTAAGGTTGTTTACCCTGTTGACGACCACCACTTTAAGGTTATTCTCCACTACGGTACTCTCGTTATTGACGGTGTTACTCCTAACATGATTGACTACTTTGGTCGCCCTTACGAGGGTATTGTGGTTTTTGACGGTAAGAAGATTACTGTTACTGGTACTCTCTGGAACGGTAACAAGATTATTGACGAGCGCCTCATTAACCCTGACGGTTCTCTCCTCTTTCGCGTTACTATTAACGGTGTTACTGGTTGGCGCCTCTGTGAGCGCATTCTCGCTTAAAAGCTTGGCGTAATCATGGTCATAGCTGTTTCCTGTGTGAAATTGTTATCCGCTCACAATTCCACACAACATACGAGCCGG
Nanoluc_21+Ser_CAA *Nannoluc gene	GGCGATTAAGTTGGGTAACGCCAGGGTTTTCCCAGTCACGACGTTGTAAAACGACGGCCAGTGAATTCTAATACGACTCACTATAGGGGAATTGTGAGCGGATAACAATTCCCCTCTAGAAATAATTTTGTTTAACTTTAAGAAGGAGATATACATATGGTTTTTACTCTCGAGGACTTTGTTGGTGACTGGCGCCAGACTGCTGGTTACAACCTCGACCAGGTTCTCGAGCAGGGTGGTGTTTCTTTGCTCTTTCAGAACCTCGGTGTTTCTGTTACTCCTATTCAGCGCATTGTTCTCTTGGGTGAGAACGGTCTCAAGATTGACATTCACGTTATTATTCCTTACGAGGGTCTCTCTGGTGACCAGATGGGTCAGATTGAGAAGATTTTTAAGGTTGTTTACCCTGTTGACGACCACCACTTTAAGGTTATTCTCCACTACGGTACTCTCGTTATTGACGGTGTTACTCCTAACATGATTGACTACTTTGGTCGCCCTTACGAGGGTATTGCTGTTTTTGACGGTAAGAAGATTACTGTTACTGGTACTCTCTGGAACGGTAACAAGATTATTGACGAGCGCCTCATTAACCCTGACGGTTTGCTCCTCTTTCGCGTTACTATTAACGGTGTTACTGGTTGGCGCCTCTGTGAGCGCATTCTCGCTTAAAAGCTTGGCGTAATCATGGTCATAGCTGTTTCCTGTGTGAAATTGTTATCCGCTCACAATTCCACACAACATACGAGCCGG
Nanoluc_21+Ser_CAG *Nannoluc gene	GGCGATTAAGTTGGGTAACGCCAGGGTTTTCCCAGTCACGACGTTGTAAAACGACGGCCAGTGAATTCTAATACGACTCACTATAGGGGAATTGTGAGCGGATAACAATTCCCCTCTAGAAATAATTTTGTTTAACTTTAAGAAGGAGATATACATATGGTTTTTACTCTCGAGGACTTTGTTGGTGACTGGCGCCAGACTGCTGGTTACAACCTCGACCAGGTTCTCGAGCAGGGTGGTGTTTCTCTGCTCTTTCAGAACCTCGGTGTTTCTGTTACTCCTATTCAGCGCATTGTTCTCCTGGGTGAGAACGGTCTCAAGATTGACATTCACGTTATTATTCCTTACGAGGGTCTCTCTGGTGACCAGATGGGTCAGATTGAGAAGATTTTTAAGGTTGTTTACCCTGTTGACGACCACCACTTTAAGGTTATTCTCCACTACGGTACTCTCGTTATTGACGGTGTTACTCCTAACATGATTGACTACTTTGGTCGCCCTTACGAGGGTATTGCTGTTTTTGACGGTAAGAAGATTACTGTTACTGGTACTCTCTGGAACGGTAACAAGATTATTGACGAGCGCCTCATTAACCCTGACGGTCTGCTCCTCTTTCGCGTTACTATTAACGGTGTTACTGGTTGGCGCCTCTGTGAGCGCATTCTCGCTTAAAAGCTTGGCGTAATCATGGTCATAGCTGTTTCCTGTGTGAAATTGTTATCCGCTCACAATTCCACACAACATACGAGCCGG
Nanoluc_21+Ser_CGG *Nannoluc gene	GGCGATTAAGTTGGGTAACGCCAGGGTTTTCCCAGTCACGACGTTGTAAAACGACGGCCAGTGAATTCTAATACGACTCACTATAGGGGAATTGTGAGCGGATAACAATTCCCCTCTAGAAATAATTTTGTTTAACTTTAAGAAGGAGATATACATATGGTTTTTACTCTCGAGGACTTTGTTGGTGACTGGCGCCAGACTGCTGGTTACAACCTCGACCAGGTTCTCGAGCAGGGTGGTGTTTCTCCGCTCTTTCAGAACCTCGGTGTTTCTGTTACTCCTATTCAGCGCATTGTTCTCCCGGGTGAGAACGGTCTCAAGATTGACATTCACGTTATTATTCCTTACGAGGGTCTCTCTGGTGACCAGATGGGTCAGATTGAGAAGATTTTTAAGGTTGTTTACCCTGTTGACGACCACCACTTTAAGGTTATTCTCCACTACGGTACTCTCGTTATTGACGGTGTTACTCCTAACATGATTGACTACTTTGGTCGCCCTTACGAGGGTATTGCTGTTTTTGACGGTAAGAAGATTACTGTTACTGGTACTCTCTGGAACGGTAACAAGATTATTGACGAGCGCCTCATTAACCCTGACGGTCCGCTCCTCTTTCGCGTTACTATTAACGGTGTTACTGGTTGGCGCCTCTGTGAGCGCATTCTCGCTTAAAAGCTTGGCGTAATCATGGTCATAGCTGTTTCCTGTGTGAAATTGTTATCCGCTCACAATTCCACACAACATACGAGCCGG
Nanoluc_21+Ser_CCG *Nannoluc gene	GGCGATTAAGTTGGGTAACGCCAGGGTTTTCCCAGTCACGACGTTGTAAAACGACGGCCAGTGAATTCTAATACGACTCACTATAGGGGAATTGTGAGCGGATAACAATTCCCCTCTAGAAATAATTTTGTTTAACTTTAAGAAGGAGATATACATATGGTTTTTACTCTCGAGGACTTTGTTGGTGACTGGCGCCAGACTGCTGGTTACAACCTCGACCAGGTTCTCGAGCAGGGTGGTGTTTCTCGGCTCTTTCAGAACCTCGGTGTTTCTGTTACTCCTATTCAGCGCATTGTTCTCCGGGGTGAGAACGGTCTCAAGATTGACATTCACGTTATTATTCCTTACGAGGGTCTCTCTGGTGACCAGATGGGTCAGATTGAGAAGATTTTTAAGGTTGTTTACCCTGTTGACGACCACCACTTTAAGGTTATTCTCCACTACGGTACTCTCGTTATTGACGGTGTTACTCCTAACATGATTGACTACTTTGGTCGCCCTTACGAGGGTATTGCTGTTTTTGACGGTAAGAAGATTACTGTTACTGGTACTCTCTGGAACGGTAACAAGATTATTGACGAGCGCCTCATTAACCCTGACGGTCGGCTCCTCTTTCGCGTTACTATTAACGGTGTTACTGGTTGGCGCCTCTGTGAGCGCATTCTCGCTTAAAAGCTTGGCGTAATCATGGTCATAGCTGTTTCCTGTGTGAAATTGTTATCCGCTCACAATTCCACACAACATACGAGCCGG
Nanoluc_21+Ser_CGU *Nannoluc gene	GGCGATTAAGTTGGGTAACGCCAGGGTTTTCCCAGTCACGACGTTGTAAAACGACGGCCAGTGAATTCTAATACGACTCACTATAGGGGAATTGTGAGCGGATAACAATTCCCCTCTAGAAATAATTTTGTTTAACTTTAAGAAGGAGATATACATATGGTTTTTACTCTCGAGGACTTTGTTGGTGACTGGCGCCAGACTGCTGGTTACAACCTCGACCAGGTTCTCGAGCAGGGTGGTGTTTCTACGCTCTTTCAGAACCTCGGTGTTTCTGTTACTCCTATTCAGCGCATTGTTCTCACGGGTGAGAACGGTCTCAAGATTGACATTCACGTTATTATTCCTTACGAGGGTCTCTCTGGTGACCAGATGGGTCAGATTGAGAAGATTTTTAAGGTTGTTTACCCTGTTGACGACCACCACTTTAAGGTTATTCTCCACTACGGTACTCTCGTTATTGACGGTGTTACTCCTAACATGATTGACTACTTTGGTCGCCCTTACGAGGGTATTGCTGTTTTTGACGGTAAGAAGATTACTGTTACTGGTACTCTCTGGAACGGTAACAAGATTATTGACGAGCGCCTCATTAACCCTGACGGTACGCTCCTCTTTCGCGTTACTATTAACGGTGTTACTGGTTGGCGCCTCTGTGAGCGCATTCTCGCTTAAAAGCTTGGCGTAATCATGGTCATAGCTGTTTCCTGTGTGAAATTGTTATCCGCTCACAATTCCACACAACATACGAGCCGG
Nanoluc_21+Ser_CAC *Nannoluc gene	GGCGATTAAGTTGGGTAACGCCAGGGTTTTCCCAGTCACGACGTTGTAAAACGACGGCCAGTGAATTCTAATACGACTCACTATAGGGGAATTGTGAGCGGATAACAATTCCCCTCTAGAAATAATTTTGTTTAACTTTAAGAAGGAGATATACATATGGTTTTTACTCTCGAGGACTTTGTTGGTGACTGGCGCCAGACTGCTGGTTACAACCTCGACCAGGTTCTCGAGCAGGGTGGTGTTTCTGTGCTCTTTCAGAACCTCGGTGTTTCTGTTACTCCTATTCAGCGCATTGTTCTCGTGGGTGAGAACGGTCTCAAGATTGACATTCACGTTATTATTCCTTACGAGGGTCTCTCTGGTGACCAGATGGGTCAGATTGAGAAGATTTTTAAGGTTGTTTACCCTGTTGACGACCACCACTTTAAGGTTATTCTCCACTACGGTACTCTCGTTATTGACGGTGTTACTCCTAACATGATTGACTACTTTGGTCGCCCTTACGAGGGTATTGCTGTTTTTGACGGTAAGAAGATTACTGTTACTGGTACTCTCTGGAACGGTAACAAGATTATTGACGAGCGCCTCATTAACCCTGACGGTGTGCTCCTCTTTCGCGTTACTATTAACGGTGTTACTGGTTGGCGCCTCTGTGAGCGCATTCTCGCTTAAAAGCTTGGCGTAATCATGGTCATAGCTGTTTCCTGTGTGAAATTGTTATCCGCTCACAATTCCACACAACATACGAGCCGG
Nanoluc_21+Ser_CGC *Nannoluc gene	GGCGATTAAGTTGGGTAACGCCAGGGTTTTCCCAGTCACGACGTTGTAAAACGACGGCCAGTGAATTCTAATACGACTCACTATAGGGGAATTGTGAGCGGATAACAATTCCCCTCTAGAAATAATTTTGTTTAACTTTAAGAAGGAGATATACATATGGTTTTTACTCTCGAGGACTTTGTTGGTGACTGGCGCCAGACTGCTGGTTACAACCTCGACCAGGTTCTCGAGCAGGGTGGTGTTTCTGCGCTCTTTCAGAACCTCGGTGTTTCTGTTACTCCTATTCAGCGCATTGTTCTCGCGGGTGAGAACGGTCTCAAGATTGACATTCACGTTATTATTCCTTACGAGGGTCTCTCTGGTGACCAGATGGGTCAGATTGAGAAGATTTTTAAGGTTGTTTACCCTGTTGACGACCACCACTTTAAGGTTATTCTCCACTACGGTACTCTCGTTATTGACGGTGTTACTCCTAACATGATTGACTACTTTGGTCGCCCTTACGAGGGTATTGCTGTTTTTGACGGTAAGAAGATTACTGTTACTGGTACTCTCTGGAACGGTAACAAGATTATTGACGAGCGCCTCATTAACCCTGACGGTGCGCTCCTCTTTCGCGTTACTATTAACGGTGTTACTGGTTGGCGCCTCTGTGAGCGCATTCTCGCTTAAAAGCTTGGCGTAATCATGGTCATAGCTGTTTCCTGTGTGAAATTGTTATCCGCTCACAATTCCACACAACATACGAGCCGG
Nanoluc_21+Leu_CGA *Nannoluc gene	GGCGATTAAGTTGGGTAACGCCAGGGTTTTCCCAGTCACGACGTTGTAAAACGACGGCCAGTGAATTCTAATACGACTCACTATAGGGGAATTGTGAGCGGATAACAATTCCCCTCTAGAAATAATTTTGTTTAACTTTAAGAAGGAGATATACATATGGTTTTTACTCTCGAGGACTTTGTTGGTGACTGGCGCCAGACTGCTGGTTACAACCTCGACCAGGTTCTCGAGCAGGGTGGTGTTTCTTCTTCGTTTCAGAACCTCGGTGTTTCTGTTACTCCTATTCAGCGCATTGTTCTCTCTGGTGAGAACGGTCTCAAGATTGACATTCACGTTATTATTCCTTACGAGGGTTCGTCTGGTGACCAGATGGGTCAGATTGAGAAGATTTTTAAGGTTGTTTACCCTGTTGACGACCACCACTTTAAGGTTATTCTCCACTACGGTACTCTCGTTATTGACGGTGTTACTCCTAACATGATTGACTACTTTGGTCGCCCTTACGAGGGTATTGCTGTTTTTGACGGTAAGAAGATTACTGTTACTGGTACTCTCTGGAACGGTAACAAGATTATTGACGAGCGCTCGATTAACCCTGACGGTTCTCTCCTCTTTCGCGTTACTATTAACGGTGTTACTGGTTGGCGCCTCTGTGAGCGCATTTCGGCTTAAAAGCTTGGCGTAATCATGGTCATAGCTGTTTCCTGTGTGAAATTGTTATCCGCTCACAATTCCACACAACATACGAGCCGG
Nanoluc_21+Leu_CGG *Nannoluc gene	GGCGATTAAGTTGGGTAACGCCAGGGTTTTCCCAGTCACGACGTTGTAAAACGACGGCCAGTGAATTCTAATACGACTCACTATAGGGGAATTGTGAGCGGATAACAATTCCCCTCTAGAAATAATTTTGTTTAACTTTAAGAAGGAGATATACATATGGTTTTTACTCTCGAGGACTTTGTTGGTGACTGGCGCCAGACTGCTGGTTACAACCTCGACCAGGTTCTCGAGCAGGGTGGTGTTTCTTCTCCGTTTCAGAACCTCGGTGTTTCTGTTACTCCTATTCAGCGCATTGTTCTCTCTGGTGAGAACGGTCTCAAGATTGACATTCACGTTATTATTCCTTACGAGGGTCCGTCTGGTGACCAGATGGGTCAGATTGAGAAGATTTTTAAGGTTGTTTACCCTGTTGACGACCACCACTTTAAGGTTATTCTCCACTACGGTACTCTCGTTATTGACGGTGTTACTCCTAACATGATTGACTACTTTGGTCGCCCTTACGAGGGTATTGCTGTTTTTGACGGTAAGAAGATTACTGTTACTGGTACTCTCTGGAACGGTAACAAGATTATTGACGAGCGCCCGATTAACCCTGACGGTTCTCTCCTCTTTCGCGTTACTATTAACGGTGTTACTGGTTGGCGCCTCTGTGAGCGCATTCCGGCTTAAAAGCTTGGCGTAATCATGGTCATAGCTGTTTCCTGTGTGAAATTGTTATCCGCTCACAATTCCACACAACATACGAGCCGG
Nanoluc_21+Leu_CCG *Nannoluc gene	GGCGATTAAGTTGGGTAACGCCAGGGTTTTCCCAGTCACGACGTTGTAAAACGACGGCCAGTGAATTCTAATACGACTCACTATAGGGGAATTGTGAGCGGATAACAATTCCCCTCTAGAAATAATTTTGTTTAACTTTAAGAAGGAGATATACATATGGTTTTTACTCTCGAGGACTTTGTTGGTGACTGGCGCCAGACTGCTGGTTACAACCTCGACCAGGTTCTCGAGCAGGGTGGTGTTTCTTCTCGGTTTCAGAACCTCGGTGTTTCTGTTACTCCTATTCAGCGCATTGTTCTCTCTGGTGAGAACGGTCTCAAGATTGACATTCACGTTATTATTCCTTACGAGGGTCGGTCTGGTGACCAGATGGGTCAGATTGAGAAGATTTTTAAGGTTGTTTACCCTGTTGACGACCACCACTTTAAGGTTATTCTCCACTACGGTACTCTCGTTATTGACGGTGTTACTCCTAACATGATTGACTACTTTGGTCGCCCTTACGAGGGTATTGCTGTTTTTGACGGTAAGAAGATTACTGTTACTGGTACTCTCTGGAACGGTAACAAGATTATTGACGAGCGCCGGATTAACCCTGACGGTTCTCTCCTCTTTCGCGTTACTATTAACGGTGTTACTGGTTGGCGCCTCTGTGAGCGCATTCGGGCTTAAAAGCTTGGCGTAATCATGGTCATAGCTGTTTCCTGTGTGAAATTGTTATCCGCTCACAATTCCACACAACATACGAGCCGG
Nanoluc_21+Leu_GCU *Nannoluc gene	GGCGATTAAGTTGGGTAACGCCAGGGTTTTCCCAGTCACGACGTTGTAAAACGACGGCCAGTGAATTCTAATACGACTCACTATAGGGGAATTGTGAGCGGATAACAATTCCCCTCTAGAAATAATTTTGTTTAACTTTAAGAAGGAGATATACATATGGTTTTTACTCTCGAGGACTTTGTTGGTGACTGGCGCCAGACTGCTGGTTACAACCTCGACCAGGTTCTCGAGCAGGGTGGTGTTTCTTCTAGCTTTCAGAACCTCGGTGTTTCTGTTACTCCTATTCAGCGCATTGTTCTCTCTGGTGAGAACGGTCTCAAGATTGACATTCACGTTATTATTCCTTACGAGGGTAGCTCTGGTGACCAGATGGGTCAGATTGAGAAGATTTTTAAGGTTGTTTACCCTGTTGACGACCACCACTTTAAGGTTATTCTCCACTACGGTACTCTCGTTATTGACGGTGTTACTCCTAACATGATTGACTACTTTGGTCGCCCTTACGAGGGTATTGCTGTTTTTGACGGTAAGAAGATTACTGTTACTGGTACTCTCTGGAACGGTAACAAGATTATTGACGAGCGCAGCATTAACCCTGACGGTTCTCTCCTCTTTCGCGTTACTATTAACGGTGTTACTGGTTGGCGCCTCTGTGAGCGCATTAGCGCTTAAAAGCTTGGCGTAATCATGGTCATAGCTGTTTCCTGTGTGAAATTGTTATCCGCTCACAATTCCACACAACATACGAGCCGG
Nanoluc_21+Leu_CGU *Nannoluc gene	GGCGATTAAGTTGGGTAACGCCAGGGTTTTCCCAGTCACGACGTTGTAAAACGACGGCCAGTGAATTCTAATACGACTCACTATAGGGGAATTGTGAGCGGATAACAATTCCCCTCTAGAAATAATTTTGTTTAACTTTAAGAAGGAGATATACATATGGTTTTTACTCTCGAGGACTTTGTTGGTGACTGGCGCCAGACTGCTGGTTACAACCTCGACCAGGTTCTCGAGCAGGGTGGTGTTTCTTCTACGTTTCAGAACCTCGGTGTTTCTGTTACTCCTATTCAGCGCATTGTTCTCTCTGGTGAGAACGGTCTCAAGATTGACATTCACGTTATTATTCCTTACGAGGGTACGTCTGGTGACCAGATGGGTCAGATTGAGAAGATTTTTAAGGTTGTTTACCCTGTTGACGACCACCACTTTAAGGTTATTCTCCACTACGGTACTCTCGTTATTGACGGTGTTACTCCTAACATGATTGACTACTTTGGTCGCCCTTACGAGGGTATTGCTGTTTTTGACGGTAAGAAGATTACTGTTACTGGTACTCTCTGGAACGGTAACAAGATTATTGACGAGCGCACGATTAACCCTGACGGTTCTCTCCTCTTTCGCGTTACTATTAACGGTGTTACTGGTTGGCGCCTCTGTGAGCGCATTACGGCTTAAAAGCTTGGCGTAATCATGGTCATAGCTGTTTCCTGTGTGAAATTGTTATCCGCTCACAATTCCACACAACATACGAGCCGG
Nanoluc_21+Leu_CAC *Nannoluc gene	GGCGATTAAGTTGGGTAACGCCAGGGTTTTCCCAGTCACGACGTTGTAAAACGACGGCCAGTGAATTCTAATACGACTCACTATAGGGGAATTGTGAGCGGATAACAATTCCCCTCTAGAAATAATTTTGTTTAACTTTAAGAAGGAGATATACATATGGTTTTTACTCTCGAGGACTTTGTTGGTGACTGGCGCCAGACTGCTGGTTACAACCTCGACCAGGTTCTCGAGCAGGGTGGTGTTTCTTCTGTGTTTCAGAACCTCGGTGTTTCTGTTACTCCTATTCAGCGCATTGTTCTCTCTGGTGAGAACGGTCTCAAGATTGACATTCACGTTATTATTCCTTACGAGGGTGTGTCTGGTGACCAGATGGGTCAGATTGAGAAGATTTTTAAGGTTGTTTACCCTGTTGACGACCACCACTTTAAGGTTATTCTCCACTACGGTACTCTCGTTATTGACGGTGTTACTCCTAACATGATTGACTACTTTGGTCGCCCTTACGAGGGTATTGCTGTTTTTGACGGTAAGAAGATTACTGTTACTGGTACTCTCTGGAACGGTAACAAGATTATTGACGAGCGCGTGATTAACCCTGACGGTTCTCTCCTCTTTCGCGTTACTATTAACGGTGTTACTGGTTGGCGCCTCTGTGAGCGCATTGTGGCTTAAAAGCTTGGCGTAATCATGGTCATAGCTGTTTCCTGTGTGAAATTGTTATCCGCTCACAATTCCACACAACATACGAGCCGG
Nanoluc_21+Leu_CGC *Nannoluc gene	GGCGATTAAGTTGGGTAACGCCAGGGTTTTCCCAGTCACGACGTTGTAAAACGACGGCCAGTGAATTCTAATACGACTCACTATAGGGGAATTGTGAGCGGATAACAATTCCCCTCTAGAAATAATTTTGTTTAACTTTAAGAAGGAGATATACATATGGTTTTTACTCTCGAGGACTTTGTTGGTGACTGGCGCCAGACTGCTGGTTACAACCTCGACCAGGTTCTCGAGCAGGGTGGTGTTTCTTCTGCGTTTCAGAACCTCGGTGTTTCTGTTACTCCTATTCAGCGCATTGTTCTCTCTGGTGAGAACGGTCTCAAGATTGACATTCACGTTATTATTCCTTACGAGGGTGCGTCTGGTGACCAGATGGGTCAGATTGAGAAGATTTTTAAGGTTGTTTACCCTGTTGACGACCACCACTTTAAGGTTATTCTCCACTACGGTACTCTCGTTATTGACGGTGTTACTCCTAACATGATTGACTACTTTGGTCGCCCTTACGAGGGTATTGCTGTTTTTGACGGTAAGAAGATTACTGTTACTGGTACTCTCTGGAACGGTAACAAGATTATTGACGAGCGCGCGATTAACCCTGACGGTTCTCTCCTCTTTCGCGTTACTATTAACGGTGTTACTGGTTGGCGCCTCTGTGAGCGCATTGCGGCTTAAAAGCTTGGCGTAATCATGGTCATAGCTGTTTCCTGTGTGAAATTGTTATCCGCTCACAATTCCACACAACATACGAGCCGG
GAL_21 C *GAL gene	GGCGATTAAGTTGGGTAACGCCAGGGTTTTCCCAGTCACGACGTTGTAAAACGACGGCCAGTGAATTCTAATACGACTCACTATAGGGGAATTGTGAGCGGATAACAATTCCCCTCTAGAAATAATTTTGTTTAACTTTAAGAAGGAGATATACATATGACTATGATTACTGACTCTCTCGCTGTTGTTCTCCAGCGCCGCGACTGGGAGAACCCTGGTGTTACTCAGCTCAACCGCCTCGCTGCTCACCCTCCTTTTGCTTCTTGGCGCAACTCTGAGGAGGCTCGCACTGACCGCCCTTCTCAGCAGCTCCGCTCTCTCAACGGTGAGTGGCGCTTTGCTTGGTTTCCTGCTCCTGAGGCTGTTCCTGAGTCTTGGCTCGAGTGTGACCTCCCTGAGGCTGACACTGTTGTTGTTCCTTCTAACTGGCAGATGCACGGTTACGACGCTCCTATTTACACTAACGTTACTTACCCTATTACTGTTAACCCTCCTTTTGTTCCTACTGAGAACCCTACTGGTTGTTACTCTCTCACTTTTAACGTTGACGAGTCTTGGCTCCAGGAGGGTCAGACTCGCATTATTTTTGACGGTGTTAACTCTGCTTTTCACCTCTGGTGTAACGGTCGCTGGGTTGGTTACGGTCAGGACTCTCGCCTCCCTTCTGAGTTTGACCTCTCTGCTTTTCTCCGCGCTGGTGAGAACCGCCTCGCTGTTATGGTTCTCCGCTGGTCTGACGGTTCTTACCTCGAGGACCAGGACATGTGGCGCATGTCTGGTATTTTTCGCGACGTTTCTCTCCTCCACAAGCCTACTACTCAGATTTCTGACTTTCACGTTGCTACTCGCTTTAACGACGACTTTTCTCGCGCTGTTCTCGAGGCTGAGGTTCAGATGTGTGGTGAGCTCCGCGACTACCTCCGCGTTACTGTTTCTCTCTGGCAGGGTGAGACTCAGGTTGCTTCTGGTACTGCTCCTTTTGGTGGTGAGATTATTGACGAGCGCGGTGGTTACGCTGACCGCGTTACTCTCCGCCTCAACGTTGAGAACCCTAAGCTCTGGTCTGCTGAGATTCCTAACCTCTACCGCGCTGTTGTTGAGCTCCACACTGCTGACGGTACTCTCATTGAGGCTGAGGCTTGTGACGTTGGTTTTCGCGAGGTTCGCATTGAGAACGGTCTCCTCCTCCTCAACGGTAAGCCTCTCCTCATTCGCGGTGTTAACCGCCACGAGCACCACCCTCTCCACGGTCAGGTTATGGACGAGCAGACTATGGTTCAGGACATTCTCCTCATGAAGCAGAACAACTTTAACGCTGTTCGCTGTTCTCACTACCCTAACCACCCTCTCTGGTACACTCTCTGTGACCGCTACGGTCTCTACGTTGTTGACGAGGCTAACATTGAGACTCACGGTATGGTTCCTATGAACCGCCTCACTGACGACCCTCGCTGGCTCCCTGCTATGTCTGAGCGCGTTACTCGCATGGTTCAGCGCGACCGCAACCACCCTTCTGTTATTATTTGGTCTCTCGGTAACGAGTCTGGTCACGGTGCTAACCACGACGCTCTCTACCGCTGGATTAAGTCTGTTGACCCTTCTCGCCCTGTTCAGTACGAGGGTGGTGGTGCTGACACTACTGCTACTGACATTATTTGTCCTATGTACGCTCGCGTTGACGAGGACCAGCCTTTTCCTGCTGTTCCTAAGTGGTCTATTAAGAAGTGGCTCTCTCTCCCTGGTGAGACTCGCCCTCTCATTCTCTGTGAGTACGCTCACGCTATGGGTAACTCTCTCGGTGGTTTTGCTAAGTACTGGCAGGCTTTTCGCCAGTACCCTCGCCTCCAGGGTGGTTTTGTTTGGGACTGGGTTGACCAGTCTCTCATTAAGTACGACGAGAACGGTAACCCTTGGTCTGCTTACGGTGGTGACTTTGGTGACACTCCTAACGACCGCCAGTTTTGTATGAACGGTCTCGTTTTTGCTGACCGCACTCCTCACCCTGCTCTCACTGAGGCTAAGCACCAGCAGCAGTTTTTTCAGTTTCGCCTCTCTGGTCAGACTATTGAGGTTACTTCTGAGTACCTCTTTCGCCACTCTGACAACGAGCTCCTCCACTGGATGGTTGCTCTCGACGGTAAGCCTCTCGCTTCTGGTGAGGTTCCTCTCGACGTTGCTCCTCAGGGTAAGCAGCTCATTGAGCTCCCTGAGCTCCCTCAGCCTGAGTCTGCTGGTCAGCTCTGGCTCACTGTTCGCGTTGTTCAGCCTAACGCTACTGCTTGGTCTGAGGCTGGTCACATTTCTGCTTGGCAGCAGTGGCGCCTCGCTGAGAACCTCTCTGTTACTCTCCCTGCTGCTTCTCACGCTATTCCTCACCTCACTACTTCTGAGATGGACTTTTGTATTGAGCTCGGTAACAAGCGCTGGCAGTTTAACCGCCAGTCTGGTTTTCTCTCTCAGATGTGGATTGGTGACAAGAAGCAGCTCCTCACTCCTCTCCGCGACCAGTTTACTCGCGCTCCTCTCGACAACGACATTGGTGTTTCTGAGGCTACTCGCATTGACCCTAACGCTTGGGTTGAGCGCTGGAAGGCTGCTGGTCACTACCAGGCTGAGGCTGCTCTCCTCCAGTGTACTGCTGACACTCTCGCTGACGCTGTTCTCATTACTACTGCTCACGCTTGGCAGCACCAGGGTAAGACTCTCTTTATTTCTCGCAAGACTTACCGCATTGACGGTTCTGGTCAGATGGCTATTACTGTTGACGTTGAGGTTGCTTCTGACACTCCTCACCCTGCTCGCATTGGTCTCAACTGTCAGCTCGCTCAGGTTGCTGAGCGCGTTAACTGGCTCGGTCTCGGTCCTCAGGAGAACTACCCTGACCGCCTCACTGCTGCTTGTTTTGACCGCTGGGACCTCCCTCTCTCTGACATGTACACTCCTTACGTTTTTCCTTCTGAGAACGGTCTCCGCTGTGGTACTCGCGAGCTCAACTACGGTCCTCACCAGTGGCGCGGTGACTTTCAGTTTAACATTTCTCGCTACTCTCAGCAGCAGCTCATGGAGACTTCTCACCGCCACCTCCTCCACGCTGAGGAGGGTACTTGGCTCAACATTGACGGTTTTCACATGGGTATTGGTGGTGACGACTCTTGGTCTCCTTCTGTTTCTGCTGAGTTTCAGCTCTCTGCTGGTCGCTACCACTACCAGCTCGTTTGGTGTCAGAAGTAAAAAAGCTTGGCGTAATCATGGTCATAGCTGTTTCCTGTGTGAAATTGTTATCCGCTCACAATTCCACACAACATACGAGCCGG
Luciferase_21 C *Luciferase gene	GGCGATTAAGTTGGGTAACGCCAGGGTTTTTGTTTAACTTTAAGAAGGAGATATACATATGGAGGACGCTTCCCAGTCACGACGTTGTAAAACGACGGCCAGTGAATTCTAATACGACTCACTATAGGGGAATTGTGAGCGGATAACAATTCCCCTCTAGAAATAATTTTGTTTAACTTTAAGAAGGAGATATACATATGGAGGACGCTAAGAACATTAAGAAGGGTCCTGCTCCTTTTTACCCTCTCGAGGACGGTACTGCTGGTGAGCAGCTCCACAAGGCTATGAAGCGCTACGCTCTCGTTCCTGGTACTATTGCTTTTACTGACGCTCACATTGAGGTTAACATTACTTACGCTGAGTACTTTGAGATGTCTGTTCGCCTCGCTGAGGCTATGAAGCGCTACGGTCTCAACACTAACCACCGCATTGTTGTTTGTTCTGAGAACTCTCTCCAGTTTTTTATGCCTGTTCTCGGTGCTCTCTTTATTGGTGTTGCTGTTGCTCCTGCTAACGACATTTACAACGAGCGCGAGCTCCTCAACTCTATGAACATTTCTCAGCCTACTGTTGTTTTTGTTTCTAAGAAGGGTCTCCAGAAGATTCTCAACGTTCAGAAGAAGCTCCCTATTATTCAGAAGATTATTATTATGGACTCTAAGACTGACTACCAGGGTTTTCAGTCTATGTACACTTTTGTTACTTCTCACCTCCCTCCTGGTTTTAACGAGTACGACTTTGTTCCTGAGTCTTTTGACCGCGACAAGACTATTGCTCTCATTATGAACTCTTCTGGTTCTACTGGTCTCCCTAAGGGTGTTGCTCTCCCTCACCGCACTGCTTGTGTTCGCTTTTCTCACGCTCGCGACCCTATTTTTGGTAACCAGATTATTCCTGACACTGCTATTCTCTCTGTTGTTCCTTTTCACCACGGTTTTGGTATGTTTACTACTCTCGGTTACCTCATTTGTGGTTTTCGCGTTGTTCTCATGTACCGCTTTGAGGAGGAGCTCTTTCTCCGCTCTCTCCAGGACTACAAGATTCAGTCTGCTCTCCTCGTTCCTACTCTCTTTTCTTTTTTTGCTAAGTCTACTCTCATTGACAAGTACGACCTCTCTAACCTCCACGAGATTGCTTCTGGTGGTGCTCCTCTCTCTAAGGAGGTTGGTGAGGCTGTTGCTAAGCGCTTTCACCTCCCTGGTATTCGCCAGGGTTACGGTCTCACTGAGACTACTTCTGCTATTCTCATTACTCCTGAGGGTGACGACAAGCCTGGTGCTGTTGGTAAGGTTGTTCCTTTTTTTGAGGCTAAGGTTGTTGACCTCGACACTGGTAAGACTCTCGGTGTTAACCAGCGCGGTGAGCTCTGTGTTCGCGGTCCTATGATTATGTCTGGTTACGTTAACAACCCTGAGGCTACTAACGCTCTCATTGACAAGGACGGTTGGCTCCACTCTGGTGACATTGCTTACTGGGACGAGGACGAGCACTTTTTTATTGTTGACCGCCTCAAGTCTCTCATTAAGTACAAGGGTTACCAGGTTGCTCCTGCTGAGCTCGAGTCTATTCTCCTCCAGCACCCTAACATTTTTGACGCTGGTGTTGCTGGTCTCCCTGACGACGACGCTGGTGAGCTCCCTGCTGCTGTTGTTGTTCTCGAGCACGGTAAGACTATGACTGAGAAGGAGATTGTTGACTACGTTGCTTCTCAGGTTACTACTGCTAAGAAGCTCCGCGGTGGTGTTGTTTTTGTTGACGAGGTTCCTAAGGGTCTCACTGGTAAGCTCGACGCTCGCAAGATTCGCGAGATTCTCATTAAGGCTAAGAAGGGTGGTAAGTCTAAGCTCTAAAAAAGCTTGGCGTAATCATGGTCATAGCTGTTTCCTGTGTGAAATTGTTATCCGCTCACAATTCCACACAACATACGAGCCGG
mStayGold_21 C ***mStayGold gene**	GGCGATTAAGTTGGGTAACGCCAGGGTTTTCCCAGTCACGACGTTGTAAAACGACGGCCAGTGAATTCTAATACGACTCACTATAGGGGAATTGTGAGCGGATAACAATTCCCCTCTAGAAATAATTTTGTTTAACTTTAAGAAGGAGATATACAT**ATGGTTTCTACTGGTGAGGAGCTCTTTACTGGTGTTGTTCCTTTTAAGTTTCAGCTCAAGGGTACTATTAACGGTAAGTCTTTTACTGTTGAGGGTGAGGGTGAGGGTAACTCTCACGAGGGTTCTCACAAGGGTAAGTACGTTTGTACTTCTGGTAAGCTCCCTATGTCTTGGGCTGCTCTCGGTACTTCTTTTGGTTACGGTATGAAGTACTACACTAAGTACCCTTCTGGTCTCAAGAACTGGTTTCACGAGGTTATGCCTGAGGGTTTTACTTACGACCGCCACATTCAGTACAAGGGTGACGGTTCTATTCACGCTAAGCACCAGCACTTTATGAAGAACGGTACTTACCACAACATTGTTGAGTTTACTGGTCAGGACTTTAAGGAGAACTCTCCTGTTCTCACTGGTGACATGGACGTTTCTCTCCCTAACGAGGTTCAGCACATTCCTATTGACGACGGTGTTGAGTGTACTGTTACTCTCCAGTACCCTCTCCTCTCTGACGAGTCTAAGTGTGTTGAGGCTTACCAGAACACTATTATTAAGCCTCTCCACAACCAGCCTGCTCCTGACGTTCCTTTTCACTGGATTCGCAAGCAGTACACTCAGTCTAAGGACGACACTGAGGAGCGCGACCACATTATTCAGTCTGAGACTCTCGAGGCTCACCTC**TAAAAAAGCTTGGCGTAATCATGGTCATAGCTGTTTCCTGTGTGAAATTGTTATCCGCTCACAATTCCACACAACATACGAGCCGG

**Appendix 1—table 2.** Primers used in this study.

Primer no.	Sequence
1	GGCGATTAAGTTGGGTAACGCCAG
2	CCGGCTCGTATGTTGTGTGG
3	GGTTCTGGTGGTAACTCTGGTTCTTCTGGTGGTTCTTCTGGTGTTTCTGGTTGGC
4	GGCCGCAAGCCTATTAAGAAATCTTCTTAAAGAGGCGCCAACCAGAAACACCAGAAG
5	GAACCAGAGTTACCACCAGAACCCTTCTGACACCAAACGAGCTGG
6	GAACCAGAGTTACCACCAGAACCGAGCTTAGACTTACCACCCTTCTTAGC
7	GAACCAGAGTTACCACCAGAACCGAGGTGAGCCTCGAGAGTCTC
8	GGCCGCAAGCCTATTAAGAAATC
9	CCGCGTAATACGACTCACTATAGGGGCTATAGCTCAGCTG
10	TGGTGGAGCTAAGCGGGATCG
11	ATGCAAGCGCTCTCCCAGC
12	GGAGAGCGCTTGCATCGCATGCAAGAGGTCAG
13	CCGCGTAATACGACTCACTATAGCGCCCGTAGCTCAG
14	TGGCGCGCCCGACAGGATTCG
15	AGGGCAGCGCTCTATCCAGCTGAG
16	ATAGAGCGCTGCCCTGCGGAGGCAGAGG
17	ATAGAGCGCTGCCCTCCTGAGGCAGAGGTC
18	CCGCGTAATACGACTCACTATAGGAGCGGTAGTTCAGTCG
19	TGGCGGAACGGACGGGACTCG
20	CCGCGTAATACGACTCACTATAGGCGCGTTAACAAAGCG
21	TGGAGGCGCGTTCCGGAGTCG
22	CCGCGTAATACGACTCACTATAGCGGGAATAGCTCAGTTG
23	TGGAGCGGGAAACGAGAC
24	AAGGTCGTGCTCTACCAACTGAGCTATTC
25	GTAGAGCACGACCTTCCCAAGGTCGGGGTC
26	CCGCGTAATACGACTCACTATAGTCCCCTTCGTCTAGAGG
27	TGGCGTCCCCTAGGGGATTCG
28	CCGCGTAATACGACTCACTATAGGTGGCTATAGCTCAGTTG
29	TGGGGTGGCTAATGGGATTCG
30	CCGCGTAATACGACTCACTATAGCGAAGGTGGCGGAATTG
31	TGGTGCGAGGGGGGGGA
32	AAGCTAGCGCGTCTACCAATTCCGC
33	TAGACGCGCTAGCTTCAAGTGTTAGTGTCCTTAC
34	TAGACGCGCTAGCTTGAGGTGTTAGTGTCCTTAC
35	CCGCGTAATACGACTCACTATAGGGTCGTTAGCTCAGTTGG
36	TGGTGGGTCGTGCAGGATT
37	CCGCGTAATACGACTCACTATAGGCTACGTAGCTCAGTTG
38	TGGTGGCTACGACGGGATTCG
39	CCGCGTAATACGACTCACTATAGCCCGGATAGCTCAGTC
40	TGGTGCCCGGACTCGGAA
41	CCGCGTAATACGACTCACTATAGGTGAGGTGTCCGAGTG
42	TGGCGGTGAGGGGGGGATTCG
43	AGGCGTGCTCCTTCAGCCACTCG
44	TGAAGGAGCACGCCTCGAAAGTGTGTATACGGCAAC
45	TGAAGGAGCACGCCTGCTAAGTGTGTATACGGCAAC
46	CCGCGTAATACGACTCACTATAGCTGATATGGCTCAGTTGG
47	TGGTGCTGATACCCAGAGTCG
48	AAGGGTGCGCTCTACCAACTGAGC
49	GTAGAGCGCACCCTTCGTAAGGGTGAGGTCCCCAG
50	CCGCGTAATACGACTCACTATAGGTGGGGTTCCCGAG
51	TGGTGGTGGGGGAAGGATTCG
52	CCGCGTAATACGACTCACTATAGCGTCCGTAGCTCAGTTG
53	TGGTGCGTCCGAGTGGACTCG
54	AAGGTGGTGCTCTAACCAACTGAGCTAC
55	TTAGAGCACCACCTTCACATGGTGGGGGTCGG
56	GAGATTAATACGACTCACTATAGGGCACGTAGCGCAGCCTGG
57	TGGTCGGCACGAGAGGATTT
58	ATGACGGTGCGCTACCAGGCTGC
59	GTAGCGCACCGTCATCGGGTGTCGGGGG
60	GGAGAGCGCTTGCATCAAATGCAAGAGGTCAG
61	GGAGAGCGCTTGCATCGAATGCAAGAGGTCAG
62	GGAGAGCGCTTGCATCAGATGCAAGAGGTCAG
63	GGAGAGCGCTTGCATCGGATGCAAGAGGTCAG
64	GGAGAGCGCTTGCATCCGATGCAAGAGGTCAG
65	GGAGAGCGCTTGCATGCTATGCAAGAGGTCAG
66	GGAGAGCGCTTGCATCGTATGCAAGAGGTCAG
67	GGAGAGCGCTTGCATCACATGCAAGAGGTCAG
68	GGAGAGCGCTTGCATCCCATGCAAGAGGTCAG
69	TGAAGGAGCACGCCTCAAAAGTGTGTATACGGCAAC
70	TGAAGGAGCACGCCTCAGAAGTGTGTATACGGCAAC
71	TGAAGGAGCACGCCTCGGAAGTGTGTATACGGCAAC
72	TGAAGGAGCACGCCTCCGAAGTGTGTATACGGCAAC
73	TGAAGGAGCACGCCTCGTAAGTGTGTATACGGCAAC
74	TGAAGGAGCACGCCTCACAAGTGTGTATACGGCAAC
75	TGAAGGAGCACGCCTCGCAAGTGTGTATACGGCAAC
76	TGAAGGAGCACGCCTCCCAAGTGTGTATACGGCAAC
77	TAGACGCGCTAGCTTCGAGTGTTAGTGTCCTTAC
78	TAGACGCGCTAGCTTCGGGTGTTAGTGTCCTTAC
79	TAGACGCGCTAGCTTCCGGTGTTAGTGTCCTTAC
80	TAGACGCGCTAGCTTGCTGTGTTAGTGTCCTTAC
81	TAGACGCGCTAGCTTCGTGTGTTAGTGTCCTTAC
82	TAGACGCGCTAGCTTCACGTGTTAGTGTCCTTAC
83	TAGACGCGCTAGCTTCGCGTGTTAGTGTCCTTAC
84	TAGACGCGCTAGCTTCCCGTGTTAGTGTCCTTAC
85	GGAGAGCGCTTGCATUGCATGCAAGAGGTCAG
86	ATAGAGCGCTGCCCTTCGGAGGCAGAGG
87	ATAGAGCGCTGCCCTTCTGAGGCAGAGGTC
88	GAGATTAATACGACTCACTATAGGGGGTATCGCCAAGCGGTAAG
89	TGGCGGGGGTACGAGGATTCG
90	AATCCGGTGCCTTACCGCTTG
91	GTAAGGCACCGGATTTTGATTCCGGCATTCC
92	AGGGCGGTGTCCTGGGCCTC
93	CCAGGACACCGCCCTTTCACGGCGGTAAC
94	GTAGAGCACGACCTTTCCAAGGTCGGGGTC
95	GAGATTAATACGACTCACTATAGGGCTTGTAGCTCAGGTGGTTAG
96	TGGTAGGCCTGAGTGGACTTG
97	AGGGGTGCGCTCTAACCACC
98	TTAGAGCGCACCCCTTATAAGGGTGAGGTCG
99	TAGACGCGCTAGCTTTAAGTGTTAGTGTCCTTACG
100	TAGACGCGCTAGCTTTAGGTGTTAGTGTCCTTACG
101	AGTCAACTGCTCTACCAACTGAGC
102	GTAGAGCAGTTGACTTTTAATCAATTGGTCGC
103	GTAGCGCACCGTCATTGGGTGTCGGGGG
104	TGAAGGAGCACGCCTTGAAAGTGTGTATACGGCAAC
105	GTAGAGCGCACCCTTTGTAAGGGTGAGGTCCCCAG
106	TTAGAGCACCACCTTTACATGGTGGGGGTCGG
107	ACACTCTTTCCCTACACGACGCTCTTCCGATCTCGTAACCTGCTTCTTGGCGCAACTCTG
108	ACACTCTTTCCCTACACGACGCTCTTCCGATCTGAATTGGGGCTTCTTGGCGCAACTCTG
109	ACACTCTTTCCCTACACGACGCTCTTCCGATCTAACCACTCGCTTCTTGGCGCAACTCTG
110	ACACTCTTTCCCTACACGACGCTCTTCCGATCTCAGGGTATGCTTCTTGGCGCAACTCTG
111	ACACTCTTTCCCTACACGACGCTCTTCCGATCTTAGGAGCAGCTTCTTGGCGCAACTCTG
112	GTGACTGGAGTTCAGACGTGTGCTCTTCCGATCTGAGACTTCGGTCCTCGAGGTAAGAACCGTC
113	ACACTCTTTCCCTACACGACGCTCTTCCGATCTATCACTGCTTACAACGAGCGCGAGCTC
114	ACACTCTTTCCCTACACGACGCTCTTCCGATCTGAAGGCAATTACAACGAGCGCGAGCTC
115	ACACTCTTTCCCTACACGACGCTCTTCCGATCTCTCGAGAATTACAACGAGCGCGAGCTC
116	ACACTCTTTCCCTACACGACGCTCTTCCGATCTCGTCAACATTACAACGAGCGCGAGCTC
117	ACACTCTTTCCCTACACGACGCTCTTCCGATCTCAAGAGTCTTACAACGAGCGCGAGCTC
118	GTGACTGGAGTTCAGACGTGTGCTCTTCCGATCTAGGGTGAGCGGAGAAAGAGCTCCTCC
119	ACACTCTTTCCCTACACGACGCTCTTCCGATCTGGACTAGTGTTGAGGGTGAGGGTGAGG
120	ACACTCTTTCCCTACACGACGCTCTTCCGATCTCAGAGGAAGTTGAGGGTGAGGGTGAGG
121	ACACTCTTTCCCTACACGACGCTCTTCCGATCTCGCTTGATGTTGAGGGTGAGGGTGAGG
122	ACACTCTTTCCCTACACGACGCTCTTCCGATCTGGAAATCGGTTGAGGGTGAGGGTGAGG
123	ACACTCTTTCCCTACACGACGCTCTTCCGATCTAGTGCTCTGTTGAGGGTGAGGGTGAGG
124	GTGACTGGAGTTCAGACGTGTGCTCTTCCGATCTTCGATCTGGCGAATCCAGTGAAAAGGAACGTC

**Appendix 1—table 3.** tRNAs used in this study.

tRNA(or pre-tRNA)	Primer set for IVT template	Primer set for mutation	Sequence
tRNA^Ala^GGC	9, 10	-	GGGGCUAUAGCUCAGCUGGGAGAGCGCUUGCAUGGCAUGCAAGAGGUCAGCGGUUCGAUCCCGCUUAGCUCCACCA
tRNA^Ala^CGC	9, 10	11, 12	GGGGCUAUAGCUCAGCUGGGAGAGCGCUUGCAUCGCAUGCAAGAGGUCAGCGGUUCGAUCCCGCUUAGCUCCACCA
tRNA^Arg^CCG	13, 14	-	GCGCCCGUAGCUCAGCUGGAUAGAGCGCUGCCCUCCGGAGGCAGAGGUCUCAGGUUCGAAUCCUGUCGGGCGCGCCA
tRNA^Arg^GCG	13, 14	15, 16	GCGCCCGUAGCUCAGCUGGAUAGAGCGCUGCCCUGCGGAGGCAGAGGUCUCAGGUUCGAAUCCUGUCGGGCGCGCCA
tRNA^Arg^CCU	13, 14	15, 17	GCGCCCGUAGCUCAGCUGGAUAGAGCGCUGCCCUCCUGAGGCAGAGGUCUCAGGUUCGAAUCCUGUCGGGCGCGCCA
tRNA^Asn^GUC	-	-	UCCUCUGUAGUUCAGUCGGUAGAACGGCGGACUGUUAAUCCGUAUGUCACUGGUUCGAGUCCAGUCAGAGGAGCCA
tRNA^Asp^GUC	18, 19	-	GGAGCGGUAGUUCAGUCGGUUAGAAUACCUGCCUGUCACGCAGGGGGUCGCGGGUUCGAGUCCCGUCCGUUCCGCCA
tRNA^Cys^GCA	20, 21	-	GGCGCGUUAACAAAGCGGUUAUGUAGCGGAUUGCAAAUCCGUCUAGUCCGGUUCGACUCCGGAACGCGCCUCCA
tRNA^Gln^CUG	-	-	UGGGGUAUCGCCAAGCGGUAAGGCACCGGAUUCUGAUUCCGGCAUUCCGAGGUUCGAAUCCUCGUACCCCAGCCA
tRNA^Gly^GCC	22, 23	-	GCGGGAAUAGCUCAGUUGGUAGAGCACGACCUUGCCAAGGUCGGGGUCGCGAGUUCGAGUCUCGUUUCCCGCUCCA
tRNA^Gly^CCC	22, 23	24, 25	GCGGGAAUAGCUCAGUUGGUAGAGCACGACCUUCCCAAGGUCGGGGUCGCGAGUUCGAGUCUCGUUUCCCGCUCCA
tRNA^Glu^CUC	26, 27	-	GUCCCCUUCGUCUAGAGGCCCAGGACACCGCCCUCUCACGGCGGUAACAGGGGUUCGAAUCCCCUAGGGGACGCCA
tRNA^His^GUG	28, 29	-	GGUGGCUAUAGCUCAGUUGGUAGAGCCCUGGAUUGUGAUUCCAGUUGUCGUGGGUUCGAAUCCCAUUAGCCACCCCA
tRNA^Ile^GAU	-	-	AGGCUUGUAGCUCAGGUGGUUAGAGCGCACCCCUGAUAAGGGUGAGGUCGGUGGUUCAAGUCCACUCAGGCCUACCA
tRNA^Leu^CAG	30, 31	-	GCGAAGGUGGCGGAAUUGGUAGACGCGCUAGCUUCAGGUGUUAGUGUCCUUACGGACGUGGGGGUUCAAGUCCCCCCCCUCGCACCA
tRNA^Leu^CAA	30, 31	32, 33	GCGAAGGUGGCGGAAUUGGUAGACGCGCUAGCUUCAAGUGUUAGUGUCCUUACGGACGUGGGGGUUCAAGUCCCCCCCCUCGCACCA
tRNA^Leu^GAG	30, 31	32, 34	GCGAAGGUGGCGGAAUUGGUAGACGCGCUAGCUUGAGGUGUUAGUGUCCUUACGGACGUGGGGGUUCAAGUCCCCCCCCUCGCACCA
tRNA^Lys^CUU	35, 36	-	GGGUCGUUAGCUCAGUUGGUAGAGCAGUUGACUCUUAAUCAAUUGGUCGCAGGUUCGAAUCCUGCACGACCCACCA
tRNA^fMet^CAU	-	-	CGCGGGGUGGAGCAGCCUGGUAGCUCGUCGGGCUCAUAACCCGAAGAUCGUCGGUUCAAAUCCGGCCCCCGCAACCA
tRNA^mMet^CAU	37, 38	-	GGCUACGUAGCUCAGUUGGUUAGAGCACAUCACUCAUAAUGAUGGGGUCACAGGUUCGAAUCCCGUCGUAGCCACCA
tRNA^Phe^GAA	39, 40	-	GCCCGGAUAGCUCAGUCGGUAGAGCAGGGGAUUGAAAAUCCCCGUGUCCUUGGUUCGAUUCCGAGUCCGGGCACCA
tRNA^Ser^GGA	41, 42	-	GGUGAGGUGUCCGAGUGGCUGAAGGAGCACGCCUGGAAAGUGUGUAUACGGCAACGUAUCGGGGGUUCGAAUCCCCCCCUCACCGCCA
tRNA^Ser^CGA	41, 42	43, 44	GGUGAGGUGUCCGAGUGGCUGAAGGAGCACGCCUCGAAAGUGUGUAUACGGCAACGUAUCGGGGGUUCGAAUCCCCCCCUCACCGCCA
tRNA^Ser^GCU	41, 42	43, 45	GGUGAGGUGUCCGAGUGGCUGAAGGAGCACGCCUGCUAAGUGUGUAUACGGCAACGUAUCGGGGGUUCGAAUCCCCCCCUCACCGCCA
tRNA^Thr^GGU	46, 47	-	GCUGAUAUGGCUCAGUUGGUAGAGCGCACCCUUGGUAAGGGUGAGGUCCCCAGUUCGACUCUGGGUAUCAGCACCA
tRNA^Thr^CGU	46, 47	48, 49	GCUGAUAUGGCUCAGUUGGUAGAGCGCACCCUUCGUAAGGGUGAGGUCCCCAGUUCGACUCUGGGUAUCAGCACCA
tRNA^Trp^CCA	-	-	AGGGGCGUAGUUCAAUUGGUAGAGCACCGGUCUCCAAAACCGGGUGUUGGGAGUUCGAGUCUCUCCGCCCCUGCCA
tRNA^Tyr^GUA	50, 51	-	GGUGGGGUUCCCGAGCGGCCAAAGGGAGCAGACUGUAAAUCUGCCGUCACAGACUUCGAAGGUUCGAAUCCUUCCCCCACCACCA
tRNA^Val^GAC	52, 53	-	GCGUCCGUAGCUCAGUUGGUUAGAGCACCACCUUGACAUGGUGGGGGUCGGUGGUUCGAGUCCACUCGGACGCACCA
tRNA^Val^CAC	52, 53	54, 55	GCGUCCGUAGCUCAGUUGGUUAGAGCACCACCUUCACAUGGUGGGGGUCGGUGGUUCGAGUCCACUCGGACGCACCA
tRNA^Pro^GGG	-	-	CGGCACGUAGCGCAGCCUGGUAGCGCACCGUCAUGGGGUGUCGGGGGUCGGAGGUUCAAAUCCUCUCGUGCCGACCA
tRNA^Pro^CGG	56, 57	58, 59	GGGCACGUAGCGCAGCCUGGUAGCGCACCGUCAUCGGGUGUCGGGGGUCGGAGGUUCAAAUCCUCUCGUGCCGACCA
tRNA^Ala^CAA *anticodon	9, 10	11, 60	GGGGCUAUAGCUCAGCUGGGAGAGCGCUUGCAUCAAAUGCAAGAGGUCAGCGGUUCGAUCCCGCUUAGCUCCACCA
tRNA^Ala^CGA	9, 10	11, 61	GGGGCUAUAGCUCAGCUGGGAGAGCGCUUGCAUCGAAUGCAAGAGGUCAGCGGUUCGAUCCCGCUUAGCUCCACCA
tRNA^Ala^CAG	9, 10	11, 62	GGGGCUAUAGCUCAGCUGGGAGAGCGCUUGCAUCAGAUGCAAGAGGUCAGCGGUUCGAUCCCGCUUAGCUCCACCA
tRNA^Ala^CGG	9, 10	11, 63	GGGGCUAUAGCUCAGCUGGGAGAGCGCUUGCAUCGGAUGCAAGAGGUCAGCGGUUCGAUCCCGCUUAGCUCCACCA
tRNA^Ala^CCG	9, 10	11, 64	GGGGCUAUAGCUCAGCUGGGAGAGCGCUUGCAUCCGAUGCAAGAGGUCAGCGGUUCGAUCCCGCUUAGCUCCACCA
tRNA^Ala^GCU	9, 10	11, 65	GGGGCUAUAGCUCAGCUGGGAGAGCGCUUGCAUGCUAUGCAAGAGGUCAGCGGUUCGAUCCCGCUUAGCUCCACCA
tRNA^Ala^CGU	9, 10	11, 66	GGGGCUAUAGCUCAGCUGGGAGAGCGCUUGCAUCGUAUGCAAGAGGUCAGCGGUUCGAUCCCGCUUAGCUCCACCA
tRNA^Ala^CAC	9, 10	11, 67	GGGGCUAUAGCUCAGCUGGGAGAGCGCUUGCAUCACAUGCAAGAGGUCAGCGGUUCGAUCCCGCUUAGCUCCACCA
tRNA^Ala^CCC	9, 10	11, 68	GGGGCUAUAGCUCAGCUGGGAGAGCGCUUGCAUCCCAUGCAAGAGGUCAGCGGUUCGAUCCCGCUUAGCUCCACCA
tRNA^Ser^CAA *anticodon	41, 42	43, 69	GGUGAGGUGUCCGAGUGGCUGAAGGAGCACGCCUCAAAAGUGUGUAUACGGCAACGUAUCGGGGGUUCGAAUCCCCCCCUCACCGCCA
tRNA^Ser^CAG	41, 42	43, 70	GGUGAGGUGUCCGAGUGGCUGAAGGAGCACGCCUCAGAAGUGUGUAUACGGCAACGUAUCGGGGGUUCGAAUCCCCCCCUCACCGCCA
tRNA^Ser^CGG	41, 42	43, 71	GGUGAGGUGUCCGAGUGGCUGAAGGAGCACGCCUCGGAAGUGUGUAUACGGCAACGUAUCGGGGGUUCGAAUCCCCCCCUCACCGCCA
tRNA^Ser^CCG	41, 42	43, 72	GGUGAGGUGUCCGAGUGGCUGAAGGAGCACGCCUCCGAAGUGUGUAUACGGCAACGUAUCGGGGGUUCGAAUCCCCCCCUCACCGCCA
tRNA^Ser^CGU	41, 42	43, 73	GGUGAGGUGUCCGAGUGGCUGAAGGAGCACGCCUCGUAAGUGUGUAUACGGCAACGUAUCGGGGGUUCGAAUCCCCCCCUCACCGCCA
tRNA^Ser^CAC	41, 42	43, 74	GGUGAGGUGUCCGAGUGGCUGAAGGAGCACGCCUCACAAGUGUGUAUACGGCAACGUAUCGGGGGUUCGAAUCCCCCCCUCACCGCCA
tRNA^Ser^CGC	41, 42	43, 75	GGUGAGGUGUCCGAGUGGCUGAAGGAGCACGCCUCGCAAGUGUGUAUACGGCAACGUAUCGGGGGUUCGAAUCCCCCCCUCACCGCCA
tRNA^Ser^CCC	41, 42	43, 76	GGUGAGGUGUCCGAGUGGCUGAAGGAGCACGCCUCCCAAGUGUGUAUACGGCAACGUAUCGGGGGUUCGAAUCCCCCCCUCACCGCCA
tRNA^Leu^CGA *anticodon	30, 31	32, 77	GCGAAGGUGGCGGAAUUGGUAGACGCGCUAGCUUCGAGUGUUAGUGUCCUUACGGACGUGGGGGUUCAAGUCCCCCCCCUCGCACCA
tRNA^Leu^CGG	30, 31	32, 78	GCGAAGGUGGCGGAAUUGGUAGACGCGCUAGCUUCGGGUGUUAGUGUCCUUACGGACGUGGGGGUUCAAGUCCCCCCCCUCGCACCA
tRNA^Leu^CCG	30, 31	32, 79	GCGAAGGUGGCGGAAUUGGUAGACGCGCUAGCUUCCGGUGUUAGUGUCCUUACGGACGUGGGGGUUCAAGUCCCCCCCCUCGCACCA
tRNA^Leu^GCU	30, 31	32, 80	GCGAAGGUGGCGGAAUUGGUAGACGCGCUAGCUUGCUGUGUUAGUGUCCUUACGGACGUGGGGGUUCAAGUCCCCCCCCUCGCACCA
tRNA^Leu^CGU	30, 31	32, 81	GCGAAGGUGGCGGAAUUGGUAGACGCGCUAGCUUCGUGUGUUAGUGUCCUUACGGACGUGGGGGUUCAAGUCCCCCCCCUCGCACCA
tRNA^Leu^CAC	30, 31	32, 82	GCGAAGGUGGCGGAAUUGGUAGACGCGCUAGCUUCACGUGUUAGUGUCCUUACGGACGUGGGGGUUCAAGUCCCCCCCCUCGCACCA
tRNA^Leu^CGC	30, 31	32, 83	GCGAAGGUGGCGGAAUUGGUAGACGCGCUAGCUUCGCGUGUUAGUGUCCUUACGGACGUGGGGGUUCAAGUCCCCCCCCUCGCACCA
tRNA^Leu^CCC	30, 31	32, 84	GCGAAGGUGGCGGAAUUGGUAGACGCGCUAGCUUCCCGUGUUAGUGUCCUUACGGACGUGGGGGUUCAAGUCCCCCCCCUCGCACCA
tRNA^Ala^UGC	9, 10	11, 85	GGGGCUAUAGCUCAGCUGGGAGAGCGCUUGCAUUGCAUGCAAGAGGUCAGCGGUUCGAUCCCGCUUAGCUCCACCA
tRNA^Arg^UCG	13, 14	15, 86	GCGCCCGTAGCTCAGCTGGATAGAGCGCTGCCCTUCGGAGGCAGAGGTCTCAGGTTCGAATCCTGTCGGGCGCGCCA
tRNA^Arg^UCU	13, 14	15, 87	GCGCCCGTAGCTCAGCTGGATAGAGCGCTGCCCTUCUGAGGCAGAGGTCTCAGGTTCGAATCCTGTCGGGCGCGCCA
tRNA^Gln^UUG	88, 89	90, 91	TGGGGTATCGCCAAGCGGTAAGGCACCGGATTTTGATTCCGGCATTCCGAGGTTCGAATCCTCGTACCCCAGCCA
tRNA^Glu^UUC	26, 27	92, 93	GTCCCCTTCGTCTAGAGGCCCAGGACACCGCCCTTTCACGGCGGTAACAGGGGTTCGAATCCCCTAGGGGACGCCA
tRNA^Gly^UCC	22, 23	24, 94	GCGGGAATAGCTCAGTTGGTAGAGCACGACCTTUCCAAGGTCGGGGTCGCGAGTTCGAGTCTCGTTTCCCGCTCCA
tRNA^Ile^UAU	95, 96	97, 98	AGGCTTGTAGCTCAGGTGGTTAGAGCGCACCCCTTATAAGGGTGAGGTCGGTGGTTCAAGTCCACTCAGGCCTACCA
tRNA^Leu^UAA	30, 31	32, 99	GCGAAGGUGGCGGAAUUGGUAGACGCGCUAGCUUUAAGUGUUAGUGUCCUUACGGACGUGGGGGUUCAAGUCCCCCCCCUCGCACCA
tRNA^Leu^UAG	30, 31	32, 100	GCGAAGGUGGCGGAAUUGGUAGACGCGCUAGCUUUAGGUGUUAGUGUCCUUACGGACGUGGGGGUUCAAGUCCCCCCCCUCGCACCA
tRNA^Lys^UUU	35, 36	101, 102	GGGTCGTTAGCTCAGTTGGTAGAGCAGTTGACTTTTAATCAATTGGTCGCAGGTTCGAATCCTGCACGACCCACCA
tRNA^Pro^UGG	56, 57	58, 103	CGGCACGTAGCGCAGCCTGGTAGCGCACCGTCATUGGGTGTCGGGGGTCGGAGGTTCAAATCCTCTCGTGCCGACCA
tRNA^Ser^UGA	41, 42	43, 104	GGUGAGGUGUCCGAGUGGCUGAAGGAGCACGCCUUGAAAGUGUGUAUACGGCAACGUAUCGGGGGUUCGAAUCCCCCCCUCACCGCCA
tRNA^Thr^UGU	46, 47	48, 105	GCTGATATGGCTCAGTTGGTAGAGCGCACCCTTUGUAAGGGTGAGGTCCCCAGTTCGACTCTGGGTATCAGCACCA
tRNA^Val^UAC	52, 53	54, 106	GCGTCCGTAGCTCAGTTGGTTAGAGCACCACCTTUACATGGTGGGGGTCGGTGGTTCGAGTCCACTCGGACGCACCA

**Appendix 1—table 4.** Weights for nucleotide substitution probabilities.

	First base	Second base	Third base
For transitions	1	0.5	1
For transversions	0.5	0.1	1

**Appendix 1—table 5.** Physicochemical property values of amino acids used for cost evaluation.

Amino acid	Polar requirement (PR)	Molecular volume (MV)	Hydropathy index (HI)
Ala	7	31	1.8
Arg	9.1	124	–4.5
Asp	13	54	–3.5
Asn	10	56	–3.5
Cys	4.8	55	2.5
Glu	12.5	83	–3.5
Gln	8.6	85	–3.5
Gly	7.9	3	–0.4
His	8.4	96	–3.2
Ile	4.9	111	4.5
Leu	4.9	111	3.8
Lys	10.1	119	–3.9
Met	5.3	105	1.9
Phe	5	132	2.8
Pro	6.6	32.5	–1.6
Ser	7.5	32	–0.8
Thr	6.6	61	–0.7
Trp	5.2	170	–0.9
Tyr	5.4	136	–1.3
Val	5.6	84	4.2

**Appendix 1—table 6.** Standard composition of the tRNA-free PURE system (tfPURE).

Component	Concentration	Component	Concentration
Initiation Factor 1	25 µM	Tryptophanyl-tRNA Synthetase	28 nM
Initiation Factor 2	1.0 µM	Tyrosyl-tRNA Synthetase	0.15 µM
Initiation Factor 3	4.9 µM	Valyl-tRNA Synthetase	17 nM
Elongation Factor G	1.1 µM	Methionyl-tRNA Formyltransferase	0.59 µM
Elongation Factor Tu	80 µM	Myokinase	1.4 µM
Elongation Factor Ts	3.3 µM	Creatine kinase	0.25 µM
Release Factor 1	49 nM	Nucleoside diphosphate kinase	16 nM
Release Factor 2	48 nM	Pyrophosphatase	41 nM
Release Factor 3	0.17 µM	Trigger Factor	1.0 µM
Ribosome Recycling Factor	3.9 µM	coli DEAH type RNA helicase A	63 nM
Alanyl-tRNA Synthetase	0.73 µM	Ribosome	1.0 µM
Arginyl-tRNA Synthetase	31 nM	Tyrosine	0.30 mM
Asparaginyl-tRNA Synthetase	0.42 µM	Cysteine	0.30 mM
Asparagyl-tRNA Synthetase	0.12 µM	18 other amino acids	0.36 mM
Cysteinyl-tRNA Synthetase	24 nM	ATP	0.38 mM
Glutaminyl-tRNA Synthetase	60 nM	GTP	0.25 mM
Glutamyl-tRNA Synthetase	0.23 µM	CTP	0.13 mM
Glycyl-tRNA Synthetase	86 nM	UTP	0.13 mM
Histidyl-tRNA Synthetase	85 nM	N-2-hydroxyethylpiperazine-N'–2-ethanesulfonic acid (pH7.6)	0.10 M
Isoleucyl-tRNA Synthetase	0.37 µM	Glutamic acid potassium salt	70 mM
Leucyl-tRNA Synthetase	41 nM	Spermidine	0.375 mM
Lysyl-tRNA Synthetase	0.12 µM	Creatine phosphate	25 mM
Methionyl-tRNA Synthetase	0.11 µM	Dithiothreitol	6 mM
Phenylalanyl-tRNA Synthetase	0.13 µM	10-formyl-5,6,7,8-tetrahydro folic acid	10 µg/mL
Prolyl-tRNA Synthetase	0.17 µM	Yeast inorganic pyrophosphatase (NEB)	0.2 units/mL
Seryl-tRNA Synthetase	78 nM	RNase Plus Inhibitor (Promega)	0.1 U/µL
Threonyl-tRNA Synthetase	84 nM	T7 RNAP (Takara)	1.7 U/µL

**Appendix 1—table 7.** tRNA composition in each genetic code.

tRNA (ng/ µL)	MGC	near-SGC(RV)	SGC	Code1	Code2	Code3	Code4	Code5	Code6	Code7	Code8	Code9	Code10
tRNA^Ala^GGC	12	12	12	12	12	12	12	12	12	12	12	12	12
tRNA^Ala^CGC	-	12	12	12	-	12	-	12	-	-	-	-	12
tRNA^Arg^CCG	-	12	12	-	-	-	-	-	-	-	-	-	-
tRNA^Arg^GCG	12	12	12	12	12	12	12	12	12	12	12	12	12
tRNA^Arg^CCU	-	100	100	12	12	12	12	12	12	12	12	12	12
tRNA^Asp^GUC	12	12	12	12	12	12	12	12	12	12	12	12	12
tRNA^Cys^GCA	12	12	12	12	12	12	12	12	12	12	12	12	12
tRNA^Gly^GCG	12	12	12	12	12	12	12	12	12	12	12	12	12
tRNA^Gly^CCC	-	12	12	12	12	12	12	12	12	12	12	12	12
tRNA^Glu^CUC	100	100	100	100	100	100	100	100	100	100	100	100	100
tRNA^His^GUG	12	12	12	12	12	12	12	12	12	12	12	12	12
tRNA^Leu^CAG	-	12	12	-	-	-	-	-	-	12	-	12	-
tRNA^Leu^CAA	-	12	12	-	-	12	-	12	-	12	-	12	-
tRNA^Leu^GAG	12	12	12	12	12	12	12	12	12	12	12	12	12
tRNA^Lys^CUU	12	12	12	12	12	12	12	12	12	12	12	12	12
tRNA^mMet^CAU	12	12	12	12	12	12	12	12	12	12	12	12	12
tRNA^Phe^GAA	12	12	12	12	12	12	12	12	12	12	12	12	12
tRNA^Ser^GGA	12	12	12	12	12	12	12	12	12	12	12	12	12
tRNA^Ser^CGA	-	12	12	-	-	-	-	-	-	-	-	12	12
tRNA^Ser^GCU	-	12	12	-	-	-	12	-	-	-	-	-	12
tRNA^Thr^GGU	12	12	12	12	12	12	12	12	12	12	12	12	12
tRNA^Thr^CGU	-	12	12	-	-	-	-	-	-	-	-	-	-
tRNA^Tyr^GUA	12	12	12	12	12	12	12	12	12	12	12	12	12
tRNA^Val^GAC	12	12	12	12	12	12	12	12	12	12	12	12	12
tRNA^Val^CAC	-	100	100	-	-	-	-	-	-	-	-	-	-
tRNA^Pro^CGG	-	100	100	-	-	-	-	-	-	-	-	-	-
tRNA^Pro^GGG	100	100	100	100	100	100	100	100	100	100	100	100	100
tRNA^Ile^GAU	100	100	100	100	100	100	100	100	100	100	100	100	100
tRNA^Asn^GUU	100	100	100	100	100	100	100	100	100	100	100	100	100
tRNA^Gln^CUG	12	12	12	12	12	12	12	12	12	12	12	12	12
tRNA^Trp^CCA	12	12	12	12	12	12	12	12	12	12	12	12	12
tRNA^fMet^CAU	12	12	12	12	12	12	12	12	12	12	12	12	12
tRNA^Ala^UGC	-	-	12	-	-	-	-	-	-	-	-	-	-
tRNA^Arg^UCG	-	-	12	-	-	-	-	-	-	-	-	-	-
tRNA^Arg^UCU	-	-	12	-	-	-	-	-	-	-	-	-	-
tRNA^Gln^UUG	-	-	12	-	-	-	-	-	-	-	-	-	-
tRNA^Glu^UUC	-	-	100	-	-	-	-	-	-	-	-	-	-
tRNA^Gly^UCC	-	-	12	-	-	-	-	-	-	-	-	-	-
tRNA^Ile^UAU	-	-	100	-	-	-	-	-	-	-	-	-	-
tRNA^Leu^UAA	-	-	12	-	-	-	-	-	-	-	-	-	-
tRNA^Leu^UAG	-	-	12	-	-	-	-	-	-	-	-	-	-
tRNA^Lys^UUU	-	-	12	-	-	-	-	-	-	-	-	-	-
tRNA^Pro^UGG	-	-	100	-	-	-	-	-	-	-	-	-	-
tRNA^Ser^UGA	-	-	12	-	-	-	-	-	-	-	-	-	-
tRNA^Thr^UGU	-	-	12	-	-	-	-	-	-	-	-	-	-
tRNA^Val^UAC	-	-	12	-	-	-	-	-	-	-	-	-	-
tRNA^Ala^CAA	-	-	-	40	-	-	40	-	-	-	40	-	40
tRNA^Ala^CGA	-	-	-	-	60	-	60	-	-	60	-	-	-
tRNA^Ala^CAG	-	-	-	12	-	12	-	12	-	-	12	-	12
tRNA^Ala^CGG	-	-	-	12	-	-	-	-	-	12	-	-	-
tRNA^Ala^CCG	-	-	-	-	-	-	12	-	-	-	12	-	-
tRNA^Ala^GCU	-	-	-	-	-	-	-	12	-	12	12	-	-
tRNA^Ala^CGU	-	-	-	60	-	-	-	-	-	-	-	-	-
tRNA^Ala^CAC	-	-	-	-	-	-	12	-	-	-	12	-	12
tRNA^Ser^CAA	-	-	-	-	12	-	-	-	12	-	-	-	-
tRNA^Ser^CAG	-	-	-	-	12	-	12	-	12	-	-	-	-
tRNA^Ser^CGG	-	-	-	-	12	12	-	12	-	-	-	12	12
tRNA^Ser^CCG	-	-	-	-	-	-	-	12	-	12	-	-	12
tRNA^Ser^CGU	-	-	-	-	80	-	80	80	-	80	-	80	80
tRNA^Ser^CAC	-	-	-	-	-	-	-	12	12	-	-	-	-
tRNA^Ser^CGC	-	-	-	-	-	-	-	-	-	12	-	12	-
tRNA^Leu^CGA	-	-	-	80	-	80	-	80	80	-	80	-	-
tRNA^Leu^CGG	-	-	-	-	-	-	12	-	12	-	12	12	-
tRNA^Leu^CCG	-	-	-	12	12	12	-	-	12	-	-	12	-
tRNA^Leu^GCU	-	-	-	80	80	80	-	-	80	-	-	-	-
tRNA^Leu^CGU	-	-	-	-	-	12	-	-	12	-	12	-	-
tRNA^Leu^CAC	-	-	-	12	12	12	-	-	-	12	-	12	-
tRNA^Leu^CGC	-	-	-	-	12	-	12	-	12	-	12	-	-

­

**Appendix 1—table 8.** Major statistical comparisons of NanoLuc translation efficiency in Figure 1D.

Comparison	p value (Tukey’s HSD test)
MGC vs near-SGC	2.45×10^–6^
near-SGC vs near-SGC (RV)	1.04×10^–6^
MGC vs near-SGC (RV)	0.9503
SGC vs near-SGC (RV)	2.44×10^–15^

**Appendix 1—table 9.** Welch’s t-test for NanoLuc translation using variant tRNAs in Figure 2.

Ala									
Codon	UUG	UCG	CUG	CCG	CGG	ACG	AGC	GUG	GGG
p value	1.12×10^–3^	1.98×10^–3^	1.15×10^–3^	1.36×10^–3^	1.49×10^–3^	1.44×10^–3^	1.94×10^–3^	1.50×10^–3^	1.80×10^–3^
Ser									
Codon	UUG	CUG	CCG	CGG	ACG	GUG	GCG	GGG	
p value	1.98×10^–3^	2.26×10^–3^	1.90×10^–4^	3.37×10^–2^	5.14×10^–4^	1.11×10^–2^	9.08×10^–4^	0.778	
Leu									
Codon	UCG	CCG	CGG	AGC	ACG	GUG	GCG	GGG	
p value	3.17×10^–4^	1.06×10^–5^	3.58×10^–4^	6.19×10^–5^	9.88×10^–4^	1.11×10^–3^	3.21×10^–4^	2.07×10^–4^	

**Appendix 1—table 10.** Spearman correlation analysis for Figure 5.

Reporter	Cost metric	Spearman’s ρ	p Value
GAL	Cost_PR	0.109	0.75
GAL	Cost_MV	0.100	0.770
GAL	Cost_HI	0.00	1.00
Luc	Cost_PR	–0.036	0.915
Luc	Cost_MV	0.245	0.467
Luc	Cost_HI	–0.055	0.873
mSG	Cost_PR	–0.191	0.574
mSG	Cost_MV	–0.018	0.958
mSG	Cost_HI	–0.227	0.502
