# Supplementary material for: Experimental verification of the error minimization theory using non-standard genetic codes constructed in vitro
Source: eLife. 2026 Jul 13;15:RP111164. doi: 10.7554/eLife.111164 (PMC13363220; doi:10.7554/eLife.111164)
Supplement: Peer review history

# Editor report: eLife Assessment

**DOI:** 10.7554/eLife.111164.3.sa0

**Authors:** Rajan Sankaranarayanan

**Affiliations:** Centre for Cellular and Molecular Biology India

**Keywords:** Solid, Valuable

This **valuable** work addresses a longstanding question of how the extant genetic code came to be selected and conserved almost universally across life. Using a mutational approach and a small set of reporters, the authors demonstrate that the mutational impact was similar for non-standard genetic codes. The data provide **solid** support for the claim of having provided experimental verification of the error minimization theory.

# Referee report: Reviewer #1 (Public review)

**DOI:** 10.7554/eLife.111164.3.sa1

[Editors' note: This version has been assessed by the Reviewing Editor without further input from the original reviewers. The authors have addressed the comments raised in the previous round of review satisfactorily and toned down the comments as advised.]

In this manuscript, the authors investigate the relationship between genetic codes and their robustness to single-point mutations. They construct ten alternative genetic codes by reassigning nine codons to Leu, Ser, or Ala, and assess mutational robustness using three reporter proteins subjected to error-prone PCR. This represents an interesting experimental approach to addressing the hypothesis that the standard genetic code is optimized for mutational robustness.

# Referee report: Reviewer #2 (Public review)

**DOI:** 10.7554/eLife.111164.3.sa2

The study addresses the long-standing question in molecular biology and genetics: why has nature selected the current genetic code (SGC, or standard genetic code)? The authors have tested 'error minimization theory', one of the prevailing hypotheses to explain this. Their approach is to create a minimum genetic code (MGC) and its variants (3^9 theoretical possible codes). Using three parameters to quantify the effect of mutations (Polarity, volume, and hydropathy), they computationally test the cost of these genetic codes (3^9) by simulations. Finally, they test this cost experimentally using an in vitro translation system with 10 select genetic code variants with a range of costs (low to high). They use three randomly mutated reporter genes for this purpose - beta-galactosidase, luciferase, and mSG. They find no correlation between the cost of the genetic code and the reporters' output. Based on these observations, they suggest that error-minimization theory may not explain the current egocentric code.

The question they are asking is very exciting, and their approach is solid. The authors are very careful in their analyses and conclusions.

# Referee report: Reviewer #3 (Public review)

**DOI:** 10.7554/eLife.111164.3.sa3

Summary:

In this manuscript, Miyachi and Ichihashi investigate whether the arrangement of the genetic code affects mutational robustness. Using an in vitro minimal genetic code with vacant codons, they constructed 10 non-standard genetic codes by reassigning Ala, Ser, and Leu, generating codes with replacement costs that were generally higher than those of the standard genetic code across several amino acid property measures. They then tested how random mutations affected the activity of reporter proteins translated under these altered codes. Although error minimization theory predicts that higher-cost codes should make mutations more harmful, the authors report that protein function declined to a similar extent across all codes examined, suggesting that mutational robustness remains largely unchanged within the range of genetic code alterations tested here.

Strengths:

This is an interesting study that investigates one of the most fundamental and intriguing questions in molecular evolution: the emergence of the genetic code, which is nearly universal across nature. The in vitro approach is a powerful aspect of the work and provides an opportunity to examine this phenomenon experimentally at a depth that has previously been inaccessible.

# Author comment: Author response

**DOI:** 10.7554/eLife.111164.3.sa4

**Authors:** Norikazu Ichihashi, Ryota Miyachi

**Affiliations:** The University of Tokyo Tokyo Japan; The University of Tokyo Tokyo Japan

The following is the authors’ response to the original reviews.

**Public Reviews:**

**Reviewer #1 (Public review):**

In this manuscript, the authors investigate the relationship between genetic codes and their robustness to single-point mutations. They construct ten alternative genetic codes by reassigning nine codons to Leu, Ser, or Ala, and assess mutational robustness using three reporter proteins subjected to error-prone PCR. This represents an interesting experimental approach to addressing the hypothesis that the standard genetic code is optimized for mutational robustness.

We sincerely thank the reviewer for the positive evaluation of our experimental approach. We are encouraged that the reviewer recognizes the value of constructing multiple non-standard genetic codes in vitro and using them to experimentally examine the relationship between genetic code arrangement and mutational robustness. In the revised manuscript, we have further clarified the scope of our experimental system and the interpretation of the results, particularly emphasizing that our conclusions concern the mutational robustness of individual reporter protein activity measured in an in vitro translation system.

Major comment:

While I find the experimental design valuable, I am not fully convinced by the authors' conclusion that "alterations of the genetic code within the ranges explored in this study have no significant effect on mutational robustness". The current analysis is based on the functional output of three individual reporter proteins. Given that cellular systems involve far more complex interactions, it would be more appropriate to limit this conclusion to mutational robustness at the level of individual protein activity, rather than making broader generalizations.

We thank the reviewer for this important comment. We agree that our original wording was broader than what can be directly supported by the present experiments. Because our analysis is based on the functional outputs of three individual reporter proteins translated in a reconstituted in vitro system, the results do not directly address mutational robustness at the level of the cellular system, protein interaction networks, or organismal fitness.

Accordingly, we have revised the manuscript to limit our conclusion to the mutational robustness of individual reporter protein activity. In the revised Abstract, Results, and Discussion, we now state that within the experimentally tested range of non-standard genetic codes, we did not detect a dependence of the mutation-induced decrease in reporter protein activity on mutational cost. We have also added a statement in the Discussion noting that cellular systems involve many additional layers, including protein–protein interactions, metabolic networks, quality-control systems, and growth selection, and that whether genetic code arrangement affects robustness at these higher biological levels remains an important question for future work.

Specifically, we have added this explanation and the new experiment to the revised manuscript as follows.

Abstract

“This result provides direct experimental evidence that mutational robustness does not significantly change in individual reporter protein activity when the genetic code is altered within the range of mutational cost tested in this study…”

Introduction

“Random mutations decreased reporter protein function at similar levels across all genetic codes examined, implying that alterations of the genetic code within the ranges explored in this study have no significant effect on mutational robustness of individual protein activity.”

Result

“Taken together, these results indicate that mutational robustness of individual reporter protein function did not substantially differ among the genetic codes…”

Discussion

“…suggesting that mutational robustness of protein activity remained largely unchanged within at least the ranges of mutational cost tested in this study. It should be noted that this conclusion is limited to the activity of individual reporter proteins translated in a reconstituted in vitro system. Therefore, whether similar trends would be observed at the level of cellular fitness or long-term evolution remains an open question.”

Specific comments

(1) tRNA modification and expression efficiency (Page 5, line 131)

The authors attribute the observed inefficiency to the lack of chemical modifications in the tRNAs used. However, gene expression efficiency can also be strongly influenced by DNA sequence design. To better support this claim, it would be helpful to compare luciferase activity when expressed using native *E. coli* tRNAs. This comparison could clarify whether the observed effects are due to tRNA modification status or other sequence-dependent factors.

We thank the reviewer for this important suggestion. We agree that the translation efficiency of NanoLuc templates with 21-, 32-, and 46-codons may be affected not only by the chemical modification of tRNAs but also by sequence-dependent factors, such as codon context and mRNA structure.

To examine this possibility, we performed an additional comparison using native *E. coli* tRNAs in the tfPURE system. When the NanoLuc templates encoded with 21, 32, or 46 codons were translated using native *E. coli* tRNAs, the observed luminescence values were 1.2 × 10^10^, 0.78 × 10^10^, and 0.60 × 10^10^, respectively. Thus, the 46-codon NanoLuc template showed lower activity than the 21- and 32-codon templates even with native tRNAs, indicating that sequence-dependent effects indeed contribute to translation efficiency.

However, the difference among these templates with native *E. coli* tRNAs was within approximately two-fold. This effect was much smaller than the marked decrease observed when the 46-codon template was translated using the in vitro prepared 46 tRNAs SGC system. Therefore, while sequence-dependent effects cannot be excluded, the inefficient translation in the reconstructed 46 tRNAs SGC is likely to be mainly attributable to the limited functionality of unmodified tRNAs decoding NNA codons.

We have revised the manuscript to clarify this interpretation and have added the new comparison using native *E. coli* tRNAs.

“We also examined whether the lower translation efficiency of the 46-codon NanoLuc template could be explained by sequence-dependent effects, such as codon context or mRNA structure. When the 21-, 32-, and 46-codon NanoLuc templates were translated using native *E. coli* tRNAs in the tfPURE system (Figure 1–figure supplement 2), the 46-codon template showed lower activity than the 21- and 32-codon templates; however, this difference was within approximately two-fold. Accordingly, we decided to use only the 32 codons used in near-SGC (i.e., excluding NNA codons) in the subsequent construction of non-standard genetic codes.”

(2) Discrepancy between expression level and activity (Figure S7 vs Figure S8).

Although GAL expression levels appear similar across different genetic codes (Figure S7), their activities differ substantially (Figure S8), even in the low-mutation library. This discrepancy warrants further investigation. Possible explanations include differences in protein folding efficiency or translational error rates, as mentioned by the authors in the main text.

To address this, the authors could analyze the protein products using mass spectrometry. If this is not feasible due to low expression levels, alternative approaches such as SDS-PAGE (e.g., with radiolabeling or Western blotting) would still provide valuable information. Additionally, comparing activity after in vitro refolding could help distinguish between folding defects and sequence-level errors. While I understand that the primary aim of this study is to compare mutational robustness across genetic codes, discussing these observations would significantly enhance the mechanistic insight of the work.

We agree that the discrepancy between similar GAL expression levels and different GAL activities across genetic codes is important for interpreting the results.

In our experiment, GAL protein amounts were quantified using a C-terminal HiBiT tag. Because the HiBiT tag was fused to the C-terminus of GAL, this assay indicates that the amount of C-terminally completed GAL products did not differ substantially among genetic codes. However, we agree that this assay does not evaluate the sequence fidelity, amino acid misincorporation patterns, or folding state of the translated products. Therefore, the observed differences in GAL activity despite similar HiBiT signals may reflect genetic code-dependent differences in translational error rates, amino acid misincorporation, protein folding efficiency, or other effects on the fraction of catalytically active protein.

We have revised the Discussion to explicitly describe this interpretation and to clarify that detailed mechanistic dissection of these baseline activity differences, for example by mass spectrometry, SDS-PAGE/Western blotting, or refolding analysis, is an important future direction but beyond the scope of the present study. We also clarified that the main analysis in this study uses the ratio of activity from the high-mutation library to that from the corresponding low-mutation library within each genetic code.

We have added this explanation to the revised manuscript as follows.

“Although protein amounts quantified by the HiBiT tag were comparable among genetic codes, GAL activities differed substantially. This indicates that the activity differences among genetic codes were not primarily attributable to differences in the amount of C-terminally completed translation products. The HiBiT assay does not provide information on the fraction of catalytically active protein, including sequence fidelity or folding state, and therefore cannot distinguish among these possibilities. Detailed characterization of translated products by mass spectrometry would provide further mechanistic insight into how individual non-SGCs affect protein quality. However, the primary objective of the present study was to compare mutation-dependent activity loss across genetic codes. Therefore, we evaluated this effect by normalizing the activity of the high-mutation library to that of the corresponding low-mutation library within each genetic code.”

(3) Protein expression analysis for additional reporters.

Since protein expression levels are critical for interpreting reporter activity, similar analyses should also be performed for luciferase (Luc) and mSG in both high- and low-mutation libraries. This would ensure that differences in activity are not confounded by variations in protein abundance.

We agree that protein abundance is an important factor for interpreting reporter activity. In this study, we performed HiBiT-based protein quantification for GAL because GAL showed the largest variation in absolute activity among genetic codes, even in the low-mutation library. This analysis showed that the amount of C-terminally completed GAL products was broadly comparable among genetic codes and between low- and high-mutation libraries, indicating that the observed GAL activity differences were not primarily attributable to differences in total protein abundance.

For all three reporters, our main analysis was based on the ratio of activity from the high-mutation library to that from the corresponding low-mutation library within each genetic code. This normalization was intended to evaluate mutation-dependent activity loss while reducing the influence of code-specific baseline differences in expression level or protein quality. We believe that the data are sufficient to evaluate the effect of mutations on protein activities. Nevertheless, we agree that protein quantification for Luc and mSG would provide useful information regarding variation in the baseline levels of reporter activity, and this is an important direction for future work.

**Reviewer #2 (Public review):**

Summary:

The study addresses the long-standing question in molecular biology and genetics: why has nature selected the current genetic code (SGC, or standard genetic code)? The authors have tested 'error minimization theory', one of the prevailing hypotheses to explain this. Their approach is to create a minimum genetic code (MGC) and its variants (3^9 theoretical possible codes). Using three parameters to quantify the effect of mutations (Polarity, volume, and hydropathy), they computationally test the cost of these genetic codes (3^9) by simulations. Finally, they test this cost experimentally using an in vitro translation system with 10 select genetic code variants with a range of costs (low to high). They use three randomly mutated reporter genes for this purpose - beta-galactosidase, luciferase, and mSG. They find no correlation between the cost of the genetic code and the reporters' output. Based on these observations, they suggest that error-minimization theory may not explain the current egocentric code.

The question they are asking is very exciting, and their approach is solid. The authors are very careful in their analyses and conclusions.

We sincerely thank the reviewer for the positive assessment of our study and for the helpful suggestions. We are encouraged that the reviewer found the question exciting and the approach solid. In the revised manuscript, we have clarified the rationale for using the MGC/near-SGC framework, added further analyses and explanations of the mutational cost calculations, and revised the wording of our conclusions to more explicitly define the scope and limitations of the present experimental system.

(1) The rationale for using MGC instead of SGC: It is unclear why the authors rely on the MGC for this analysis when the central question concerns the SGC. If the goal is to evaluate whether the SGC minimizes mutational cost, a more direct approach would be to generate alternative variants of the SGC itself and compare their mutational cost distributions. At present, it is difficult to assess whether conclusions drawn from this comparison are fully relevant to the stated biological question.

We thank the reviewer for this important comment. We agree that directly constructing alternative variants of the SGC by changing amino acid assignment from SGC would be the most straightforward approach to testing whether the SGC minimizes mutational cost. However, this approach is currently not feasible in our reconstituted translation system for two reasons.

First, our attempt to construct a 46-tRNA SGC-like system revealed that translation using the 46-codon NanoLuc template was approximately 100-fold less efficient than translation using the MGC or near-SGC (Fig. 1). This low activity likely reflects inefficient decoding of NNA codons by in vitro-prepared tRNAs, which lack native post-transcriptional modifications. Because this system did not provide sufficient translational activity for systematic reporter assays, we restricted subsequent experiments to the 32-codon near-SGC framework, excluding NNA codons. We now describe this technical limitation more explicitly in the revised manuscript.

Second, the MGC framework provides vacant codons that can be reassigned by adding anticodon-variant tRNAs. This feature is essential for constructing multiple genetic code variants in parallel under controlled in vitro conditions. We, therefore, constructed the near-SGC-based non-SGC by adding each tRNA variant to the MGC as an experimentally tractable model system to verify whether differences in genetic code arrangement affect mutation-induced decreases in reporter protein activity.

We have added this explanation to the revised manuscript as follows.

“We first established a minimal genetic code, composed of 21 tRNAs with vacant codons, which allows multiple alternative codon assignments to be introduced under otherwise comparable translation conditions.”

Despite this technical limitation, we believe that the central conclusion of this study—that mutational robustness in individual reporter protein activity does not change significantly when the genetic code is altered within the range of mutational costs tested here—remains well-supported by the present results.

(2) The mutational cost analysis appears biologically oversimplified because all amino acid substitutions are treated equivalently. The analysis assumes that all mutations contribute equally to fitness consequences, which does not reflect biological reality. In natural proteins, the impact of an amino acid substitution depends strongly on its structural and functional context. For example, substitutions affecting catalytic residues, ligand-binding interfaces, phosphorylation sites, or other regulatory motifs can severely impair protein function even when associated changes in polarity, hydropathy, or volume are minimal. Conversely, substitutions in structurally permissive or functionally dispensable regions may have little or no measurable effect despite larger physicochemical differences. Therefore, changes in polarity, hydropathy, and volume alone do not necessarily predict functional consequences.

We agree that the mutational cost used in this study is a simplified measure and does not capture the full biological complexity of amino acid substitutions. As the reviewer pointed out, the functional consequence of a substitution depends strongly on its structural and functional context, including whether the affected residue is involved in catalysis, ligand binding, protein–protein interactions, regulatory motifs, folding, or structurally permissive regions.

In this study, we used physicochemical-property-based mutational costs because this type of definition has been widely used in classical formulations of the error minimization theory. Our aim was therefore not to construct a comprehensive predictor of protein fitness effects, but to experimentally test whether the conventional theoretical cost metrics used to discuss genetic code optimality are reflected in the average mutation-induced decrease in reporter protein activity. We have now clarified this rationale in the revised manuscript.

“It should be noted that this conclusion is limited to the activity of individual reporter proteins translated in a reconstituted in vitro system. Therefore, whether similar trends would be observed at the level of cellular fitness or long-term evolution remains an open question.”

(3) It is not clear why they increased the concentration of the two tRNAs in near-SGC. Have they maintained the same tRNA concentrations in experiments explained in Fig 5 for all 10 genetic codes tested?

We apologize that the rationale for increasing the concentrations of tRNA^Val^_CAC_ and tRNA^Arg^_CCU_ was not sufficiently clear in the original manuscript. As we wrote in the previous manuscript, “To improve translation efficiency with near-SGC, we focused on two tRNA concentrations (tRNA^Val^_CAC_ and tRNA^Arg^_CCU_), which were suggested to have low activities in a previous study (Iwane et al., 2016),” we tested whether increasing their concentrations would improve translation efficiency. As shown in Figure 1–figure supplement 1, NanoLuc activity increased as the concentrations of these two tRNAs were raised and used at 100 ng/µL for tRNA^Val^_CAC_ and tRNA^Arg^_CCU_ in the optimized near-SGC, referred to as near-SGC (RV), and in all subsequent experiments. Additional anticodon-variant tRNAs required for each non-SGC were used at optimized concentrations determined from Figure 2–figure supplement 1. For each genetic code, the same tRNA composition and concentrations were used for the low- and high-mutation libraries (See Supplementary Table S7). To clarify this point, we added the sentence, “The increased concentrations of these two tRNAs were used in all the subsequent experiments,” in the corresponding part.

**Reviewer #3 (Public review):**

In this manuscript, Miyachi and Ichihashi investigate whether the arrangement of the genetic code affects mutational robustness. Using an in vitro minimal genetic code with vacant codons, they constructed 10 non-standard genetic codes by reassigning Ala, Ser, and Leu, generating codes with replacement costs that were generally higher than those of the standard genetic code across several amino acid property measures. They then tested how random mutations affected the activity of reporter proteins translated under these altered codes. Although error minimization theory predicts that higher-cost codes should make mutations more harmful, the authors report that protein function declined to a similar extent across all codes examined, suggesting that mutational robustness remains largely unchanged within the range of genetic code alterations tested here.

Strengths:

This is an interesting study that investigates one of the most fundamental and intriguing questions in molecular evolution: the emergence of the genetic code, which is nearly universal across nature. The in vitro approach is a powerful aspect of the work and provides an opportunity to examine this phenomenon experimentally at a depth that has previously been inaccessible.

Weaknesses:

However, the authors' use of random mutation libraries has certain limitations that prevent the study from realizing its full potential to uncover the mechanisms governing the molecular evolution of the genetic code.

We sincerely thank the reviewer for the positive evaluation of our study and for recognizing the strength of the in vitro approach. We are encouraged that the reviewer considers this system a powerful way to experimentally address the emergence of the genetic code.

We also appreciate the reviewer’s constructive comments regarding the limitations of random mutation libraries. We agree that pooled random libraries do not allow us to assign functional effects to individual mutations or to fully uncover the molecular mechanisms underlying mutational robustness. In the revised manuscript, we therefore clarify that our conclusions concern the library-averaged effects of random mutations on individual reporter protein activity, rather than the effects of specific mutations or cellular-level fitness. To address this limitation, we have added explanations of the scope and limitations of the present approach.

(1) Statistical analyses are missing for several of the manuscript's main claims. This issue applies throughout the paper, including, but not limited to, Figures 1D, 2B, 4B-D, and 5B.

We thank the reviewer for this important comment. We agree that statistical analyses are necessary to support the major claims of the manuscript. We have therefore added statistical analyses appropriate for the purpose and experimental design of each figure.

For Fig. 1D, we performed one-way ANOVA followed by Tukey’s post hoc test on NanoLuc activity to compare translation efficiencies among the MGC, near-SGC, near-SGC (RV), and SGC conditions. This analysis showed a significant overall difference among conditions (one-way ANOVA, p < 0.0001). Tukey’s post hoc test showed that near-SGC was significantly lower than MGC, that near-SGC (RV) significantly improved near-SGC translation, and that near-SGC (RV) was not significantly different from MGC. In contrast, the 46-tRNA SGC remained significantly less efficient than near-SGC (RV). We have summarized the major comparisons in Supplementary Table S8.

For Fig. 2B, we compared NanoLuc activity between the 21-code control and the corresponding 21+1-code condition for each codon reassignment using Welch’s t-test on luminescence. This analysis was added to statistically support whether each anticodon-variant tRNA increased NanoLuc translation from the corresponding reassigned template. The statistical results are summarized in Supplementary Table S9.

For Fig. 4B–D, we converted mutation rates per base to estimated numbers of mutations per gene and performed Spearman’s rank correlation analysis to evaluate whether reporter activity decreased monotonically with increasing mutational load. This analysis showed strong negative monotonic trends between mutation rate (estimated mutation number) and reporter activity for all three reporters (ρ = −0.90 to −1.00), supporting that the random mutation libraries reduced protein activity in a mutation-load-dependent manner.

For Fig. 5B, replicate-level data were available for GAL, and we therefore performed two-way ANOVA using genetic code and mutation level as factors. This analysis detected significant main effects of genetic code and mutation level, indicating that GAL activity differed among genetic codes and decreased in the high-mutation library. However, no significant interaction between genetic code and mutation level was detected, indicating that the magnitude of mutation-induced activity reduction was not strongly code-dependent under the conditions examined.

Finally, because the central claim of Fig. 5C, 5E, and 5G is that mutational cost does not systematically predict mutation-induced activity loss, we performed Spearman’s rank correlation analysis between each mutational cost metric and the high-/low-mutation activity ratio. No significant correlations were detected for any reporter or cost metric (Spearman’s ρ = −0.23 to 0.25), supporting the conclusion that mutational cost did not show a detectable monotonic relationship with mutation-induced activity loss within the tested range.

We have added these statistical analyses to the revised manuscript. The following sentences were added to the figure legends:

Fig. 1

“Statistical comparisons in (D) were performed using one-way ANOVA followed by Tukey’s post hoc test on NanoLuc activity; major comparisons are summarized in Table S8.”

Fig. 2

“For each template, NanoLuc activity in the 21-code and corresponding 21+1-code conditions was compared using Welch’s t-test on luminescence. Statistical results are summarized in Table S9.”

Fig. 4

“Spearman’s rank correlation coefficients were ρ = −0.90 for GAL, ρ = −1.00 for Luc, and ρ = −1.00 for mSG”

Fig. 5

“For GAL activity in (B), two-way ANOVA was performed using genetic code and mutation level as factors. Significant main effects of genetic code and mutation level were detected (both p < 0.0001), whereas their interaction was not significant. For (C), (E), and (G), Spearman’s rank correlation analysis was performed between each mutational cost metric and the high-/low-mutation activity ratio. Statistical details are summarized in Table S10.”

(2) In Figure 2A, the authors modify the NanoLuc gene by reassigning Ala, Leu, or Ser to new codons and elegantly show that the in vitro availability of the corresponding tRNAs is important for protein function. However, the functional importance of the specific modified positions within NanoLuc is not clear. As a result, it is difficult to determine what the expected consequences of these codon changes should be, which in turn limits the interpretation of the observed changes in protein activity. To improve the interpretability of this experiment, the authors should report exactly how many codons were modified in each variant and, ideally, examine the effect of progressively increasing the number of reassigned codons.

We agree that the exact positions and numbers of codon replacements should be clearly reported. In the revised manuscript, we have added a list of the modified amino acid positions. In brief, two Ala codons, three Ser codons, or four Leu codons were replaced with the target vacant codon; the modified positions were Ala16 and Ala120, Ser31, Ser49, and Ser150, and Leu32, Leu67, Leu144, and Leu170, respectively.

We also agree that progressively increasing the number of reassigned codons would provide additional mechanistic insight. However, the purpose of Fig. 2 was to test whether each vacant codon could be decoded by the corresponding anticodon-variant tRNA to produce functional NanoLuc, rather than to analyze the positional contribution of each replacement. We previously performed such progressive codon replacement analysis for one reassigned codon, ACG, in a related study (Miyachi et al., 2025), and the results supported the same qualitative interpretation. Although we did not repeat this progressive analysis for all codons in the present study, we expect that the qualitative interpretation of Fig. 2 would not be substantially changed.

We have revised the figure text to clarify the scope of the experiment and added the detailed codon replacement information.

“(A) Schematic illustration of reassignment experiments. Translation with the original MGC and NanoLuc template is shown at the top for comparison. An example of Ala reassignment to the UUG codon is shown at the bottom. In this example, three Ala codons in the NanoLuc sequence were replaced with one type of vacant codon (e.g., UUG), generating a 21 + 1 (UUG-Ala) codon set. Similar reassignment experiments were performed for three amino acids (Ala, Ser, and Leu) and nine vacant codons. Specifically, two Ala codons (Ala16 and Ala120), three Ser codons (Ser31, Ser49, and Ser150), or four Leu codons (Leu32, Leu67, Leu144, and Leu170) were replaced.”

(3) The calculations presented in Figure 3 raise an interesting conceptual question: why does the near-standard genetic code not exhibit the lowest cost? One possible explanation is that the standard genetic code evolved under multiple competing constraints and is therefore not expected to be optimal for any single cost metric, while still achieving strong overall performance. In this context, it would be informative if the authors combined the three cost measures into a single integrated index and examined whether the near-SGC performs more favorably when all three dimensions are considered together. Such an analysis could add important depth to the study.

We agree that the near-SGC is not necessarily expected to minimize each individual cost metric, because the standard genetic code may reflect multiple competing physicochemical, translational, biosynthetic, and evolutionary constraints rather than optimization of a single property.

To address this point, we added an integrated cost analysis combining the three physicochemical cost metrics, Cost_PR_, Cost_MV_, and Cost_HI_. Because these three metrics have different numerical scales, we normalized each metric before integration. We used two types of integrated indices.

First, for each metric *m* 𝛜 {*PR*, *MV*, *HI*}, we calculated a min–max normalized cost,

$$\displaystyle \operatorname{Cost}_{m_{n o r m}}(C)=\frac{\operatorname{Cost}_{m}(C)-\min _{h \in G}(h)}{\max _{h \in G}(h)-\min _{h \in G}(h)}$$

Where *G* denotes the set of 19,683 candidate non-SGCs generated by assigning Ala, Ser, or Leu to the nine vacant codon boxes. We then defined the integrated min–max cost as

$$\displaystyle \operatorname{Cost}_{\mathrm{int}, \operatorname{minmax}}(C)=\frac{1}{3}\left(\operatorname{Cost}_{P R_{\text {norm }}}(C)+\operatorname{Cost}_{M V_{\text {norm }}}(C)+\operatorname{Cost}_{H I_{\text {norm }}}(C)\right)$$

Second, we calculated a z-score-normalized cost for each metric,

$$\displaystyle Z_{m}(C)=\frac{\operatorname{Cost}_{m_{n o r m}}(C)-\mu_{m, G}}{\sigma_{m, G}}$$

Where *µ_m,G_* and *𝜎_m,G_* are the mean and standard deviation of *Cost_mnorm_* across the candidate non-SGCs. The integrated z-score cost was then defined as

$$\displaystyle C_{\mathrm{int}, \mathrm{z}}(C)=\frac{1}{3}\left(Z_{P R}(C)+Z_{M V}(C)+Z_{H I}(C)\right)$$

Using both integrated indices, the near-SGC ranked first when compared with all 19,683 candidate non-SGCs; in other words, no candidate non-SGC showed a lower integrated cost than the near-SGC. The integrated min–max cost of the near-SGC was 0.01525, whereas the lowest value among candidate non-SGCs was 0.12301. Similarly, the integrated z-score cost of the near-SGC was −2.47947, whereas the lowest candidate value was −1.90838.

We have added this integrated cost analysis as Supplementary Figure 5–figure supplement 7. We have also revised the Discussion to note that the near-SGC does not necessarily minimize every individual physicochemical cost, but performs most favorably when PR, MV, and HI are considered comprehensively. This result is consistent with the idea that the standard genetic code may represent a compromise among multiple constraints rather than optimization of a single physicochemical property.

“We consider that the cost ranges examined in this study represent substantial fractions, especially for MV and HI. Although the near-SGC did not necessarily exhibit the lowest cost for each individual physicochemical metric, this does not mean that it is unfavorable in the multidimensional cost space. Because the SGC may reflect a balance among multiple physicochemical constraints rather than optimization of a single property, we also calculated integrated cost indices by combining Cost_PR, Cost_MV, and Cost_HI after min–max normalization or z-score normalization. In both integrated indices, the near-SGC showed the lowest overall cost when compared with all 19,683 candidate non-SGCs (Figure 5–figure supplement 7), indicating that no candidate non-SGC exhibited a lower combined cost than the near-SGC when the three physicochemical properties were considered comprehensively.”

(4) It is difficult to assess the consequences of the random mutations presented in Figure 4 on reporter gene function based solely on the reported "error rate/base" parameter. In particular, the x-axis in Figure 4B should be converted into the estimated number of mutations per gene. This would make the results more intuitive and would allow the reader to better evaluate the expected degree of disruption to protein function.

We agree that the mutation rate per base alone does not provide an intuitive sense of the expected mutational burden for each reporter gene. We therefore added a second x-axis to Fig. 4B–D showing the estimated number of mutations per gene. This value was calculated by multiplying the mutation rate per base by the coding sequence length of each reporter gene.

We retained the original mutation rate per base axis to preserve the direct link to the sequencing-based mutation rate measurement, while adding the estimated mutations per gene axis to improve interpretability. We have revised the figure and figure 4 legend accordingly.

“The lower x-axis indicates the estimated number of mutations per gene, calculated by multiplying the mutation rate per base by the coding sequence length of each reporter gene.”

(5) A central limitation of the random mutagenesis libraries used in Figure 5, which also underlie one of the manuscript's main claims, is that the exact mutations and their distribution across the reporter genes are not reported. In addition, protein activity is measured only at the level of the entire library, without directly linking individual mutations to their functional consequences. This substantially limits mechanistic interpretation. In my view, this issue can only be addressed convincingly if the authors test a set of defined variants carrying specific mutations and directly evaluate their functional effects.

(6) Related to the previous point, in Figures 5C, 5E, and 5G, the authors present the ratio between low-mutation-rate and high-mutation-rate libraries. However, because each library contains a different collection of mutations, it is unclear what can be inferred from these comparisons. To overcome this limitation, the authors should assess the effects of altered genetic codes on specific, defined mutations rather than on heterogeneous mutation pools alone.

(7) Along the same lines, in Figures 5C, 5E, and 5G, it is unclear why the effects of random mutations would be expected to correlate with the three calculated cost metrics, given that the positions, identities, and functional relevance of the mutations within the genes are not known. Without this information, the biological meaning of these correlations remains difficult to evaluate.

We agree that using pooled random mutation libraries does not allow us to directly link individual mutations to their functional consequences. We also agree that testing defined variants carrying specific mutations would provide a more direct and mechanistic understanding of how each genetic code affects the functional impact of particular amino acid substitutions. However, the purpose of the present study was different from such a defined-variant analysis. Our aim was to experimentally test whether the conventional mutational cost metrics used in error minimization theory predict the average effect of random mutational loads on protein activity. Because these theoretical costs are themselves defined as average expected physicochemical effects over many possible single-nucleotide substitutions, we reasoned that pooled random mutation libraries provide an appropriate first experimental framework to evaluate whether such average-cost metrics are reflected in the average functional output of translated proteins.

We agree that low- and high-mutation libraries do not contain identical sets of mutations. Therefore, the high-/low-mutation activity ratio should not be interpreted as the effect of the same individual variants before and after additional mutations. Rather, it represents the relative reduction in average activity caused by increasing the mutational burden in a heterogeneous mutation pool under each genetic code. We have revised the text to clarify this interpretation.

We also agree that the positions, identities, and functional relevance of individual mutations are not resolved in this pooled assay. This limitation prevents us from assigning mechanistic effects to specific substitutions. At the same time, using a small set of defined variants would introduce its own selection bias, because the conclusions could strongly depend on which mutations and which protein positions were chosen. Therefore, we consider the random-library approach to be a useful first step for testing library-averaged effects, whereas systematically defined variant analysis or genotype-resolved activity assays will be necessary to reveal mutation-specific mechanisms in future studies.

In response to the reviewer’s concern, we have revised the Discussion to explicitly limit our conclusion to library-averaged effects on individual reporter protein activity. We now state that this approach does not identify the functional effects of individual mutations and that future studies using defined variants or high-throughput genotype–phenotype mapping will be required to determine how specific substitutions contribute to genetic code-dependent mutational robustness.

Result

“To estimate the average activity reduction associated with increased mutational burden under each genetic code, we calculated the ratio of activity obtained from the high-mutation library to that from the corresponding low-mutation library and plotted this ratio against each of the three mutational costs (Fig. 5C).”

Discussion

“A further limitation of this study is that the reporter activities were measured at the level of pooled random mutation libraries. Therefore, the high-/low-mutation activity ratio used in this study should be interpreted as the relative reduction in average activity caused by increasing the mutational burden in a heterogeneous mutation pool, rather than as the effect of identical variants before and after additional mutations. This library-averaged approach was chosen because the mutational costs considered here are also defined as average expected physicochemical effects over many possible single-nucleotide substitutions. In addition, because the non-SGCs constructed in this study were generated by reassigning only Ala, Ser, and Leu, the detectable effects may depend on how frequently mutations involving these amino acids occur in each reporter gene and whether the affected positions are functionally important. If genetic code dependent effects are restricted to a small subset of deleterious variants, such effects may be masked in pooled activity measurements. Future studies using defined variants or high-throughput genotype–phenotype mapping assays will be required to determine the mutation-specific and position-specific mechanisms underlying genetic code dependent effects on protein function (Rozhoňová et al., 2024).”

(8) For each mutagenesis library, the number of variants, the average number of mutations per variant, and the distribution of mutation positions should be reported clearly and transparently. These details are important for evaluating the strength of the conclusions.

We agree that a more transparent characterization of the random mutagenesis libraries is necessary for evaluating the strength and limitations of our conclusions.

In the revised manuscript, we have added the estimated number of mutations per gene to the Results section. This value was calculated by multiplying the mutation rate per base by the coding sequence length of each reporter gene. For the high-mutation libraries used in Fig. 5, the estimated numbers of mutations per gene were approximately 8.0 for GAL, 4.5 for Luc, and 3.3 for mSG. We also added position-wise mutation profiles along each reporter gene (Figure 4–figure supplement 2), in addition to the heatmap shown in the original manuscript. These analyses clarify the mutational burden of each library and show that mutations were broadly distributed across the analyzed regions (approximately 300 nt in the middle of each gene) of the reporter genes.

Regarding the number of variants, the translation reactions were performed using 5 nM DNA template in a 5 µL reaction, corresponding to approximately 1.5 × 10^10^ DNA molecules. However, this value represents the total number of DNA molecules introduced into the reaction and does not directly indicate the number of unique full-length sequence variants, because multiple molecules can share the same genotype, and our sequencing analysis was designed to quantify mutation frequencies and positional distributions rather than to reconstruct full-length genotypes of individual library members. Therefore, we do not infer the exact number of unique variants in each library. Instead, we report the average mutation burden and position-wise non-reference rate distributions.

We have revised the Results and added Supplementary Figure 4–figure supplement 2 accordingly.

“For this experiment, two random mutation libraries were used: a low-mutation library prepared using the high-fidelity polymerase and a high-mutation library prepared using Taq DNA polymerase at a Mn^2+^ concentration that yields mutation rates of 0.002 – 0.005 per base (0.0026 for GAL, 0.0027 for Luc, and 0.0048 for mSG, corresponding to approximately 8.0, 4.5, and 3.3 mutations per gene). We also plotted position-wise non-reference rates along the analyzed regions of each reporter gene, confirming that mutations were broadly distributed across the amplicons (Figure 4–figure supplement 2).”

(9) Because only three amino acids were manipulated in the non-standard genetic codes, it remains unclear whether these particular amino acids occupy positions in the reporter proteins that are especially important for function and therefore likely to generate strong phenotypic effects. More broadly, it is not clear whether the assay is sufficiently sensitive to detect the effects of only a subset of deleterious variants within a pooled library. This point should be addressed more explicitly.

We agree that this is an important limitation of the present study. Because our non-SGCs were constructed by reassigning only Ala, Ser, and Leu, the mutation-dependent effects that can differ among genetic codes are limited to mutations involving these reassigned codons or amino acid substitutions affected by these assignments. Therefore, the sensitivity of the assay depends on how frequently such substitutions occur in the reporter genes and whether the affected Ala, Ser, and Leu-related positions are functionally important.

We have revised the Discussion to address this point more explicitly. In the revised manuscript, we now state that the absence of a detectable cost-dependent effect may reflect not only the limited cost range examined, but also the limited set of reassigned amino acids, the position-dependent importance of Ala/Ser/Leu residues in the reporter proteins, and the sensitivity limit of pooled activity measurements. We further note that future studies using genotype-resolved activity assays (defined variants) will be required to determine whether specific amino acid substitutions or specific protein positions exhibit stronger genetic code-dependent effects.

“A further limitation of this study is that the reporter activities were measured at the level of pooled random mutation libraries. Therefore, the high-/low-mutation activity ratio used in this study should be interpreted as the relative reduction in average activity caused by increasing the mutational burden in a heterogeneous mutation pool, rather than as the effect of identical variants before and after additional mutations. This library-averaged approach was chosen because the mutational costs considered here are also defined as average expected physicochemical effects over many possible single-nucleotide substitutions. In addition, because the non-SGCs constructed in this study were generated by reassigning only Ala, Ser, and Leu, the detectable effects may depend on how frequently mutations involving these amino acids occur in each reporter gene and whether the affected positions are functionally important. If genetic code-dependent effects are restricted to a small subset of deleterious variants, such effects may be masked in pooled activity measurements. Future studies using defined variants or high-throughput genotype–phenotype mapping assays will be required to determine the mutation-specific and position-specific mechanisms underlying genetic code-dependent effects on protein function (Rozhoňová et al., 2024).”

**Recommendations for the authors:**

**Reviewing Editor Comments:**

While we suggest that you address all the technical points raised by the reviewers, you may specifically want to limit the conclusion of the study to mutational robustness at the level of individual protein activity, rather than making broader generalizations. Also, the statistical analysis needs to be strengthened, as indicated in the reviews.

We thank the Reviewing Editor for these important suggestions. We agree that the conclusion of the original manuscript was broader than what can be directly supported by the present experiments. In the revised manuscript, we have therefore limited our conclusion to mutational robustness at the level of individual reporter protein activity measured in a reconstituted in vitro translation system. We now explicitly state that our results do not directly address robustness at the level of cellular fitness, protein interaction networks, or long-term evolution.

We have also strengthened the statistical analyses throughout the manuscript. Specifically, we added one-way ANOVA followed by Tukey’s post hoc test for Fig. 1D, Welch’s t-tests for Fig. 2B, Spearman’s rank correlation analyses for Fig. 4B–D and Fig. 5C/E/G, and two-way ANOVA for GAL activity in Fig. 5B. These analyses have been incorporated into the revised Results, figure legends, and supplementary information.

**Reviewer #2 (Recommendations for the authors):**

(1) Discuss other alternative hypotheses if the error minimization theory is unlikely.

We thank the reviewer for this helpful suggestion. We think that the absence of a detectable relationship between mutational cost and reporter protein activity in our assay should not be interpreted as excluding all possible roles of error minimization in the evolution of the genetic code. Our results specifically address one aspect of the error minimization theory: whether physicochemical-property-based mutational cost predicts the average effect of random point mutations on individual reporter protein activity within the experimentally accessible range of non-SGCs tested here.

In the revised Discussion, we have clarified that the organization of the SGC may have been shaped by multiple factors, including robustness to translational errors, historical constraints associated with genetic code expansion, biosynthetic or coevolutionary processes, stereochemical interactions, and the evolvability of proteins. Our results suggest that the contribution of mutational robustness at the level of individual protein activity may be limited within the range examined here, but they do not exclude the possibility that the SGC provides advantages under other forms of error, at the level of translation fidelity, cellular fitness, or long-term evolution.

We have added a short discussion to clarify this point without expanding the scope of the manuscript beyond the present experimental results.

“It should be noted that this conclusion is limited to the activity of individual reporter proteins translated in a reconstituted in vitro system. Therefore, whether similar trends would be observed at the level of cellular fitness or long-term evolution remains an open question. Moreover, our results do not exclude other possible roles of SGC organization. The SGC may have been shaped by multiple factors, including robustness to translational errors, historical constraints during genetic code expansion, biosynthetic or coevolutionary relationships among amino acids, stereochemical interactions, and effects on protein evolvability (Katoh and Suga, 2023; Koonin and Novozhilov, 2017, 2009; Novozhilov et al., 2007; Wong, 2005).”

(2) A brief description of the PURE translation system can be provided for people from outside the field.

We have added a brief description of the PURE system in the Introduction to make the experimental platform more accessible to readers outside the field. Specifically, we now explain that the PURE system is a reconstituted cell-free translation system composed of purified translation factors, ribosomes, aminoacyl-tRNA synthetases, tRNAs, amino acids, and energy-regeneration components. We also clarify that, in this study, we used a tRNA-free version of the PURE system, in which defined synthetic tRNA sets were supplied externally to reconstruct each genetic code.

Introduction

“A representative platform for such reconstitution is the PURE system (Shimizu et al., 2001), a reconstituted cell-free translation system composed of purified translation components, including ribosomes, translation factors, aaRSs, amino acids, and energy-regeneration components. In particular, a tRNA-free PURE system (Miyachi et al., 2022), in which endogenous tRNA activity is minimized and defined tRNA sets are supplied externally, enables genetic codes to be reconstructed by controlling the supplied tRNAs.”

(3) Figure 5D and F - Technical replicates are provided only for GAL. A similar approach should be taken for LUC and mSG.

We agree that replicate-level measurements for Luc and mSG would further improve reliability. However, repeating the full translation experiments for these reporters was not feasible in the current revision, as each experiment requires large amounts of freshly prepared tRNA-free PURE system and multiple defined tRNA mixtures for every genetic code variant tested. Given these material and technical constraints, we were unable to perform additional biological replicates within the scope of this revision. We would like to emphasize, however, that the GAL replicates shown in Fig. 5D and F are fully consistent across independent experiments, providing direct evidence for the reproducibility of the assay itself. Furthermore, the key metric in our analysis, the activity ratio between high- and low-mutation groups within each genetic code, is an internally normalized measure that is inherently less sensitive to between-experiment variability than absolute activity values. The correlation analyses further showed no significant relationship between mutational cost and this ratio across all three reporters, and this conclusion is consistent regardless of which reporter is examined. Together, we believe these results provide a robust basis for the conclusions drawn, even in the absence of full replication for Luc and mSG.

(4) Provide statistical analysis wherever it is relevant (e.g, to support a lack of correlation).

We have strengthened the statistical analyses throughout the revised manuscript. In particular, to support the lack of detectable correlation between mutational cost and mutation-induced activity loss, we performed Spearman’s rank correlation analyses between each mutational cost metric and the high-/low-mutation activity ratio for all three reporters. No significant correlations were detected for any reporter or cost metric. In addition, we added statistical analyses for other relevant figures, including one-way ANOVA followed by Tukey’s post hoc test for Fig. 1D, Welch’s t-tests for Fig. 2B, Spearman’s rank correlation analyses for Fig. 4B–D, and two-way ANOVA for GAL activity in Fig. 5B.

**Reviewer #3 (Recommendations for the authors):**

(1) In line 122, the phrase "as evenly as possible" is ambiguous and should be explained more precisely.

We thank the reviewer for pointing this out. We have revised the phrase “as evenly as possible” to describe the codon design more precisely. Specifically, we now state that the NanoLuc coding sequences were designed so that the codons available in each genetic code were used with minimal differences in codon counts, while preserving the amino acid sequence of NanoLuc.

“For near-SGC and SGC, the NanoLuc coding sequences were designed so that the codons available in each genetic code were used with minimal differences in codon counts, while preserving the amino acid sequence (Fig. 1B, 32 codons and 46 codons).”

(2) For Figure 1D, a Western blot or another protein gel-based assay would be helpful to exclude the possibility that the observed differences arise from variation in translation efficiency rather than differences in protein activity.

We agree that a protein gel-based assay such as Western blotting would in principle allow us to distinguish differences in translated protein amount from differences in specific activity, and we understand why such data would be informative. However, we would like to clarify that the primary purpose of Fig. 1D was to evaluate the overall functional translation output of each reconstructed genetic code, rather than to determine the mechanistic basis of any observed differences. In this context, NanoLuc luminescence serves as an integrated readout of the entire translation process, encompassing both translational efficiency and protein folding/activity. Crucially, regardless of whether the observed differences in NanoLuc luminescence reflect lower protein yield, reduced specific activity, or a combination of both, the conclusion of Fig. 1D remains the same. Although we did not perform Western blotting in this study, we believe that such an analysis would not change this interpretation and that the current data are sufficient to support this conclusion.

(3) The number 3^9 is not immediately intuitive. It would be helpful if the authors also stated that this corresponds to approximately 20,000 possible non-standard genetic codes.

We have revised the text to state both the exact number and the approximate value: 3^9^ = 19,683, approximately 20,000 possible non-standard genetic codes.

(4) The rationale for using the three cost parameters (PR, MV, and HI) should be explained in greater detail. Because these parameters are central to the manuscript, a citation alone is not sufficient. A concise explanation of their biological relevance would improve the clarity and accessibility of the study.

We agree that the biological relevance of the three cost parameters should be explained more clearly. In the revised manuscript, we have added a concise explanation of why polar requirement (PR), molecular volume (MV), and hydropathy index (HI) were used.

These parameters were selected because they have been widely used in theoretical studies of genetic code optimality and represent distinct physicochemical aspects of amino acid substitutions. PR reflects polarity-related interactions and has been a classical metric in error minimization analyses of the genetic code. MV represents side-chain size and steric volume, which could influence packing and structural stability in proteins. HI reflects hydrophobicity, which is closely related to protein folding and hydrophobic core formation. We have also clarified that these metrics are simplified descriptors and do not capture residue-specific structural or functional context, which we now discuss as a limitation of the study.

“PR reflects polarity-related interactions of amino acids and has been used as a classical measure of amino acid similarity in error minimization analyses. MV represents side-chain size and steric volume, which could affect protein packing and structural stability, whereas HI reflects hydrophobicity, which could be closely related to protein folding or hydrophobic core formation.”

(5) In Figure 3, the experimental framework would be easier to follow if the authors included a schematic and data for one representative non-SGC, explicitly illustrating how it differs from the near-SGC with respect to each of the three cost measures.

We agree that showing one representative non-SGC would make the experimental framework and cost calculation more intuitive.

In the revised manuscript, we added a new panel to Fig. 3 comparing the near-SGC with a representative non-SGC. We selected the PR_max_ code as the representative example because it clearly illustrates how reassignment of vacant codon boxes can increase one mutational cost metric relative to the near-SGC. In this panel, we first show the codon assignment schemes of the near-SGC and PR_max_ code in the same genetic-code format used in Fig. 1. We then show the corresponding heatmap representations for the three physicochemical properties used in the cost calculation: polar requirement, molecular volume, and hydropathy index. The Cost_PR_, Cost_MV_, and Cost_HI_ values are shown for each code.

This new panel illustrates how changes in codon assignment are translated into different physicochemical cost landscapes and clarifies how the representative non-SGC differs from the near-SGC with respect to each of the three cost measures.

“To make the design of non-SGCs more explicit, we show one representative non-SGC together with the near-SGC in Fig. 3B. This comparison illustrates how assignment of Ala, Ser, or Leu to the vacant codon boxes changes the three mutational cost metrics, Cost_PR_, Cost_MV_, and Cost_HI_.”

(6) In line 329, the phrase "similar pattern" is ambiguous and should be explained more explicitly.

We have revised the ambiguous phrase “similar pattern” to describe the observation more explicitly. Specifically, we now state that the relative differences in GAL activity among genetic codes observed in the low-mutation library were broadly retained in the high-mutation library, although overall activity decreased.

“For the high-mutation library, GAL activity decreased overall, while the relative differences in activity among genetic codes observed in the low-mutation library were broadly retained.”

(7) Figure S7 appears to be an important control for the experiments shown in Figure 5, and I recommend moving it to the main figures.

We thank the reviewer for this helpful suggestion. We agree that the HiBiT-based quantification of GAL protein amount is an important control for interpreting the GAL activity measurements in Fig. 5, and we appreciate the recommendation to increase its visibility. This analysis shows that the amount of C-terminally completed GAL products was broadly comparable among genetic codes, indicating that the large differences in GAL activity were not primarily attributable to differences in total translated protein amount.

After careful consideration, we have opted to retain this analysis in the supplementary figures because the main focus of Fig. 5 is the relationship between mutational cost and mutation-induced activity loss, quantified by the high-/low-mutation activity ratio. The HiBiT experiment addresses a related but distinct question: whether differences in absolute GAL activity among genetic codes can be explained by differences in protein abundance, and we felt that including it in the main figures might shift the emphasis away from the central message of Fig. 5. Nevertheless, we have added a clear reference to Figure 4–figure supplement 1 in the main text and the figure legend to ensure that readers are directed to this control when interpreting Fig. 5.
